# Electrically controllable superconducting memory effect in UTe_2_

**DOI:** 10.1038/s41586-026-11015-3

**Published:** 2026-09-16

**Authors:** Zheyu Wu, Hanyi Chen, Mengmeng Long, Daniel Shaffer, Dmitry V. Chichinadze, Andrej Cabala, Theodore I. Weinberger, Alexander J. Hickey, Jinxu Pu, Dave Graf, Vladimir Sechovský, Michal Vališka, Gang Li, Rui Zhou, F. Malte Grosche, Alexander G. Eaton

**Affiliations:** 1 Cavendish Laboratory, University of Cambridge, Cambridge, UK; 2 National High Magnetic Field Laboratory, Tallahassee, FL USA; 3 Department of Physics, University of Wisconsin–Madison, Madison, WI USA; 4 Department of Physics, Washington University in St. Louis, St. Louis, MO USA; 5 Department of Condensed Matter Physics, Faculty of Mathematics and Physics, Charles University, Prague 2, Czech Republic; 6 School of Physics and Astronomy, Shanghai Jiao Tong University, Shanghai, China; 7 Clarendon Laboratory, Department of Physics, University of Oxford, Oxford, UK; 8 Beijing National Laboratory for Condensed Matter Physics, Institute of Physics, Chinese Academy of Sciences, Beijing, China; 9 School of Physical Sciences, University of Chinese Academy of Sciences, Beijing, China

**Keywords:** Superconducting properties and materials, Phase transitions and critical phenomena, Quantum fluids and solids

## Abstract

Multiphase superconductors—materials that host two or more distinct superconductive phases—are exceptionally rare. Examples include heavy-fermion CeRh_2_As_2_ alongside some uranium compounds such as UPt_3_ and URhGe (refs. ^1,2,3^). In the multiphase *p*-wave superfluid ^3^He, complex vortex dynamics can occur at the phase boundary between the A and B phases^4,5^. Here we study the *p*-wave superconductor candidate UTe_2_ (refs. ^6–8^). On applying a magnetic field to access an intermediate regime straddling two distinct superconducting phases^9,10^, we find that direct current pulses can push the material in and out of a metastable state that has an enhanced critical current density *J*_c_. This switching is controllable by the strength and duration of the stimuli, with the system ‘remembering’ whether it is in the high or low *J*_c_ state for extended periods. We interpret this phenomenology to be due to the quenching of a disordered out-of-equilibrium glassy vortex state under perturbation, which has stronger pinning forces and thus higher *J*_c_. The equilibrium vortex lattice is reattained by annealing the system with a gradual current ramp, returning it to the original state. Rather than requiring proximate magnetic or semiconducting interfaces^11–14^, this memory functionality seems to be an intrinsic property of UTe_2_ rooted in the superconducting order itself. Our findings underscore the rich complexity of multiphase quantum vortex matter.

## Abstract (web summary)

Observations of a memory effect in the *p*-wave superconductor candidate UTe_2_, which can be controlled by varying the strength and duration of the electrically applied stimuli, are described.

## Main

If a computer could be assembled from superconducting components, the energy efficiency would far surpass that of conventional electronics. Historic research efforts towards this goal yielded pivotal breakthroughs in the development and discovery of scanning tunnelling microscopy^15^ and high-temperature superconductivity^16^. Although recent strides have been taken in advancing superconducting rectification^17,18^ and switching^19^ technologies, realizing read/writable memory functionality in superconducting platforms has remained challenging.

Encodable superconducting memory functionality has been demonstrated in ferromagnet–superconductor heterostructures^12,13^, as well as through history-dependent trapping of magnetic flux^20,21^. In some type II superconductors such as NbSe_2_, a hysteretic modulation of the critical current density *J*_c_ has been observed near a so-called ‘peak-effect’^22^ region in which *J*_c_ suddenly increases on approaching the upper critical field. Such behaviour is understood to be governed by a dynamic competition between the injection of a highly disordered, strongly pinned vortex phase through surface edge barriers and the subsequent annealing of this disorder by a bulk transport current^23^. By tuning the amplitude, frequency and direction of the driving current, the spatial footprint of this disordered vortex matter may be programmably manipulated^24^. The material effectively archives its electrical history within this spatial distribution, giving the vortex landscape the ability to effect non-volatile information storage^25^.

Here we study vortex dynamics within the multiphase spin-triplet superconductor candidate UTe_2_ (refs. ^6–8^). In a specific portion of the complex phase landscape, we observe hysteretically tunable *J*_c_ properties analogous to those of NbSe_2_ (ref. ^23^). However, rather than becoming pronounced near to the critical temperature, as per the peak effect, in UTe_2_, this ‘memory effect’ manifests at very low temperatures over a magnetic field interval in which this material is known to transition between two distinct superconducting phases^9,10^. We therefore posit that, while in NbSe_2_ thermally induced competition between elastic and pinning energies yields metastable memory phenomenology^23^, by contrast, in UTe_2_, it seems that competing interactions between two distinct vortex structures—each native to their separate superconducting phases—drives the observed superconducting memory effect.

## Multiphase superconductivity

**Fig. 1:**
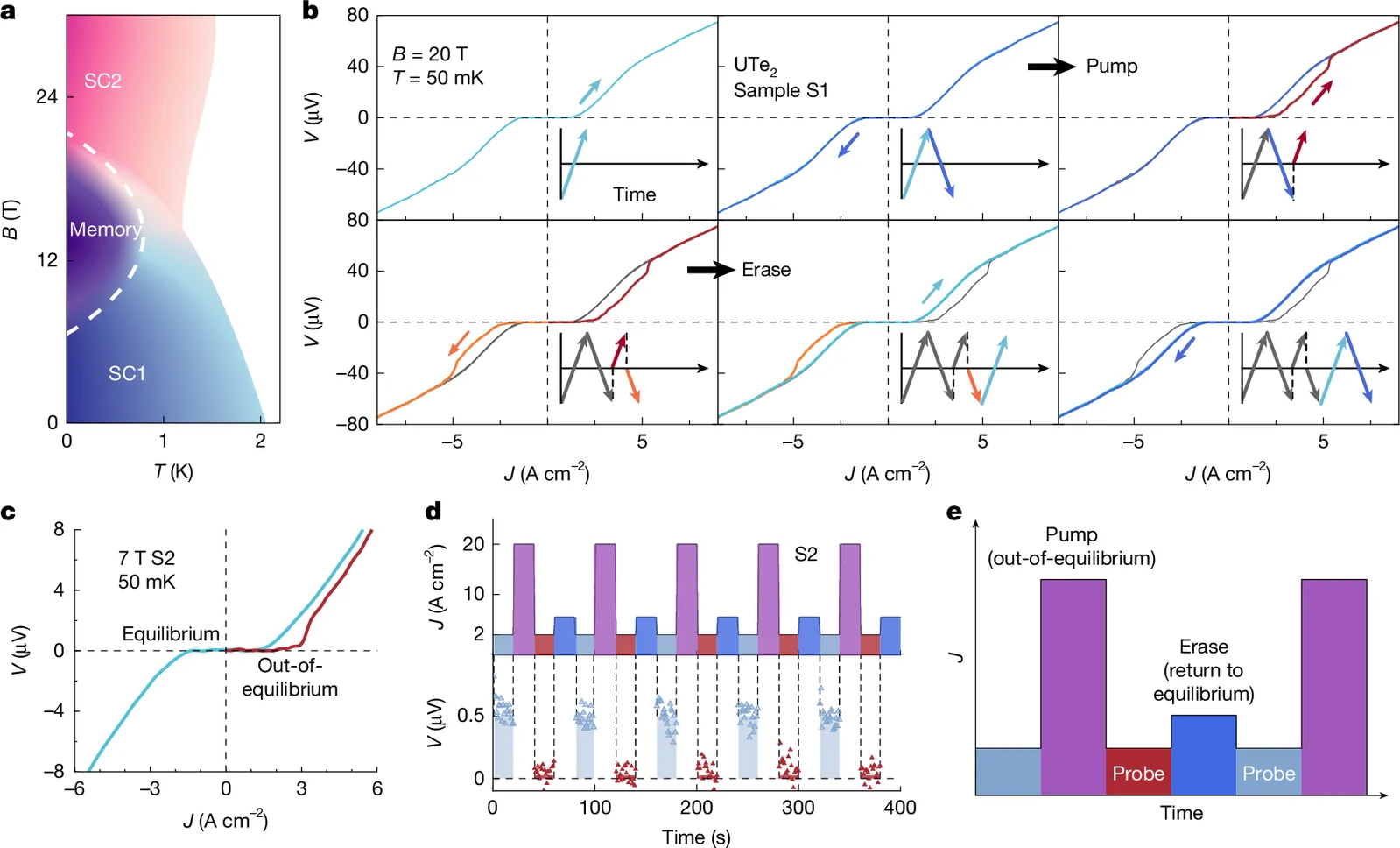
Electrical switching of an intrinsic superconducting memory effect. **a**, The low-temperature phase diagram of UTe_2_ for magnetic field *B* applied along the hard crystallographic $\hat{b}$ direction^31^. The region of phase space in which memory effects are manifested is coloured purple, lying between two distinct superconducting phases (SC1 and SC2). **b**, The measured voltage *V* across a UTe_2_ single crystal as a function of dc current density *J*. Panels are arranged chronologically from left to right, with the insets (and colour coding) showing how *J* was modulated as a function of time. When *J* is ramped smoothly, the profile of *V*(*J*) is in its equilibrium state and no hysteresis is observed. By contrast, after a discontinuous change in *J* from a large magnitude abruptly to zero (labelled ‘Pump’), hysteresis in *V*(*J*) is observed, plotted in red and orange. Smoothly sweeping the current from a large negative value to zero (‘Erase’) then resets the system to the original *V*(*J*) profile with lower *J*_c_. All data were acquired at 50 mK with $B\Vert \hat{b}$. **c**, Data collected on sample S2 at 7 T and 50 mK. Here we plot just two traces, to highlight the hysteretic out-of-equilibrium form of *V*(*J*) in the memory state. **d**, The lower part shows *V* measured as a function of time for modulations of *J* depicted in the upper part, cycling through a sequence of perturbation, measurement and erasure (resetting) protocols, as described in **e**. By sitting at this point in the *J*–*V* curve (sourcing *J* = 2 Acm^−2^), successively switching in and out of the memory state yields a voltage versus time profile similar to supercurrent rectification by the superconducting diode effect^17^. Source data

UTe_2_ is an orthorhombic heavy-fermion superconductor, which, under various conditions of applied pressure and magnetic field, has been reported to have up to six different superconducting phases^8,9,26–37^. The most easily accessible transition between two superconducting states is found at ambient pressure for a magnetic field *B* aligned along the hard $\hat{b}\mathrm{-axis}$. Bulk-sensitive specific heat measurements^9^ have discerned a thermodynamic phase boundary between two superconducting states at around 15–20 T. These superconductive phases are typically referred to as SC1 (the low-*B* state) and SC2 (at higher *B*; Fig. 1a; ref. ^31^). There is a substantial, growing body of evidence that SC1 and SC2 have distinct order parameters^8–10,34,37–39^.

Recent measurements of the surface properties of the SC1 state at low *B*, made by scanning tunnelling microscopy and scanning SQUID, have revealed several anomalous vortex features. These include a mirror-asymmetric profile of the vortex cores^40^, which form in doublets^41,42^, with a tendency to organize into extended stripes of vortices^43^. These vortex structures form for *B* > *B*_c1_ ≈ 4 mT (ref. ^44^) and, so far, have been found to persist up to at least 8 T (ref. ^41^).

The vortex behaviour at higher *B* is also unusual. Current-dependent magnetotransport measurements have discerned a region of anomalously low *J*_c_ between the SC1 and SC2 states at around 15–20 T (ref. ^45^). Alongside signatures in the magnetic susceptibility, these observations have been taken to indicate a region of coexistence between the SC1 and SC2 states^10^ that, hereafter, we refer to as the SC1.5 regime.

We investigated the current–voltage (*J*–*V*) characteristics of UTe_2_ in this region of phase space in which SC1 and SC2 show signs of coexistence. When the material is entirely in either the SC1 or the SC2 state, we observe forms of *V*(*J*) that are typical for a type II superconductor in a magnetic field. By contrast, in the intermediate SC1.5 region, we find that abrupt perturbative modulations of *J* lead to unusual hysteretic features in the form of *V*(*J*), increasing the value of *J*_c_. The system then stays in this new hysteretic high-*J*_c_ state on subsequent measurements, only returning to the original *V*(*J*) profile after the application of a sufficiently large perturbation that acts to reset the system. This hysteretic tuning seems robustly long-lived (persisting over a timescale of at least several hours), constituting a previously unknown electrically controllable superconducting memory effect.

## Superconducting memory effect in UTe_2_

We plot a chronological sequence of *J*–*V* curves in Fig. 1b. Throughout this study, all excitations were applied as direct currents (Methods), unless otherwise specified. With UTe_2_ in its intermediate SC1.5 state at low temperatures, when the current is swept smoothly, *V*(*J*) is a single-valued function, with no hysteresis observed for increasing or decreasing current ramps. By contrast, after suddenly decreasing *J* from a high value down to zero, a larger value of *J*_c_ is then observed on smoothly sweeping *J* back up again (red curve, top-right panel of Fig. 1b). This hysteretic new form of *V*(*J*) is also manifested for opposite polarity current ramps (orange curve, subsequent panel). Then, on smoothly sweeping down from a large magnitude of current back to zero, the initial state is recovered, with the original form of *V*(*J*) retraced for subsequent rising and falling current sweeps (blue curves). We identify this hysteretic tuning of *J*_c_ as a current-controlled memory effect and refer to the hysteretically higher *J*_c_ value as the characterization of the system being in the ‘memory state’.

In Fig. 1c, we show data measured on a second sample (labelled S2). For clarity, here we show just the non-hysteretic *V*(*J*) curve (in blue, labelled ‘Equilibrium’) and the post-perturbation memory state curve (in red, labelled ‘Out-of-equilibrium’). On successive measurements, we find that the hysteretic form of *V*(*J*) in the memory state persists over a timescale of at least several hours. Therefore, although the memory state is away from the equilibrium state of the system, it does not seem to be transiently metastable in character, giving promise for possible future development of non-volatile memory functionalities.

The tunability of the memory state enables us to perform pump/probe operations to switch between different states (Fig. 1d,e). For example, if we choose a *J* value that lies at the bottom of the hysteresis loop, then perturbing the system to switch from the equilibrium (non-memory) state to the out-of-equilibrium (memory) state changes *V* between non-zero and zero values by raising *J*_c_ (blue and red data points in Fig. 1d). These high and low states could, for instance, be labelled 1 and 0 for computational memory purposes.

**Fig. 2:**
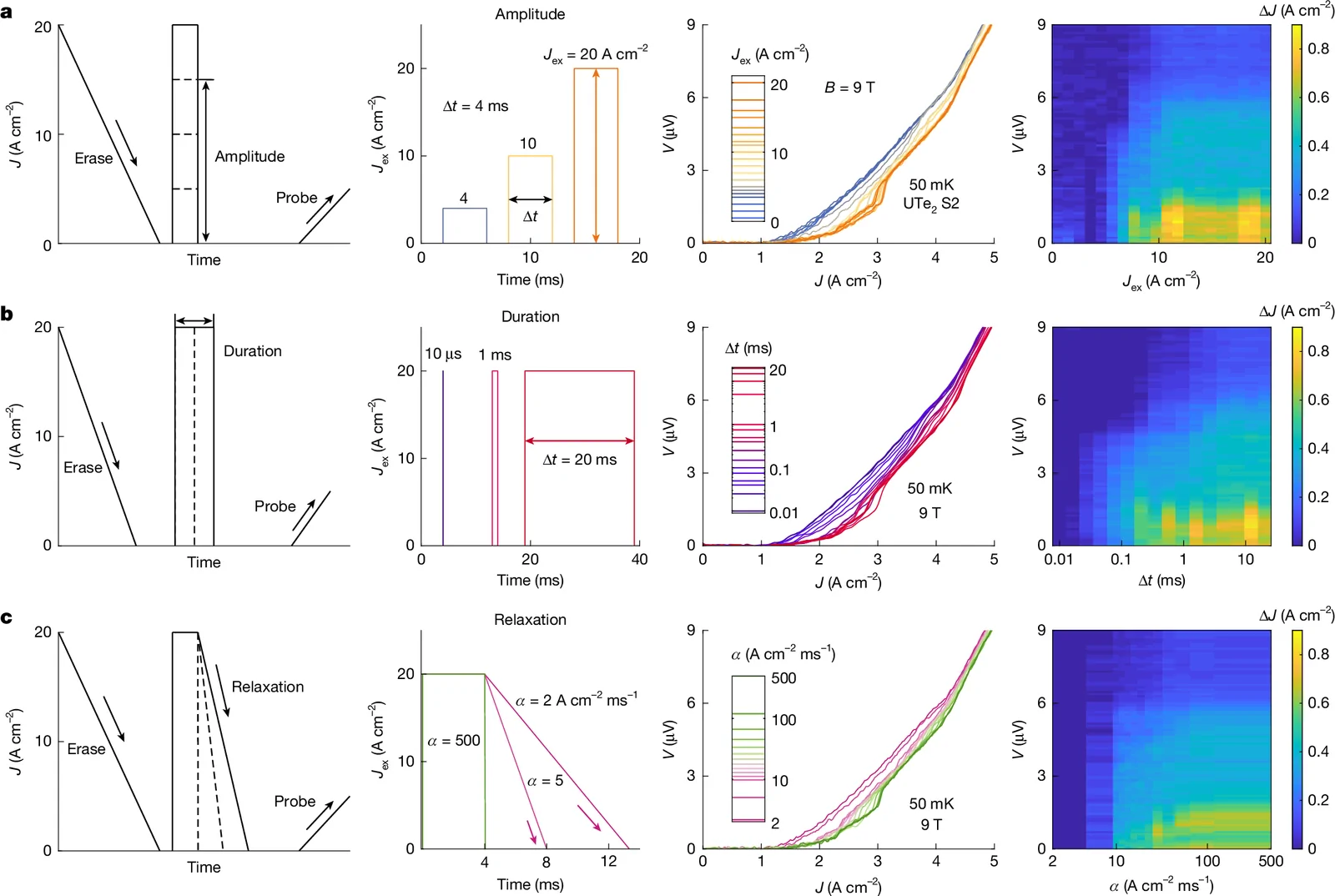
Three tuning parameters to control the memory effect. **a**–**c**, Hysteretic response profiles for modulations of an applied dc excitation’s amplitude (**a**), duration (**b**) and relaxation rate (**c**). In each case, the greater the perturbation to the system—be it a larger amplitude *J*_ex_, longer duration Δ*t* or more rapid relaxation rate *α*—the greater the resulting hysteresis loop in *V*(*J*), up to some saturation value (see Extended Data Fig. 1 for further analysis). For the heatmaps, Δ*J* is calculated as the change in *J* at fixed values of *V*. The regions of highest Δ*J*, coloured yellow, correspond to the strongest memory effect. Source data

**Fig. 3:**
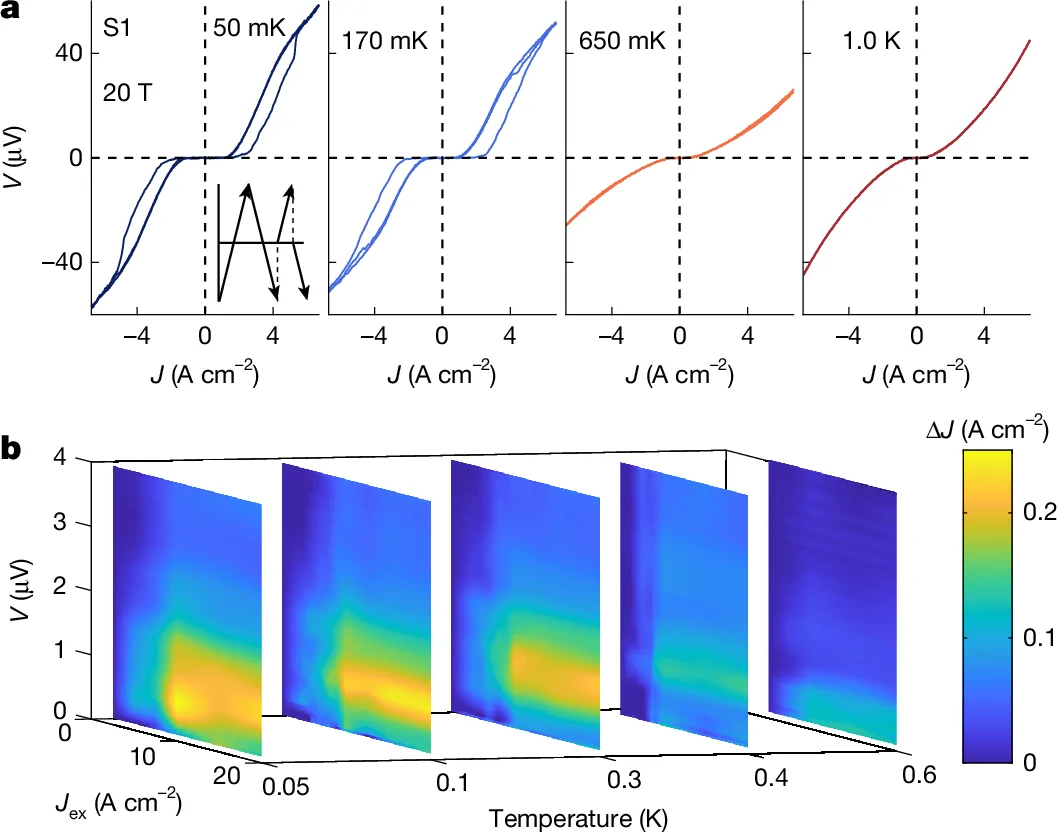
Temperature dependence of the memory effect. **a**, *V*(*J*) at incremental temperatures as indicated. The inset shows the sequence of current modulations. Although large hysteresis loops are recorded at low temperature, these have closed by 650 mK. At 1.0 K, the curvature of *V*(*J*) is markedly different compared with the equilibrium curve at 50 mK (see also Supplementary Fig. 7). **b**, Heatmaps of Δ*J* at incremental temperatures for modulations of the excitation amplitude *J*_ex_ up to 20 A cm^−2^ measured on sample S2 at 14 T. Again, yellow colouring indicates the strongest memory effect, which sharply diminishes at higher temperatures. Source data

**Extended Data Fig. 1:**
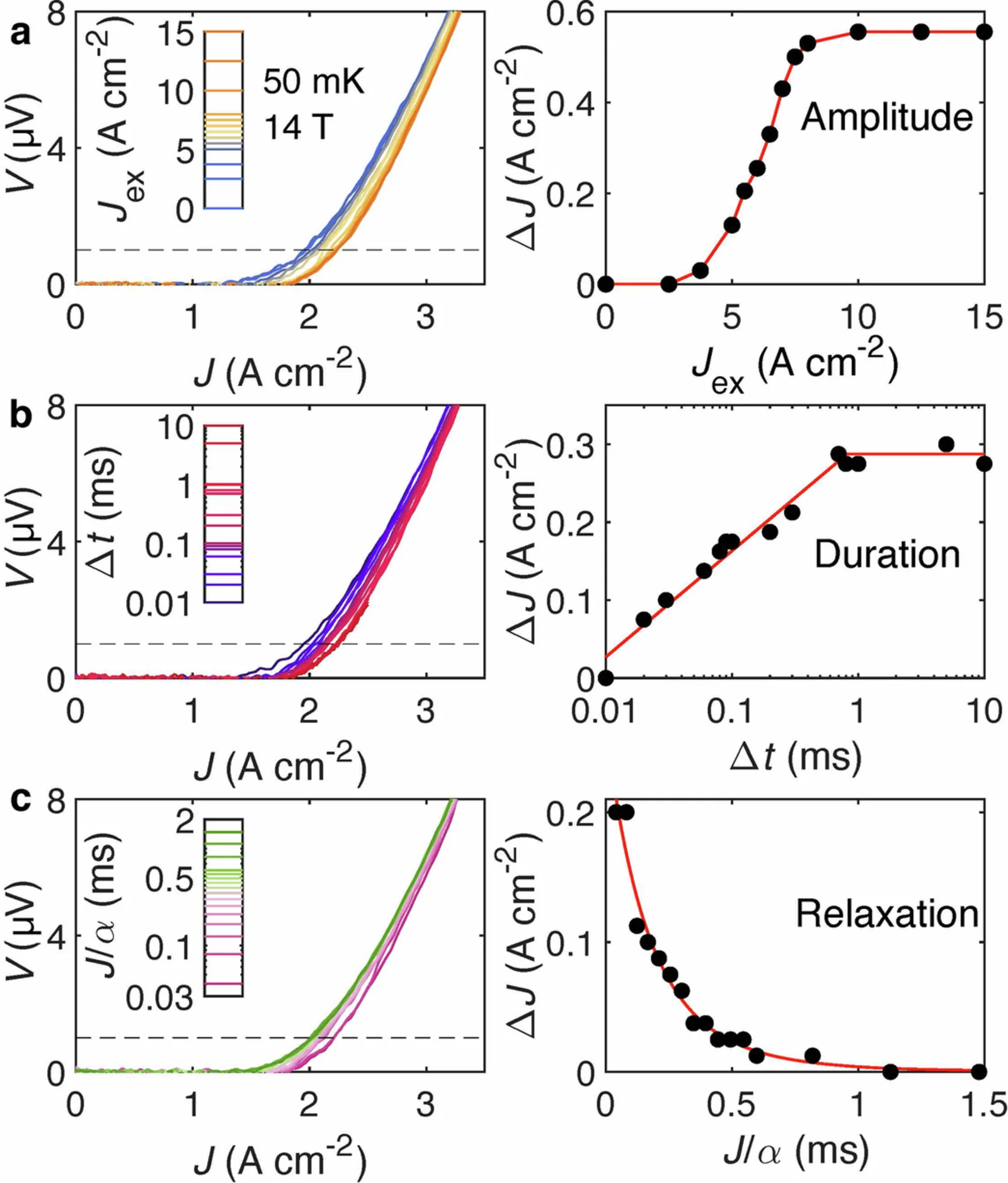
Saturation of the memory effect. **a**–**c**, Amplitude (**a**), duration (**b**) and relaxation rate tuning (**c**) of the memory effect of UTe_2_. For sufficiently large perturbative amplitudes *J*_ex_ or durations Δ*t*, the memory effect approaches a saturation value. Δ*J* is calculated here at a nominal fixed value of *V* = 1 μV, as indicated by the dashed lines. All data were acquired on sample S2 at 14 T and 50 mK.

**Extended Data Fig. 2:**
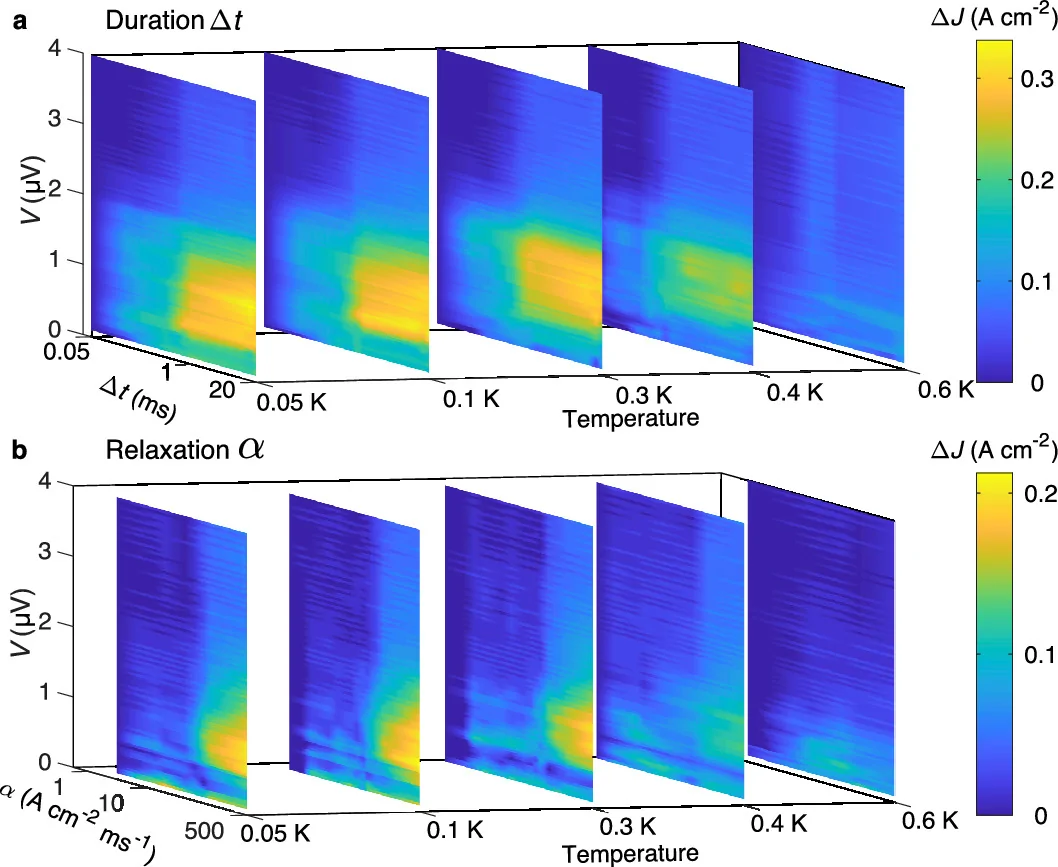
Further temperature dependence of the memory effect. **a**,**b**, Results for duration tuning (**a**) and relaxation rate tuning (**b**), to complement the amplitude tuning depicted in Fig. 3. A similar evolution, of diminishing memory effect at higher temperatures, is seen for all three types of perturbative stimuli.

We find that the memory effect can be controlled by tuning the system in three different ways: by modulating the amplitude, duration and relaxation rate of the perturbative excitation current *J*_ex_ (Fig. 2). For each of these tuning parameters, we find that larger hysteresis loops are induced for stronger perturbations, up to some saturated maximal values (Extended Data Fig. 1). Furthermore, each of these tuning axes yields a similar evolution in temperature of the memory effect. Figure 3 depicts the temperature *T* dependence over the interval 50 mK ≤ *T* ≤ 1.0 K for amplitude tuning. At low temperatures below 0.4 K, bright yellow regions in Fig. 3b identify a strong memory effect characterized by the observation of a large Δ*J* for high *J*_ex_. However, at higher temperatures, this quickly diminishes, with hysteresis loops closing for *T* > 0.6 K. Similar behaviour is also observed under duration and relaxation tuning, as shown in Extended Data Fig. 2. Such a low temperature scale of the memory effect in UTe_2_ is surprising, given that the superconducting *T*_c_ = 2.1 K in zero field and is still >1 K in this field range. This points to some intrinsic low-energy phenomenon, distinct from the superconductivity itself, being responsible for the observed hysteretic *V*(*J*) phenomena.

## Mapping the memory effect

**Fig. 4:**
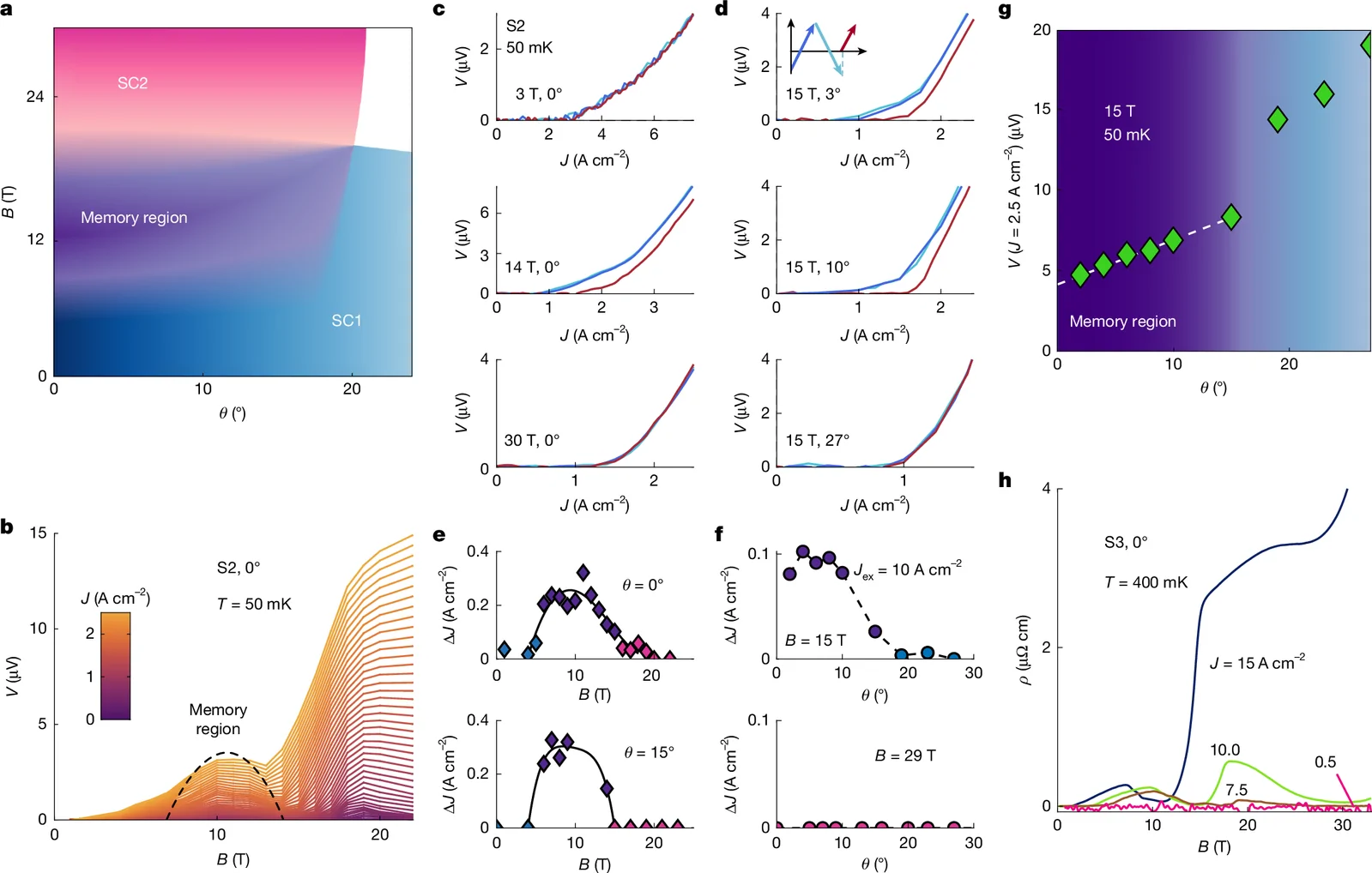
Mapping the memory region between SC1 and SC2. **a**, Schematic phase landscape of UTe_2_ for rotations of *B* by an angle *θ* from $\hat{b}$ towards $\hat{c}$. The memory region (coloured purple)—identified by non-zero Δ*J* at low *T*—is located at the intersection of the SC1 and SC2 domains. We refer to this as SC1.5. **b**, The equilibrium profile of *V*(*B*) at 50 mK for successive *J* values, as indicated by the colour scale. An anomalous non-monotonic kink in *V*(*B*) at high *J* is observed at the boundary of the memory region. **c**, Sequential modulations of *J* at different fields for $B\Vert \hat{b}$ (0°). **d**, Modulations of *J* at different magnetic field tilt angles *θ* at *B* = 15 T. The inset defines the measurement protocol. **e**,**f**, Evolution of Δ*J* as a function of *B* at 0° and 15° (**e**) and as a function of *θ* at 15 T and 29 T (**f**). **g**, Equilibrium *V*(*θ*) for *J* = 2.5 A cm^−2^, *B* = 15 T, *T* = 50 mK measured on sample S2. The transition from the memory region into SC1 is marked by a sudden jump in *V* at around *θ* = 20°, indicating a change in the vortex properties. This angle is where, in panel **f**, Δ*J* reaches zero within the resolution of the measurement. **h**, Magnetic field sweeps of the effective resistivity *ρ* for incremental *J* values as indicated. The data in this panel were acquired by low-frequency ac measurements, whereas all other data in this article are from dc measurements (Methods). Although zero resistance is observed over all *B* in the low *J* limit, at higher *J*, a complex profile of flux-flow behaviour is exhibited. Source data

**Extended Data Fig. 3:**
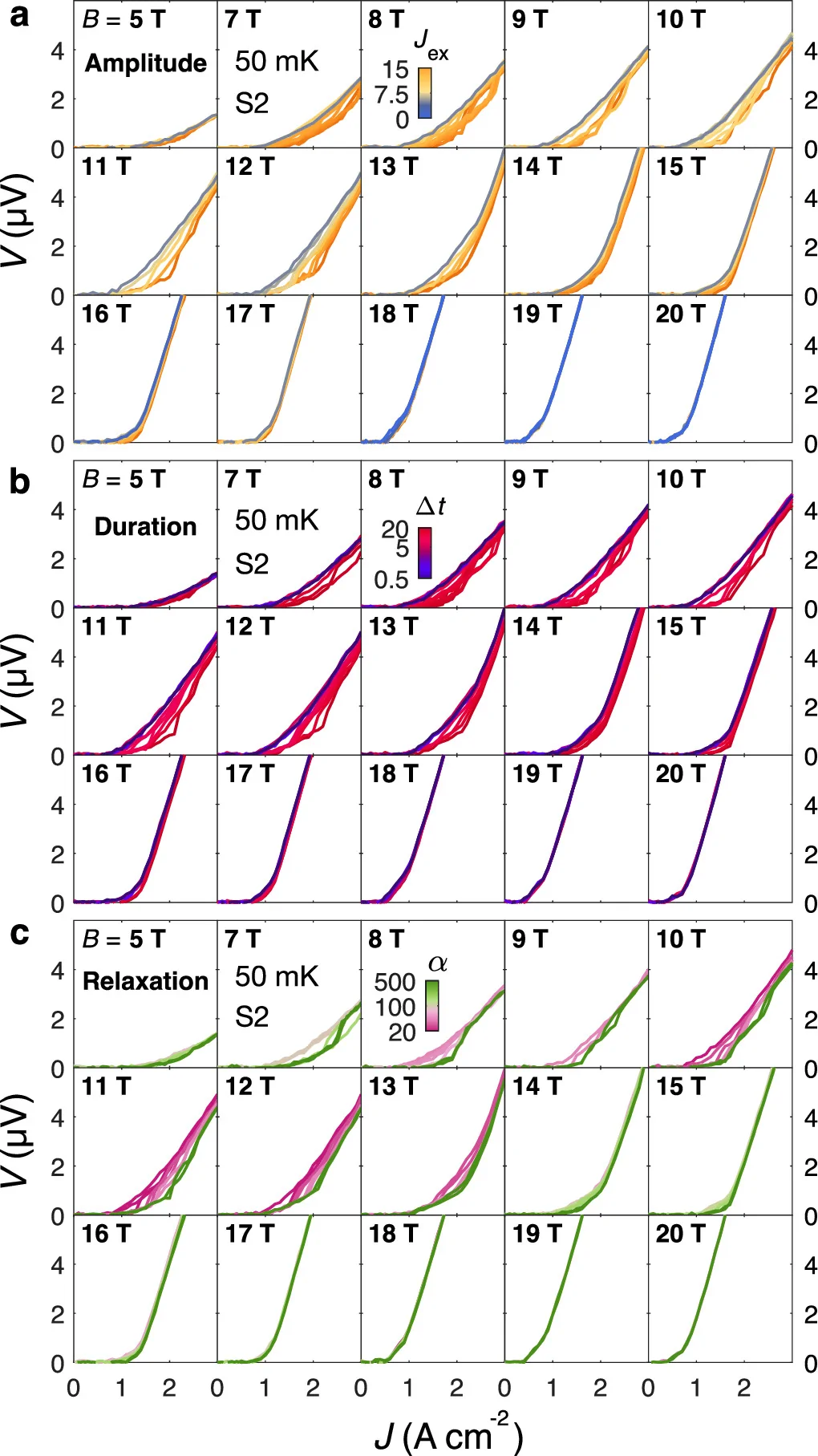
Evolution of the memory effect as a function of magnetic field strength. **a**–**c**, Amplitude (**a**), duration (**b**) and relaxation rate tuning (**c**) of the memory effect. Measurements were performed on sample S2 for *B* aligned along the $\hat{b}\mathrm{-axis}$.

**Extended Data Fig. 4:**
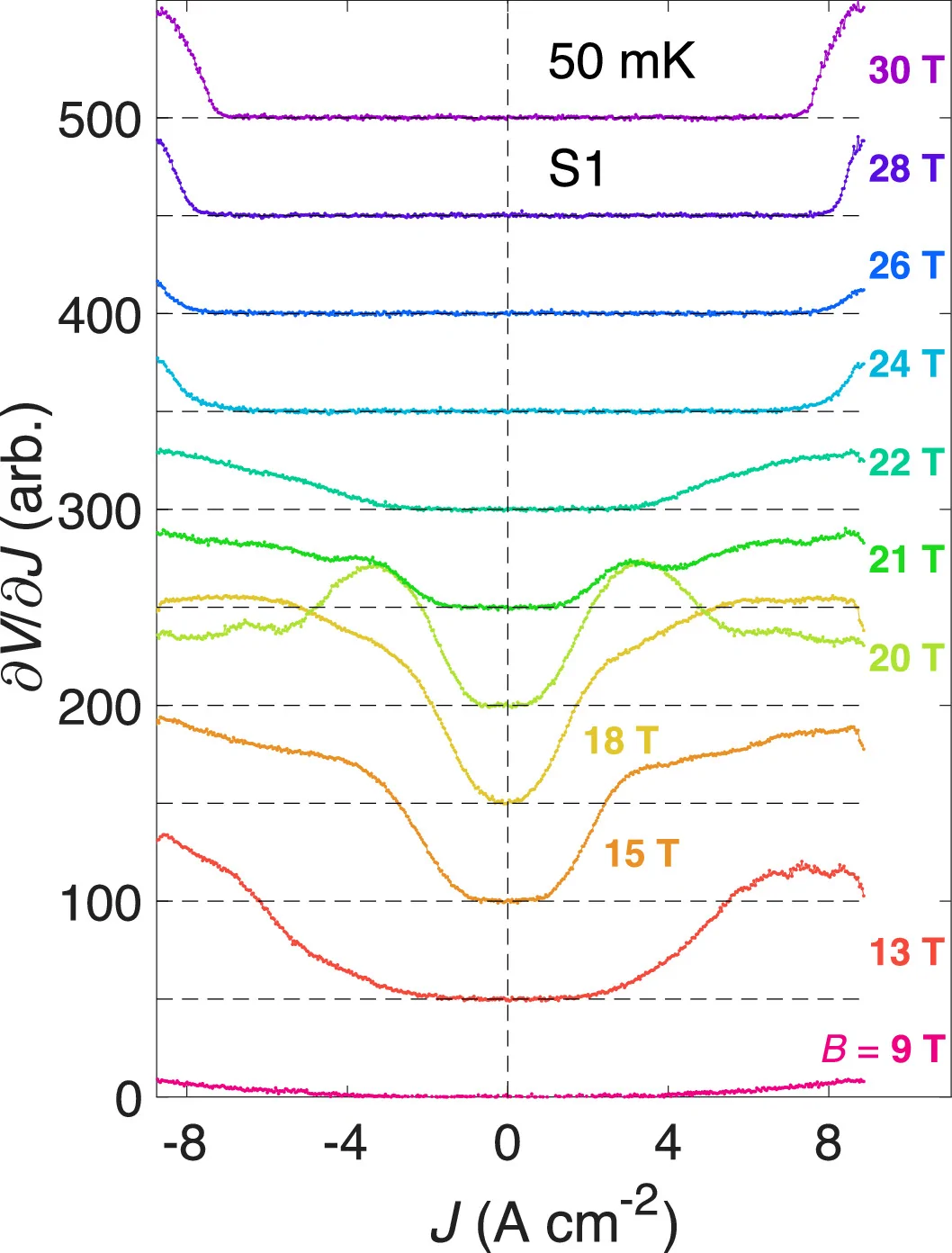
Magnetic field dependence of the slope of *V*(*J*) in the equilibrium state. $\frac{\partial V}{\partial J}$ at incremental field strengths as indicated. *J*_c_ is minimal around 20 T and is actually higher at 28 T than it is at 9 T. Anomalous non-monotonic behaviour is observed in the memory region around 20 T. Note that, for this sample (S1), the memory region is located over a higher range of *B* than for sample S2, probably because of differences in crystalline quality (and hence vortex pinning forces). All measurements were performed at *T* = 50 mK.

To reveal the full domain of phase space in which memory effects are present, we performed a detailed mapping of the hysteretic *V*(*J*) characteristics of UTe_2_ as a function of magnetic field strength and rotation angle up to 30 T at 50 mK, which is summarized in Fig. 4. At low *B*, the form of *V*(*J*) is single-valued and does not exhibit hysteresis under perturbative amplitude, timescale or relaxation rate tuning. As *B* is increased, hysteretic signatures of the memory effect start to appear at around 7 T, which persist up to higher *B* until the phase boundary out of SC1 into SC2 is crossed. The exact value of *B* at which this occurs varies slightly from sample to sample, depending on crystalline quality^9,31^—for sample S1, this is just above 20 T, whereas for S2, it is just below 20 T (Fig. 4e and Extended Data Figs. 3 and 4). At high *B* around 30 T, the material is then fully in the SC2 phase and the memory effect is no longer exhibited (Fig. 4c).

**Extended Data Fig. 5:**
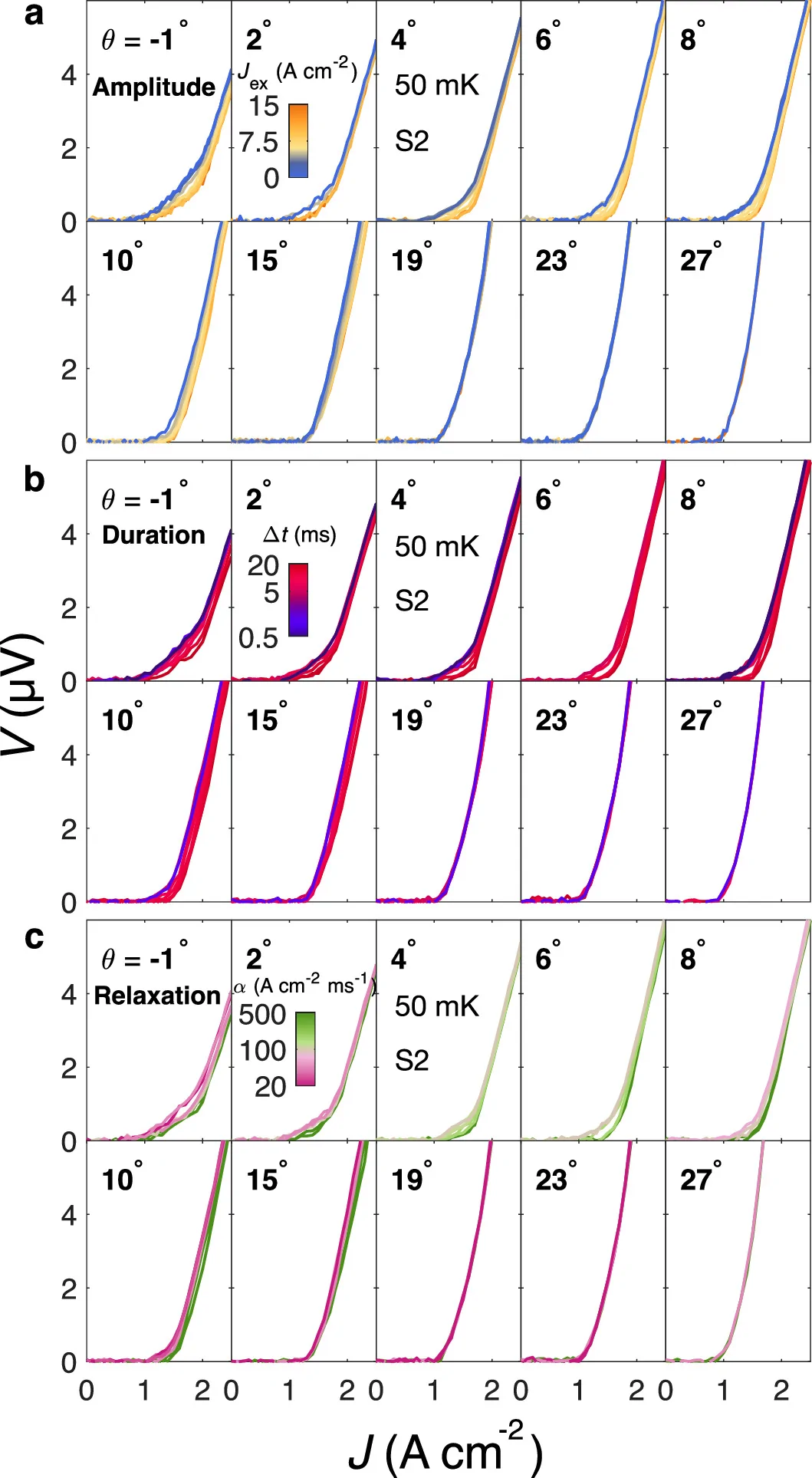
Rotation study of the superconducting memory effect. **a**–**c**, Amplitude (**a**), duration (**b**) and relaxation rate tuning (**c**) of the memory effect under rotations of *B* from the $\hat{b}\mathrm{-axis}$ (*θ* = 0°) towards the $\hat{c}\mathrm{-axis}$ (*θ* = 90°). Δ*J* abruptly diminishes for *θ* ⪆ 19°.

Tilting *B* provides insight into the character of the memory effect. The SC2 phase of UTe_2_ only resides in a narrow range of *θ* close to $B\parallel \hat{b}$, which, for high-quality salt-flux-grown crystals such as those studied here, extends out to *θ* ≈ 20° (ref. ^31^). We find that the memory region closely tracks this. In Fig. 4e,f, we plot the value of Δ*J* observed for a perturbative *J*_ex_ for various *B* and *θ* (see Extended Data Fig. 5 for raw *V*(*J*) curves). At 0°, no change in Δ*J* is observed at low *B*, which quickly rises for *B* > 7 T and peaks just above 0.2 A cm^−2^ at around 10 T. A very similar profile is exhibited at *θ* = 15°, indicating that the strength of the memory effect is not strongly peaked at the $\hat{b}\mathrm{-axis}$ but is similar in magnitude throughout the *B*–*θ* parameter space directly below SC2. Then, on rotating beyond the range of SC2, Δ*J* abruptly diminishes back to zero (Fig. 4f). This truncation of the memory region is also reflected in the variation of *V* measured in equilibrium for a fixed *J* (Fig. 4g), which suddenly jumps on departing the memory region into exclusively SC1 close to *θ* = 20°. The close connection between the angular range in which SC2 sits above SC1, and the observed domain of phase space that hosts the memory effect, underscores that the interplay of these two distinct superconducting states is imperative in generating the observed hysteretic *V*(*J*) phenomena.

## Discussion

Now we turn to considering the likely microscopic origin of the memory effect in UTe_2_. First, given that we are applying substantial electrical current pulses at dilution fridge temperatures, we should consider any extrinsic heating effects that might be present. In the Supplementary Information, we plot the measured temperature as a function of time during the current pulse measurement protocols and present further discussion as to why we do not believe that Joule heating effects greatly affect our measurements. One strong argument against Joule heating artefacts comes from the sensitivity of the memory effect to the relaxation rate of the dc stimulus. In Fig. 2, we showed that stronger memory effect correlates with larger amplitude and duration tuning. For both of these, the larger stimulus correlates with more energy deposited into the system (through lead/contact resistances). However, the memory effect is also strongly correlated with faster relaxation rates *α*, which anticorrelates with the total energy transferred into the system. If the memory effect were because of some relatively straightforward Bean–Livingston barrier or Bean model pinning effect process^46^, this should be insensitive to how the current is turned off, depending only on the total energy imparted. We note, however, that it is therefore hard to rule out the possibility of thermal quenching playing a role. We posit that the acute sensitivity of the memory effect to the turn-off speed implies some dynamic reorganization or annealing of vortex domains, probably because of some interesting intrinsic physical property (or properties) of UTe_2_.

Further arguments against extrinsic heating effects originate from the fact that this phenomenon is only observed in the narrow regime of *B*–*θ* space directly underneath SC2, with no hysteretic *V*(*J*) phenomena observed in purely SC1 or SC2. The memory effect is most pronounced at the lowest temperatures and quickly diminishes on warming (Fig. 3), providing perhaps the strongest argument against any heating-induced artefacts. This is in sharp contrast to the case of NbSe_2_, in which hysteretic *V*(*J*) profiles are enabled close to *T*_c_ (ref. ^23^). Instead, our observations strongly indicate that the hysteretic *V*(*J*) behaviour of UTe_2_ is because of some intrinsic low-energy phenomenon, with an energy scale much lower than *T*_c_.

We propose that the memory effect of UTe_2_ may be the result of competition between two different vortex structures—one native to SC1 and the other to SC2. In the equilibrium state, the vortices arrange themselves in a highly ordered configuration to minimize entropy and energetic costs. On perturbing the system, current densities above *J*_c_ are applied, inducing a flux-flow regime in which vortices are dissipatively travelling through the sample. If the perturbation is sufficiently sharp—for example, if the relaxation rate *α* is very fast—then when *J* drops back below *J*_c_, vortex flow suddenly halts and the vortices are abruptly locked into their new positions. We can imagine a glassy vortex state has now been quenched in the material, which has much higher disorder than the original equilibrium vortex lattice. Higher disorder implies stronger pinning forces between vortices and hence the higher *J*_c_ values that characterize the memory effect. The system can then be reset by smoothly raising *J* back above *J*_c_ before gradually ramping back down to zero current. This could be thought of as annealing the system, thereby returning to the equilibrium low-*J*_c_ state. In the Supplementary Information, we provide phenomenological modelling of the memory effect and show how our experimental observations can be qualitatively understood within this interpretation.

## Outlook

An attractive technological use case for the superconducting memory effect is as an ultralow-power alternative to solid-state or hard disk drive memory functionality. Moreover, the inherent plasticity of the effect presents a promising route towards cryogenic neuromorphic processing. For the bulk millimetre-sized single-crystal specimens investigated here, to probe (read) whether the material is or isn’t in the memory state—which could correspond to a binary 1 or 0—required a power consumption of about 1 nW. Scaling down even just mesoscopically to the order of one square micron in size would reduce this to ≲1 pW—orders of magnitude less than for nanometre-sized silicon elements^47^. Furthermore, whereas cryotron-based^48^ superconducting devices require transitioning to the normal state, UTe_2_ has the distinct advantage that both read and write operations can be performed while superconducting. Recent studies have demonstrated the ability of this material to be carved at the micron-scale by focused ion beams^32,49^, making the prospect of lithographically processing UTe_2_ wafers^50^ a realistic, albeit ambitious, goal.

Further work is required to assess whether similar memory phenomena could be exhibited by other multiphase superconductors such as CeRh_2_As_2_ (ref. ^1^) or UPt_3_ (ref. ^2^). We hope that our discovery here may help catalyse efforts to discover new multiphase superconductors composed of more abundant elements, which might exhibit the memory effect to higher temperatures. Although the list of known multiphase superconductors is short, notable recent additions from materials as diverse as moiré graphene^51^ and high-*T*_c_ nickelates^52^ suggest that further promising candidates await discovery.

In summary, we studied the *J*–*V* characteristics of the multiphase superconductor UTe_2_. In an intermediate phase space regime bordering two superconducting phases, we observed anomalous hysteresis in the value of the critical current, dependent on the history of applied direct current ramps and pulses. We showed that modulating the amplitudes and timescales of dc electrical stimuli can selectively tune between low and high critical current states, with the material then ‘remembering’ which state it is in. We interpret this to be because of different vortex configurations being either abruptly quenched or smoothly annealed. Our work demonstrates that, in principle, the vortex matter of an unconventional superconductor can be electrically manipulated and used to encode and process information, thereby opening up new possibilities for low-energy electronic devices, cryogenic neuromorphic memory and quantum computational hardware.

## Methods

### Sample preparation

Single-crystal UTe_2_ specimens were grown in a flux of molten salts^53^ by the recipe detailed in ref. ^54^. Samples were contacted by spot-welding four gold wires, each of 50 μm in diameter, for respective current and voltage leads. These were subsequently connected to copper twisted pairs, which were soldered to the wiring of the probe. Each of the three samples investigated in this study had *J* sourced along the [100] direction, with leads affixed to the (001) surface. Residual resistivity ratio measurements were performed using a Quantum Design Physical Properties Measurement System in Cambridge with low-frequency ac excitations. The residual resistivity ratio values for the three samples are S1:105, S2:44 and S3:395.

### Electrical transport measurements

**Extended Data Fig. 6:**
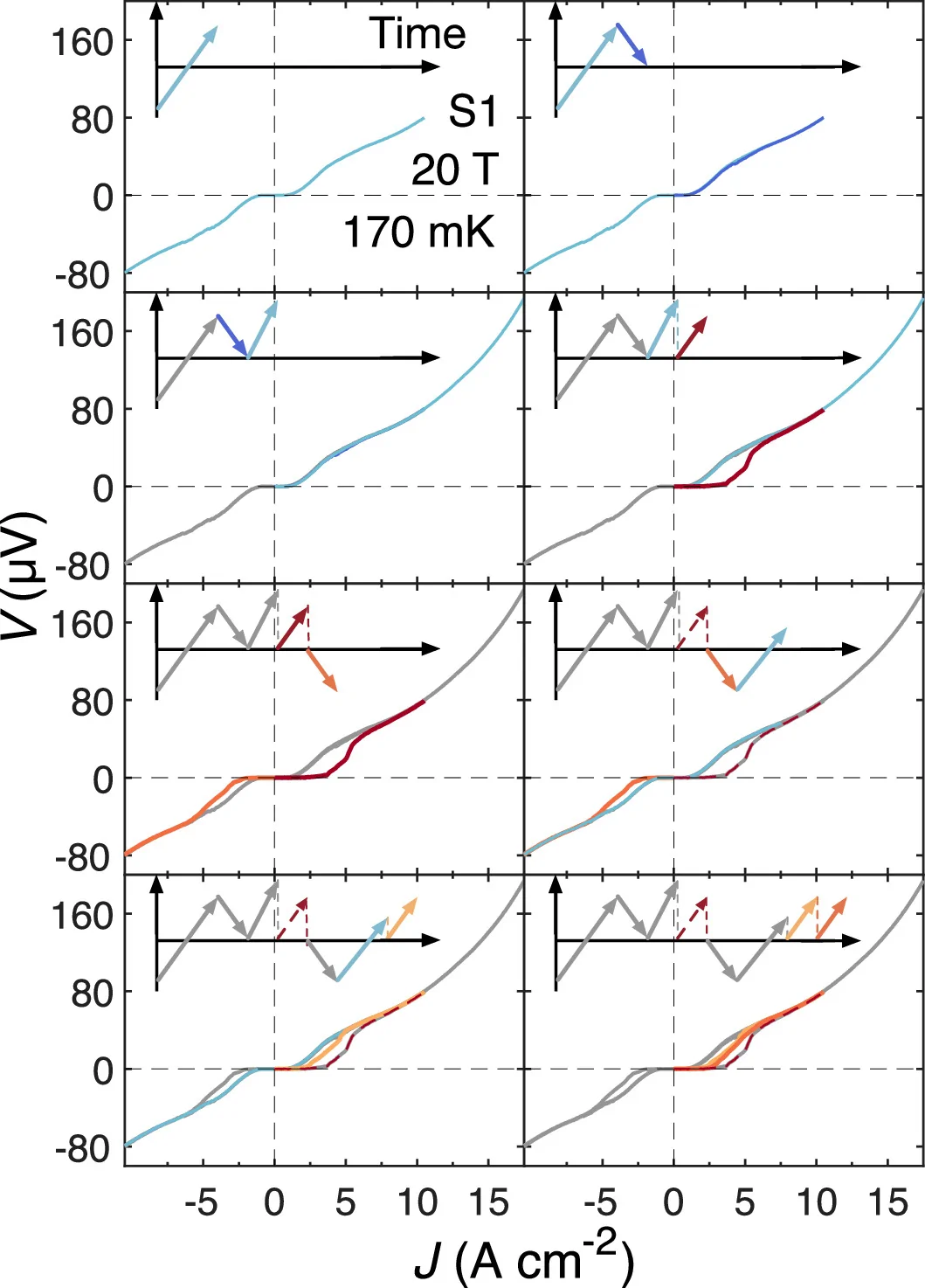
Tuning of memory hysteresis. Controllability of the superconducting memory effect by modulation of the perturbation amplitude. Insets show the chronological sequence of how *J* was swept. In Fig. 1, we always perturbed the system by discontinuously changing *J* from the same magnitude—by contrast, here we perform the perturbation from two different values of *J*. A larger hysteresis in *V*(*J*) is observed for the larger perturbation. This shows that the magnitude of the memory effect is tunable by the size of the excitation, similar to Fig. 2.

**Extended Data Fig. 7:**
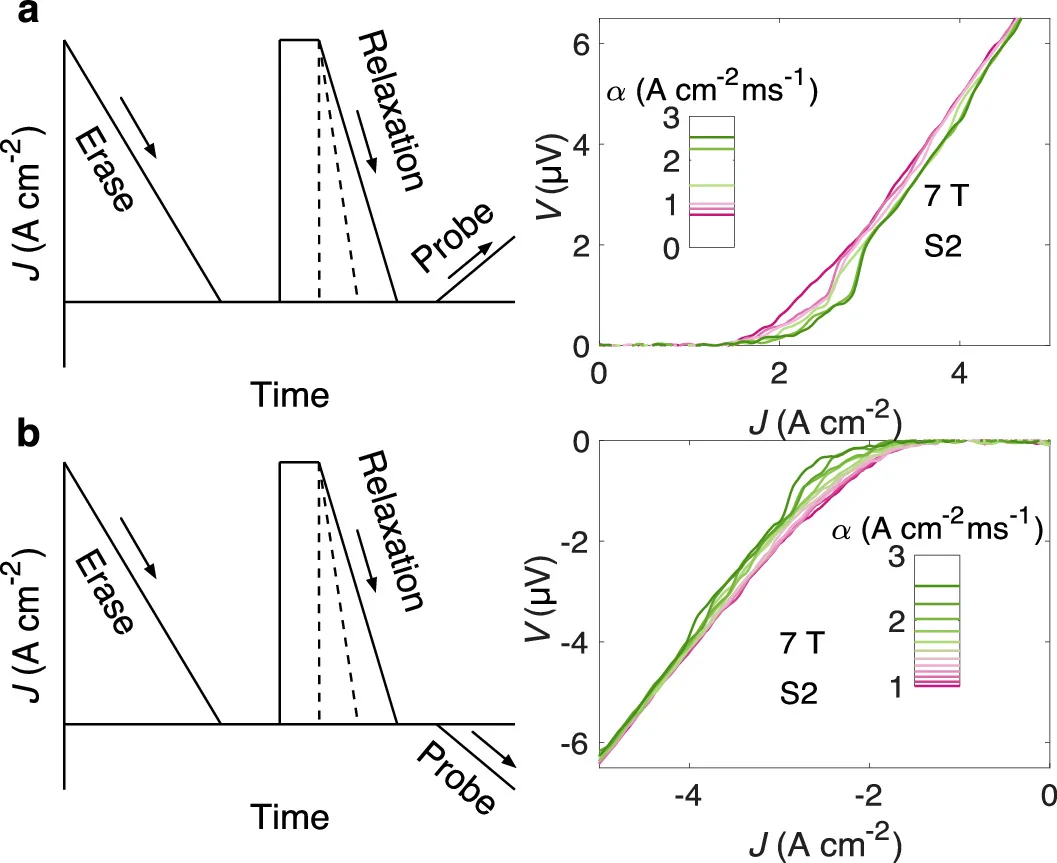
Presence of the memory effect under both polarities of current flow. **a**,**b**, Results for positive (**a**) and negative (**b**) polarity of *J*. The same (positive) polarity of erasure and perturbation were performed in both instances, with the response then measured upon sweeping *J* to large positive and to large negative values (as depicted in the schematics on the left). Consistent behaviour is observed for both polarities.

**Extended Data Fig. 8:**
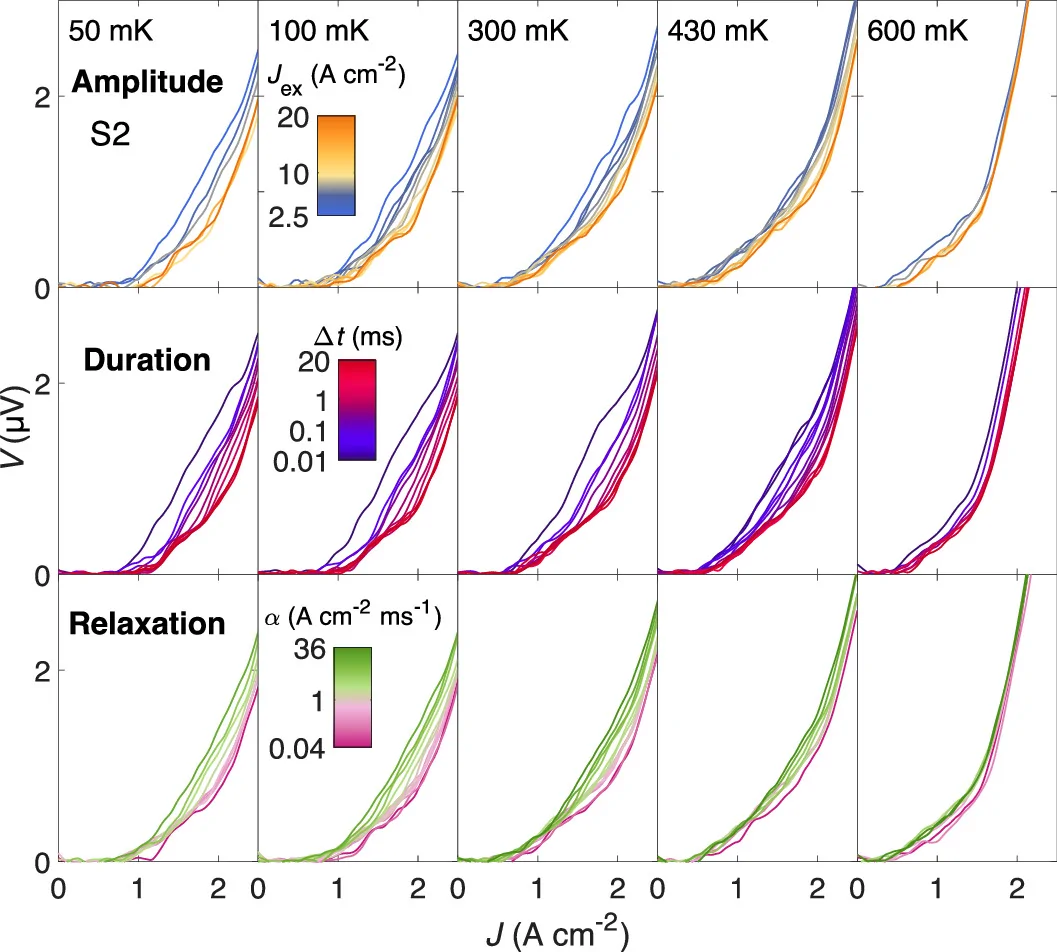
Raw temperature-dependent data. Hysteretic *J*–*V* measurements performed on sample S2 at 14 T at 50 mK, 100 mK, 300 mK, 430 mK and 600 mK for amplitude, duration and relaxation rate tuning. These data were used to generate the heatplots presented in Fig. 3 and Extended Data Fig. 2.

**Extended Data Fig. 9:**
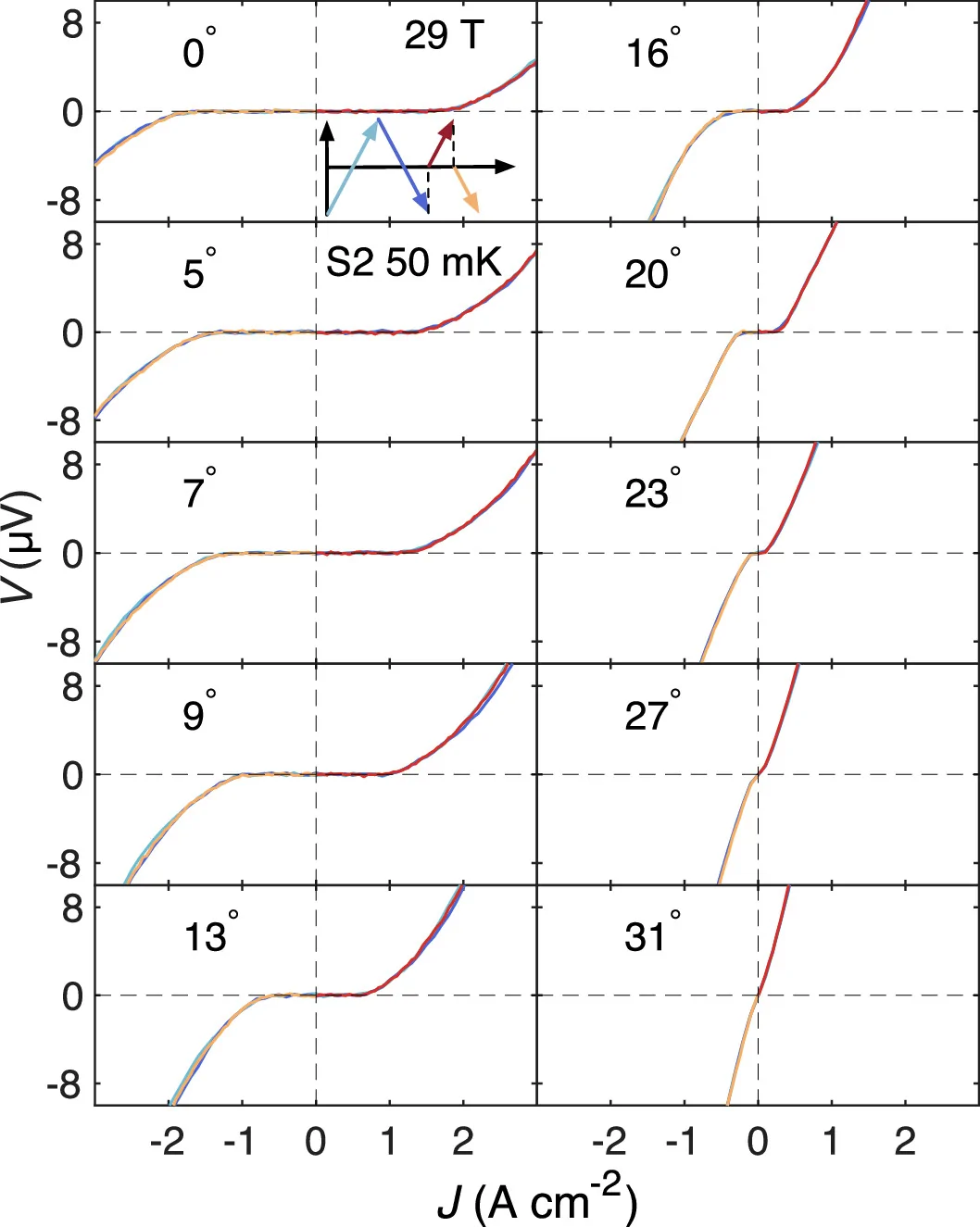
Rotation study showing no memory effect in SC2. *V*(*J*) at *B* = 29 T for rotations of *B* from the $\hat{b}\mathrm{-axis}$ (*θ* = 0°) towards the $\hat{c}\mathrm{-axis}$ (*θ* = 90°). The measurement protocol is specified in the top-left inset. No memory effect is discerned at any *θ*.

The hysteretic *J*–*V* properties of UTe_2_ were investigated by performing direct current measurements using a Keithley 6221/2182A combined current source–multimeter system. The in-built pulse-delta sweep mode and arbitrary waveform generator features were used. Measurements were performed in a 30 T superconducting magnet with Oxford Instruments dilution fridge system at the Synergetic Extreme Condition User Facility (SECUF) in Beijing^55^. All measurements presented in this study were performed in that system, except those in Fig. 4h and Supplementary Fig. 2 that were performed at the National High Magnetic Field Laboratory in Florida, in a resistive magnet using a ^3^He system, in which low-frequency ac measurements were obtained using a Keithley 6221 current source and a Stanford Research 860-series lock-in amplifier. For the electrical switching study at SECUF, a sequence of pulse-delta measurements with different applied currents was defined by entering the starting current value, ending current value and the number of steps for the sweep. To control the amplitude, duration and relaxation rate, a varying shape excitation pulse was defined by 100 points and generated with the arbitrary waveform feature of the source–multimeter system. Control of the instrument was realized by custom-developed Python code with the pymeasure package^56^, which is included as a supplement to this article (Supplementary Information). The reset protocol before performing a perturbative excitation was achieved by sweeping the current from 40 mA to 0 mA smoothly with a 2-mA step over a 20-s time interval with the pulse-delta sweep mode. The cycling sequence in Fig. 1d was performed by the pulse-delta mode. The time sequence is defined by entering the current amplitude and number of steps. Further electrical transport measurements are presented in Extended Data Figs. 6, 7, 8 and 9.

## Online content

Any methods, additional references, Nature Portfolio reporting summaries, source data, extended data, supplementary information, acknowledgements, peer review information; details of author contributions and competing interests; and statements of data and code availability are available at https://doi.org/10.1038/s41586-026-11015-3.

## Supplementary information

Supplementary Information Supplementary Figs. 1–8, Supplementary Sections 1 and 2 and Supplementary References

Peer Review File

## Source data

Source Data Fig. 1

Source Data Fig. 2

Source Data Fig. 3

Source Data Fig. 4

## Data Availability

The datasets supporting the findings of this study are available from the University of Cambridge Apollo Repository^57^. Source data are provided with this paper.
