# Supplementary material for: Electrically controllable superconducting memory effect in UTe2
Source: Nature. 2026 Sep 16;657(8132):632–7. doi: 10.1038/s41586-026-11015-3 (PMC13581588; doi:10.1038/s41586-026-11015-3)
Supplement: Supplementary file 1 [file 41586_2026_11015_MOESM1_ESM.pdf]

nature portfolio
https://doi.org/10.1038/s41586-026-11015-3

Supplementary information

Electrically controllable superconducting memory effect in UTe_2

In the format provided by the
authors and unedited

Supplementary information for:

Electrically-controllable superconducting memory effect in UTe_2

Zheyu Wu, Hanyi Chen, Mengmeng Long, Daniel Shaffer, Dmitry V. Chichinadze,
Andrej Cabala, Theodore I. Weinberger, Alexander J. Hickey, Dave Graf, Vladimir Sechovský,
Michal Vališka, Gang Li, Rui Zhou, F. Malte Grosche, Alexander G. Eaton
Correspondence to: alex.eaton@phy.cam.ac.uk

This supplement comprises:

Supplementary Figures

Fig. S1. Magnetic field evolution of critical current density J_c.

Fig. S2. Anomalous flux-flow resistive features.

Fig. S3. Investigation of the memory effect in sample S3.

Fig. S4. No discernible heating response during direct current sweeps.

Fig. S5. Temperature variation during memory effect J–V measurements.

Fig. S6. Large change in mixing chamber temperature due to mechanical rotation mechanism.

Fig. S7. Change in curvature of the equilibrium $V(J)$ curves between 50 mK and 1 K.

Fig. S8. Theoretical modelling of the memory effect.

Supplementary Sections

1. Modelling of the memory effect

2. Python code developed for controlling Keithley pulse delta measurement

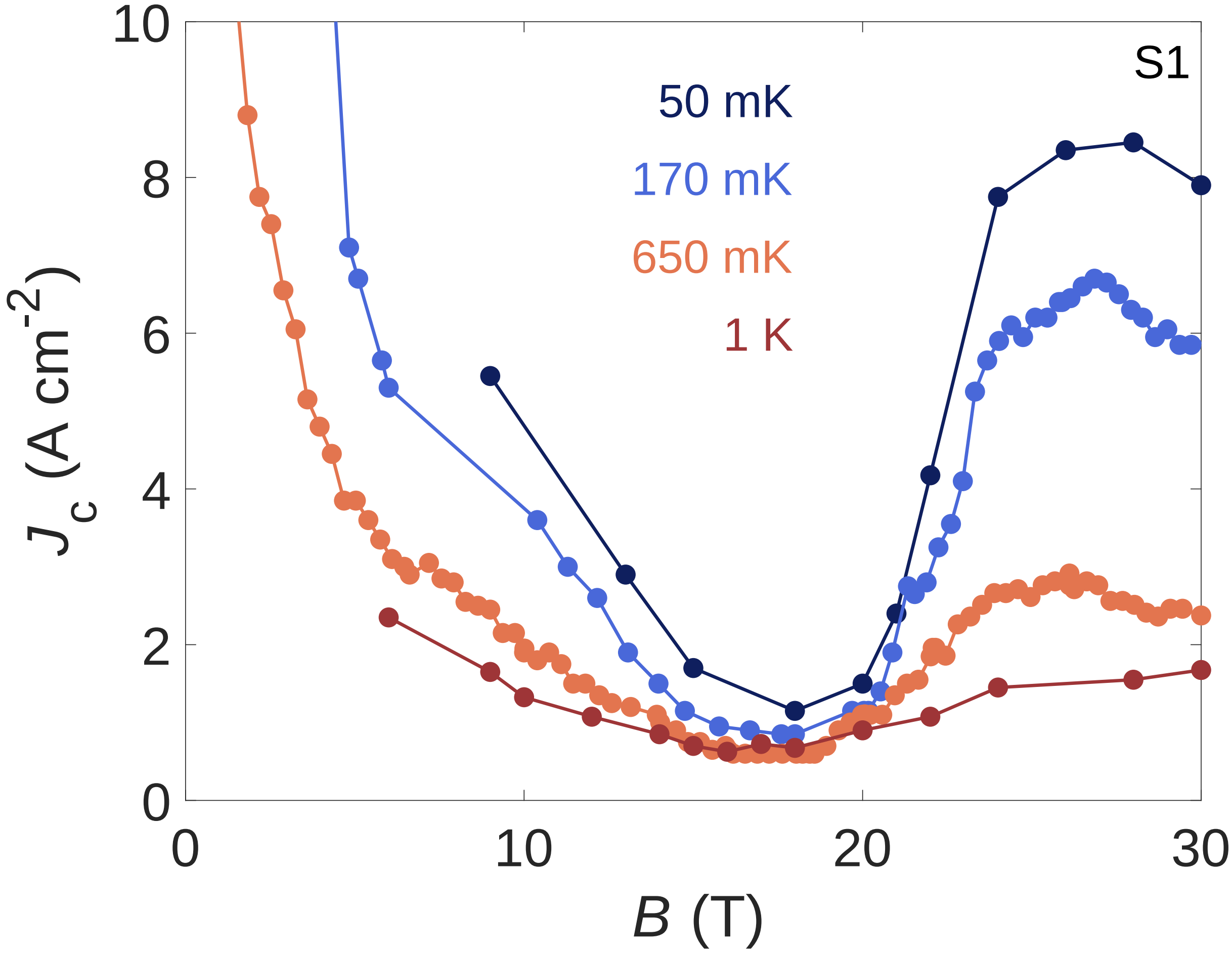

Fig. S1. Magnetic field evolution of critical current density J_c. Here we plot $J_c(B)$ for direct current measurements at incremental temperatures, with field oriented along the [010] direction. All data were recorded on sample S1. An anomalous region of low J_c is observed as UTe_2 transitions between the SC1 and SC2 states.

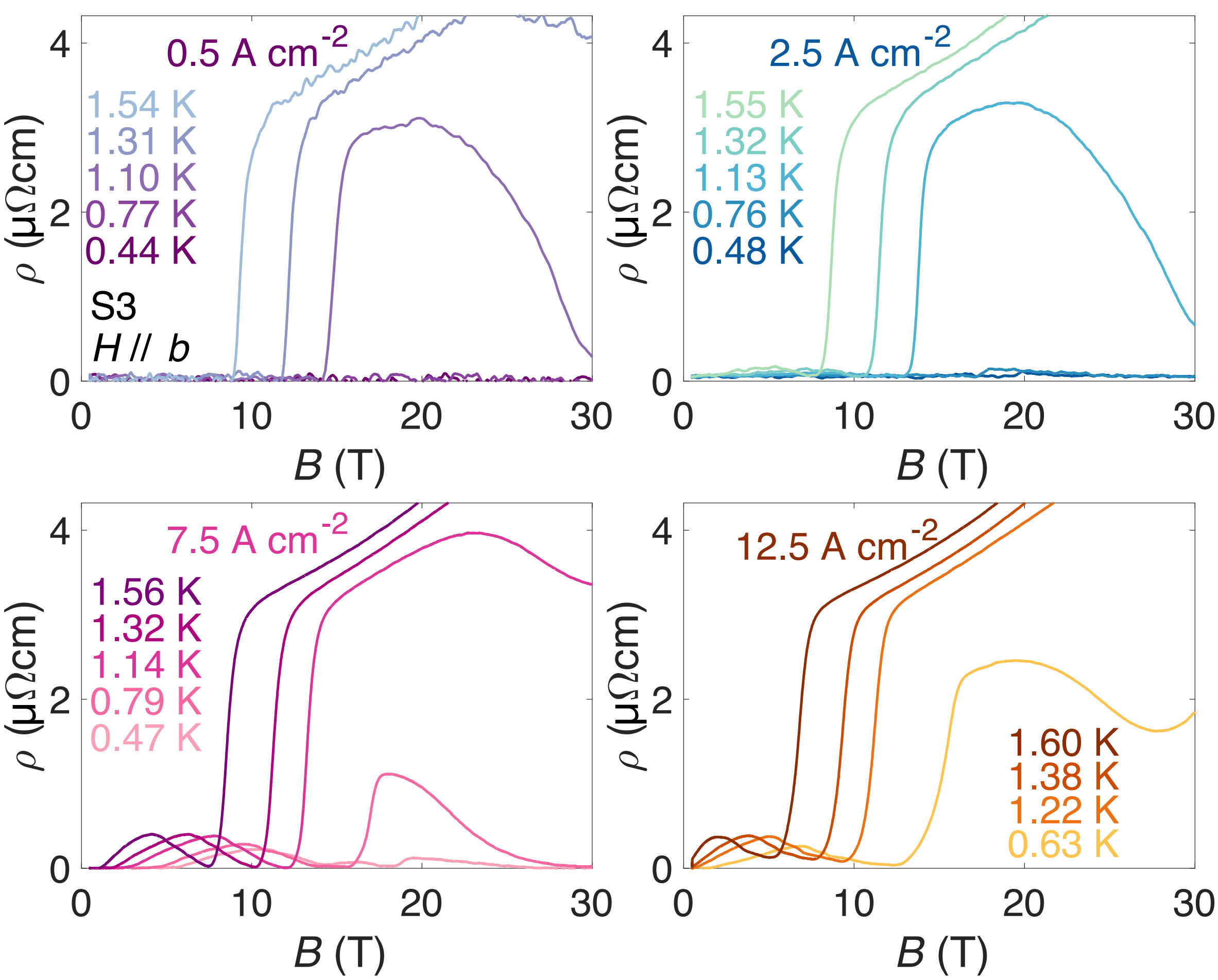

Fig. S2. Anomalous flux-flow resistive features. Magnetic field sweeps of the effective resistivity ρ for incremental J and T values as indicated, with field along [010]. The data in this figure were acquired by low frequency ac measurements. For the lowest current density of 0.5 A cm^{-2}, $\rho = 0$ at low T for all B. Upon warming, the resistive transition between SC1 and the normal state is found above 10 T at 1.10 K. Contrast this with the case of J = 6.5 A cm^{-2}, where anomalous flux-flow behaviour is observed at low fields and temperatures. This points to unusual vortex dynamics, likely with complex vortex-vortex interactions. Note that these data were recorded on sample S3, which has the highest residual resistivity ratio (RRR = 395) of the samples investigated in this study. That indicates a low density of pinning sites, implying that vortex flow should be more easily effected than in lower RRR samples, where vortices would be expected to be more easily pinned to defect sites.

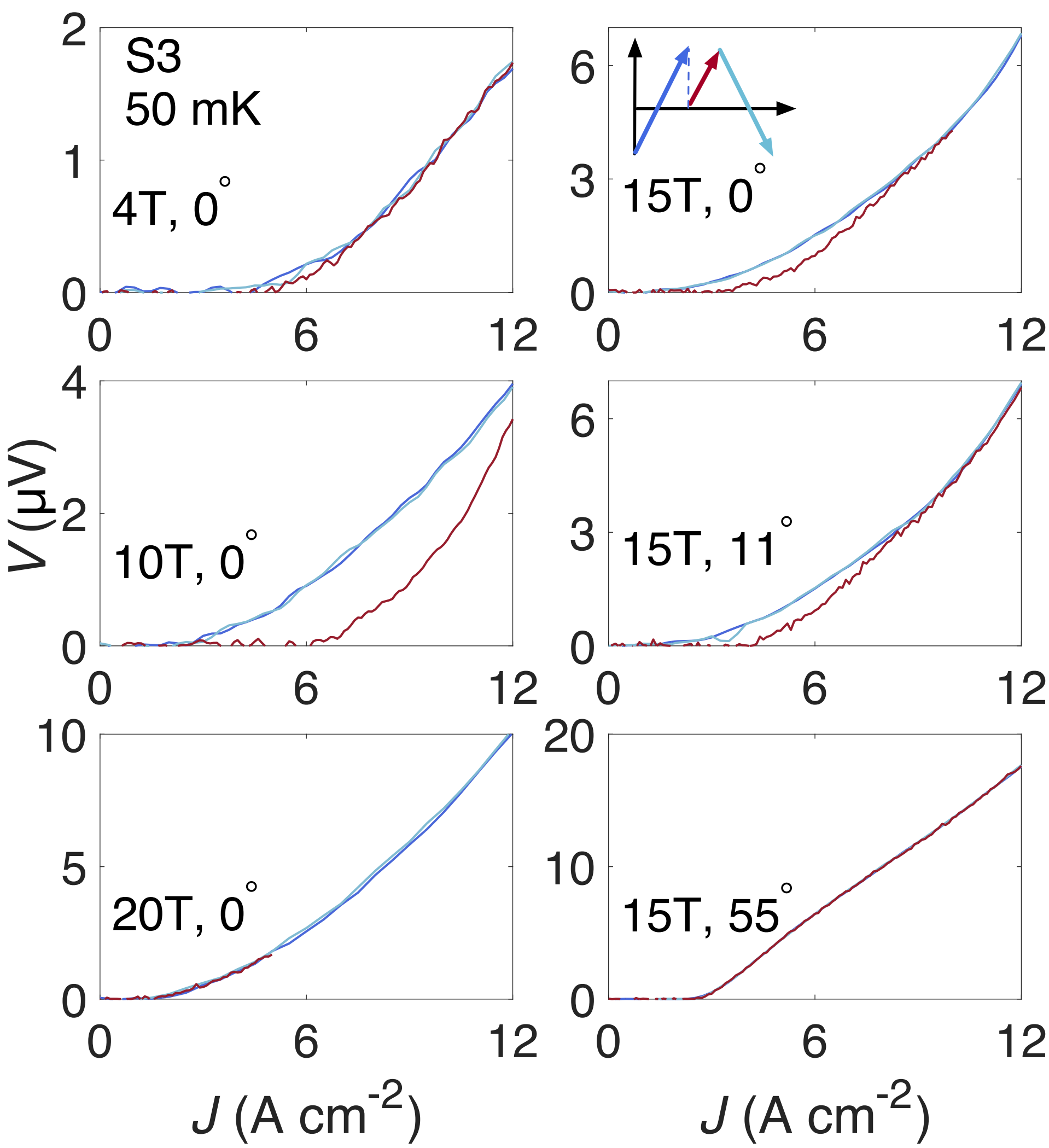

Fig. S3. Investigation of the memory effect in sample S3. Here we perform a similar sequence of memory-mapping measurements for sample S3 as was done for sample S2 in Figure 4 of the main text. There is approximately a factor of 8 difference in the residual resistivities of these two samples, which indicates quite different levels of crystalline disorder. Both samples show a prominent memory effect around 10 T for T = 50 mK (see main Fig. 4e). For the case of S2, the hysteresis in $J(V)$ is maximal at 10 T, but is still well resolved at 15 T. By contrast, here S3 exhibits a markedly smaller hysteresis loop at 15 T. This points towards an important role for vortex pinning in manifesting the memory effect.

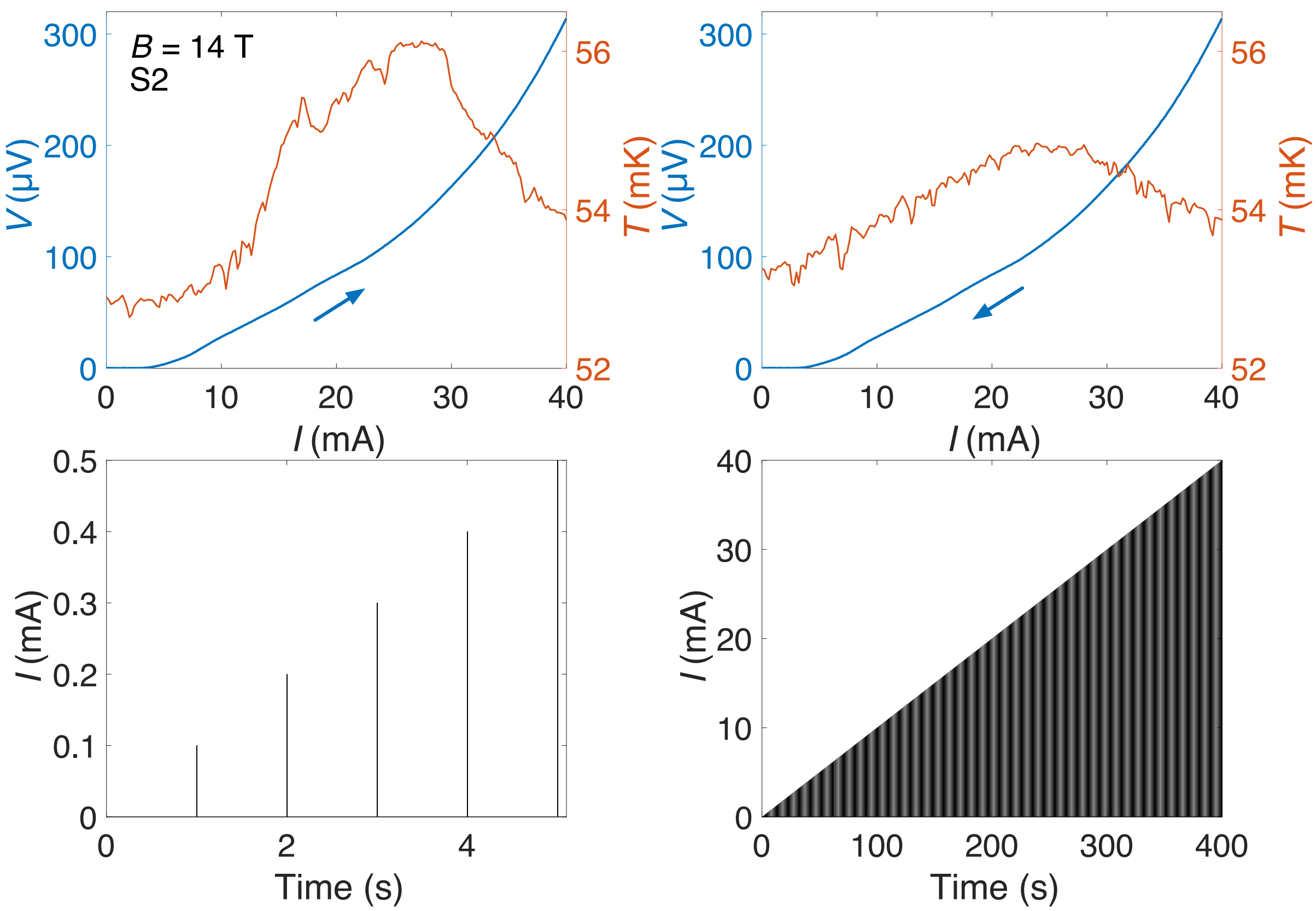

Fig. S4. No discernible heating response during direct current sweeps. Here we plot simultaneous measurements of voltage and temperature during rising (top left) and falling (top right) current sweeps. A ruthenium oxide thermometer was utilised, which was positioned within 5mm of the sample and secured to the sample platform by General Electric low-temperature varnish. The Keithley delta-pulse protocol was utilised (see *Methods*). Some millikelvin-level of temperature drift is recorded, which does not appear to correlate with the applied current. The lower panels show how the current was modulated as a function of time. This sequential current ramp would correspond to e.g. the upwards red-coloured trace in Fig. 1b of the main text.

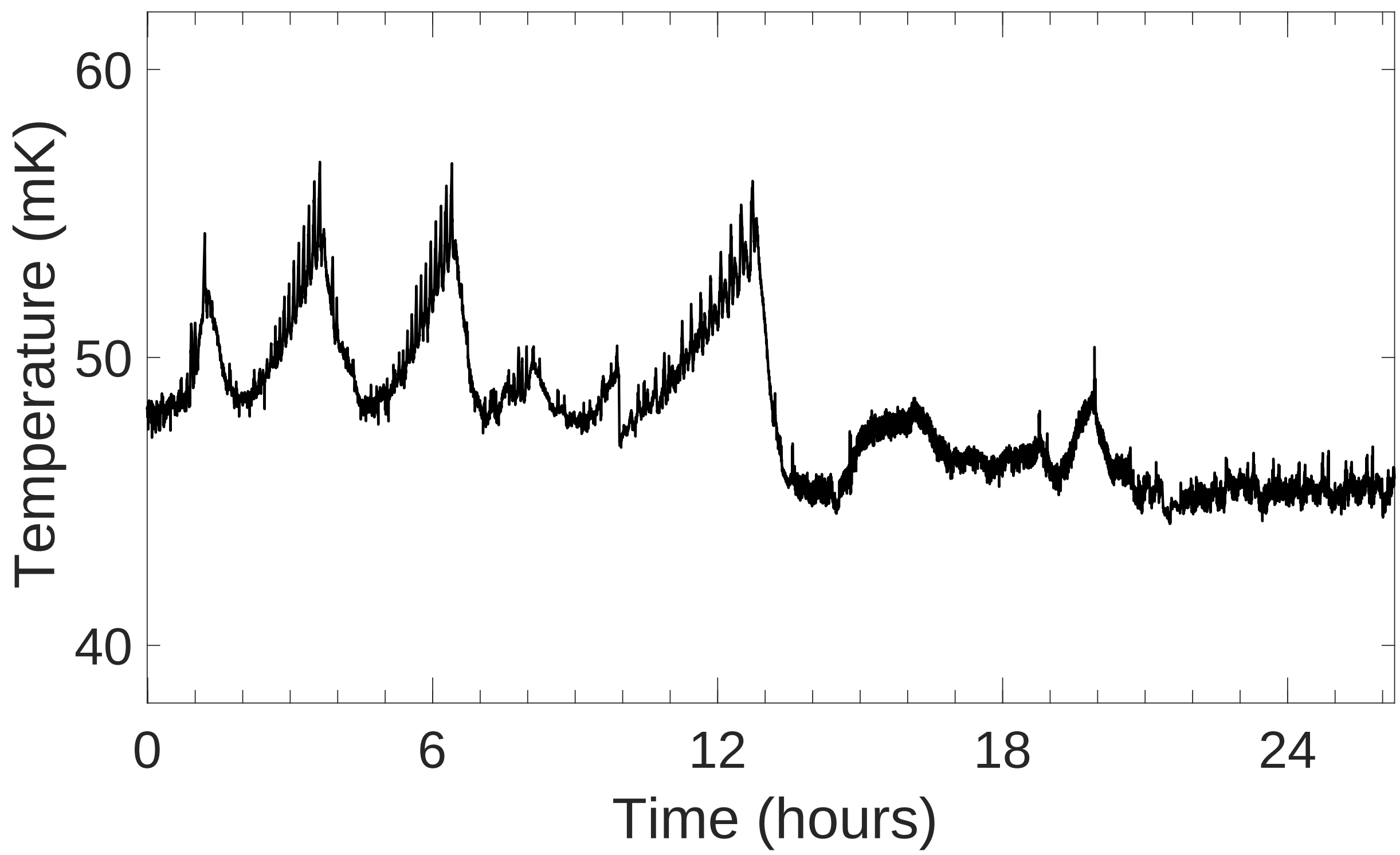

Fig. S5. Temperature variation during memory effect $J-V$ measurements. Here we plot the thermometry readings during a day of measurements in the 30 T all-superconducting magnet at SECUF, Beijing, China. The base temperature of this system during this run of measurements was approximately 50 mK. Small spikes are visible where we performed pump/erase current sweeping protocols to map the memory effect of UTe_2. However, the scale of these is very small. Measurements were performed in a dilution fridge sample environment. The vast majority of Joule heating occurred within the wires running down the probe, which was dumped into the 1 K pot. This temperature versus time recording shows that very little heating occurred within the mixing chamber of the fridge, as detected by the thermometry.

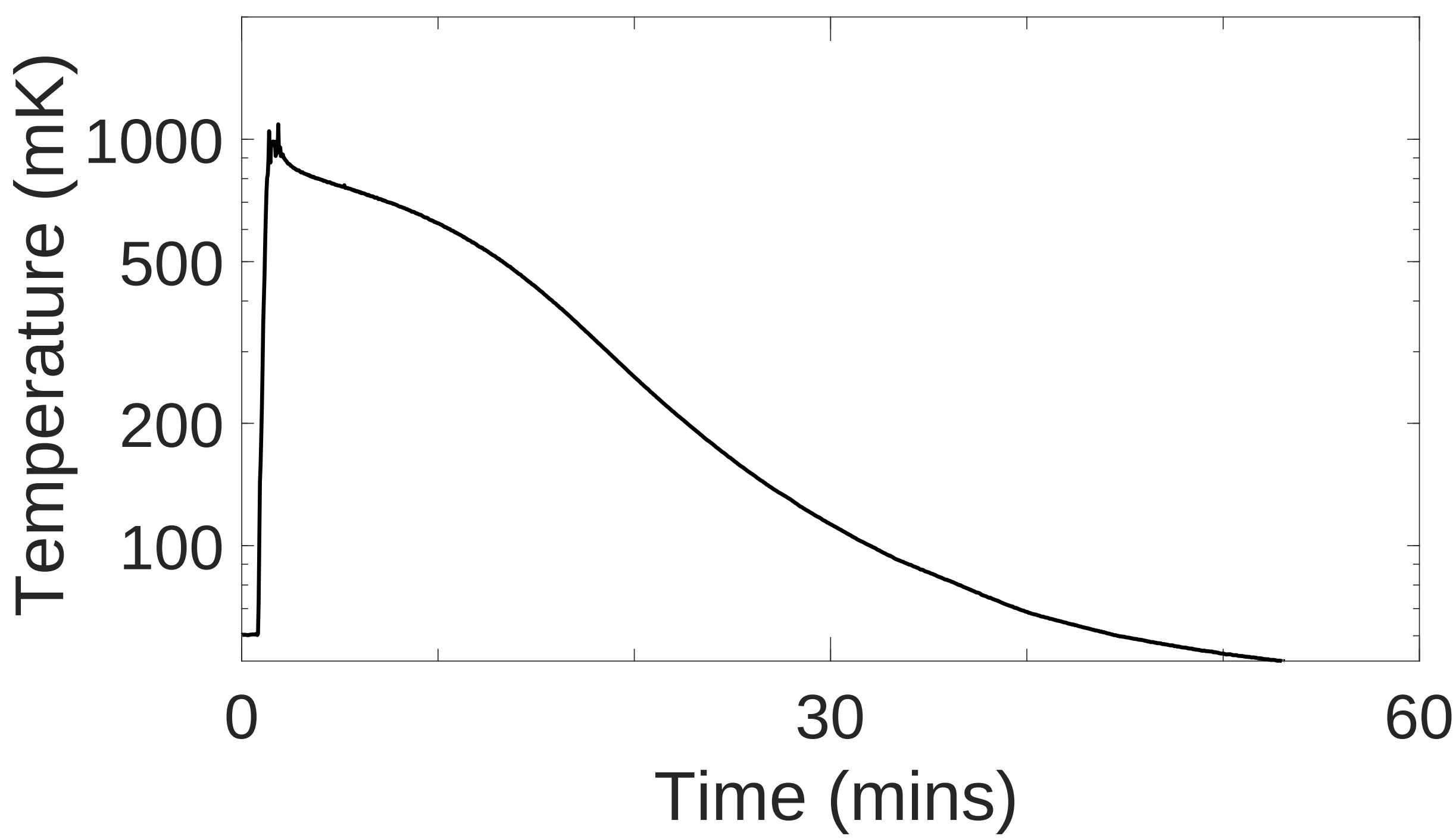

Fig. S6. Large change in mixing chamber temperature due to mechanical rotation mechanism. In contrast to the negligible temperature changes caused by our millisecond-scale current probe protocols, the friction-based rotation mechanism caused considerable heating every time we incremented the angle. Therefore, every time we changed angle we would then wait approximately an hour for the system to cool back down before taking further measurements at base temperature.

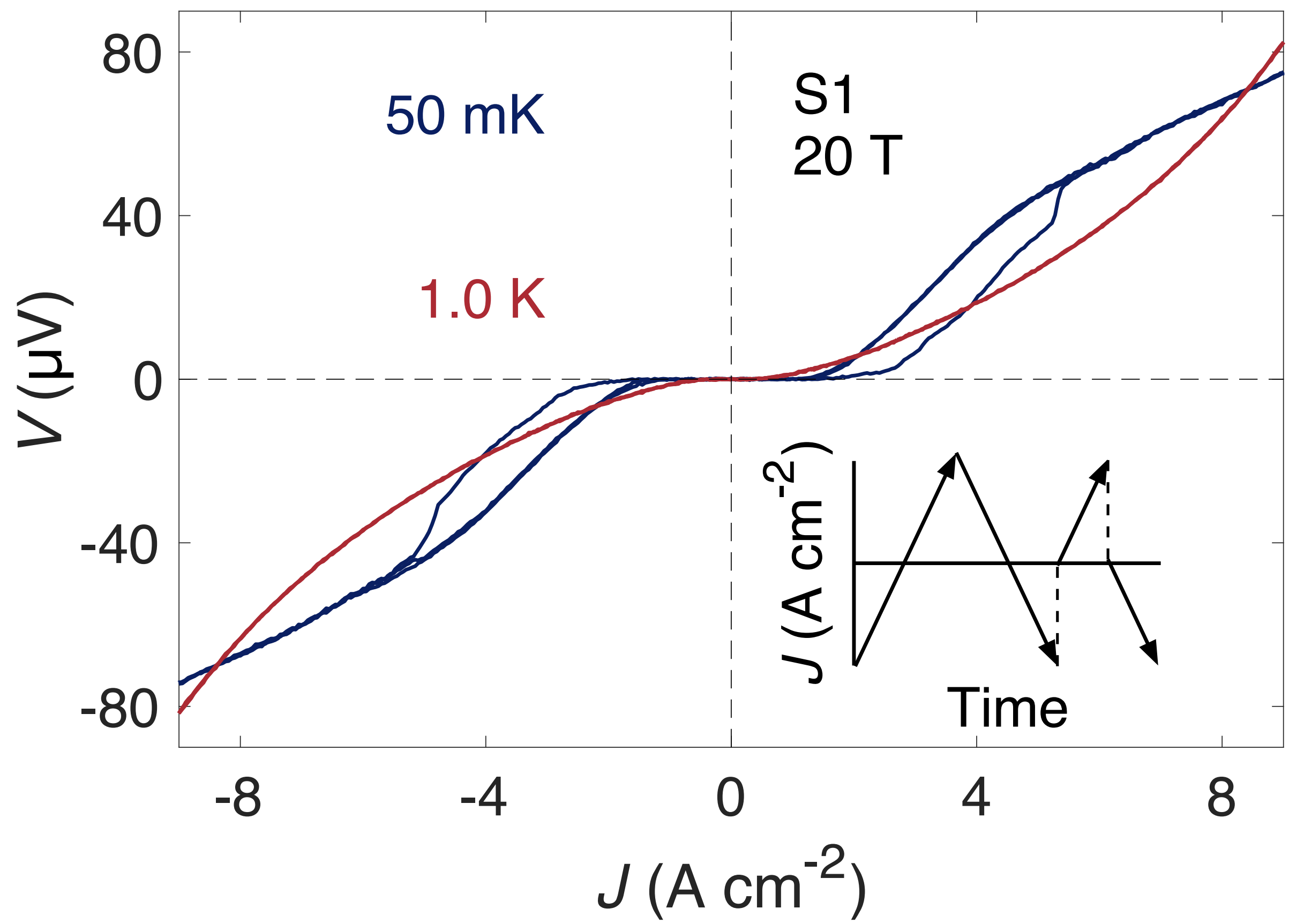

Fig. S7. Change in curvature of the equilibrium $V(J)$ curves between 50 mK and 1 K. Here we replot data from main Fig. 3, on the same axes. The memory effect is clearly discerned at 50 mK, which is absent at 1.0 K. Interestingly, the curvature of $V(J)$ is markedly different in the flux-flow regime for these two temperatures, pointing to a significant change in the properties of the underlying vortex liquid.

1 Modelling of the memory effect

In further consideration of the memory effect of UTe_2, here we present a phenomenological resistively-shunted-junction (RSJ) model, which we show can qualitatively capture some aspects of the observed hysteretic $V(J)$ phenomenology. Such RSJ modelling is often used to describe transport[1,2] and tunnelling[3,4] in Josephson junctions, but also helps to capture current dynamics features in junction-free samples (including in the presence of vortices).[5,6] However, the same set of mathematical equations describes the motion of an individual vortex (or the rigid motion of a vortex lattice) in a periodic potential.[6] We will for now leave open the question of the precise interpretation of the model and only speculate about their possible origins at the end of this section.

The RSJ model assumes that the current has three components: the supercurrent $J_S(\phi)$ that is a function of ϕ (which depending on the physical interpretation may be the superconducting phase across the sample or the vortex position; see below), the normal current $J_N = \gamma V$ with damping $\gamma \sim 1/R$ (proportional to the inverse of the effective resistance), and the noise current J_n which we find accounts for vortex flow characteristics of the experimental J-V curves. In general, $J_S(\phi) = U'(\phi)$, where $U(\phi)$ is the energy of the superconductor and the prime denotes differentiation with respect to ϕ, and the Josephson relation implies that $\dot{\phi} = 2eV/\hbar$. Finally, the noise current J_n satisfies $\langle J_n(t)J_n(t')\rangle \sim \delta(t-t')$ (uncorrelated in time t). We neglected capacitive effects as no hysteresis is seen in the absence of a pump (corresponding to an overdamped Josephson junction limit in the RCSJ model). Note that in general the damping coefficient $\gamma(\phi)$ may depend on the parameter ϕ.

Physically, such an RSJ model may be derived from a microscopic model of a superconductor using a time-dependent Ginzburg-Landau theory,[1,2,5,6] but in presence of vortex dynamics a rigorous microscopic derivation is prohibitively costly. We do not attempt it here. Mathemat-

ically, on the other hand, the RSJ equations can describe multiple analogous physical systems, for example a pendulum subject to a torque $\tau \sim J$, with ϕ corresponding to the deflection angle of the pendulum, $U(\phi)$ to the gravitational potential, γ to the damping, and $\dot{\phi} \propto V$ to the angular velocity of the pendulum. The nonzero voltage state then corresponds to a steady state in which the pendulum completes full revolutions. Similarly, this treatment is also analogous to the description of a particle with position variable ϕ in a tilted washboard potential $U(\phi) - J\phi$. In the context of vortex (or fluxon) motion in a bulk superconductor, ϕ in particular has the literal interpretation as the position of the vortex, and $U(\phi) - J\phi$ is thus the effective potential felt by the vortex (we then assume $U(\phi)$ to be periodic for simplicity).[6]

Putting aside the physical interpretation for the moment, we simply determine the requirements for $U(\phi)$ and $\gamma(\phi)$ in order to qualitatively match the experimental data. The first requirement is that the energy landscape $U(\phi)$ must include both a global and at least one additional local minimum with respect to ϕ. The local minimum corresponds to the metastable state in the SC1.5 region of the UTe_2 phase diagram. The local minimum should respect inversion symmetry since the $J - V$ curves seen in experiment are symmetric under inversion which reverses both J and $V \propto \dot{\phi}$ (and thus also reverses ϕ). This implies that the local minimum must occur at $\phi = \pi$, since the global minimum occurs at $\phi = 0$. We took a simple form of the potential with this feature:

$$U(\phi) = (2 - \cos\phi - \cos 2\phi)\left(1 - 0.6\left(\frac{1-\cos\phi}{2}\right)^{\beta}\right), \tag{1}$$

plotted in Fig. S8 a. A similar potential has been considered in ref.,[1] which studied a Josephson junction with a potential with two minima. The β parameter determines the critical current of the local minimum at $\phi = \pi$. In our calculations we chose $\beta = 40$, such that the minimum at $\phi = \pi$ has a higher critical current than the global minimum at $\phi = 0$.

A second requirement we find is that the damping parameter must vary between the two minima. Qualitatively, this can be understood from the tilted washboard potential analogy:

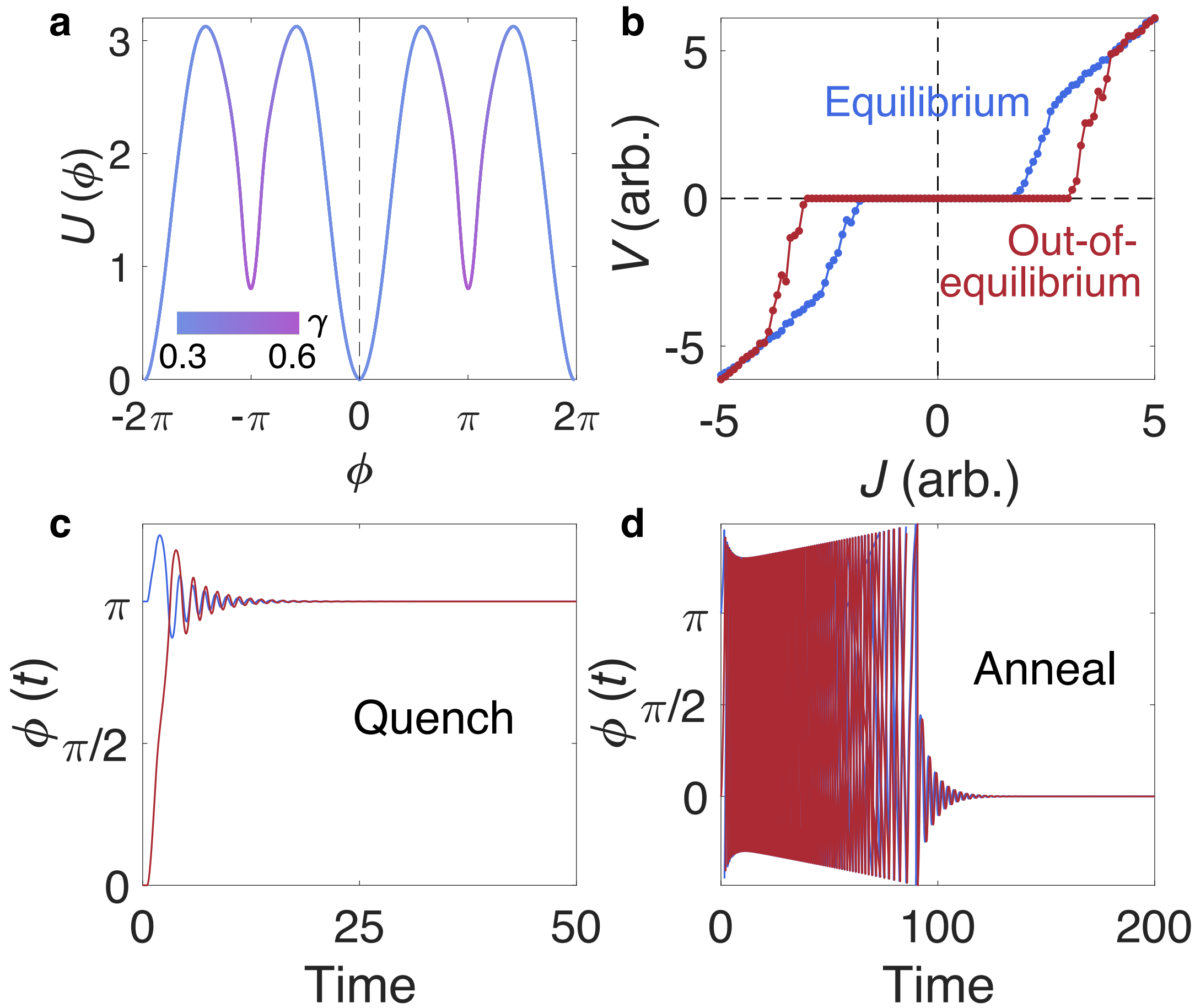

Fig. S8. Theoretical modelling of the memory effect. a, Plot of the potential $U(\phi)$ from Eqn. 1. Note the two different minima: global at $\phi = 0$ and the local "glassy" minimum at $\phi = \pm\pi$. The colour code illustrates the value of the parameter γ characterising "friction". **b,** The resulting $J - V$ characteristic for the case with random noise and averaged over $N = 60$ noise realizations. **c,** Time evolution of $\phi(t)$ for the short current pulse ("quench") and **d,** same as in the previous panel but for the erasing current ("annealing").

under a current pulse, the potential $U(\phi) - J\phi$ is tilted and then quickly relaxed as the current is turned off. The fictitious particle then starts rolling along the ϕ axis and then relaxes due to the friction γ. If the friction at the local minimum is much higher than at the global one, the particle tends to relax to the local minimum instead of the global minimum during a fast quench. On the

other hand, during a slow current relaxation, the particle has enough time to relax at the global minimum.

We confirm this qualitative expectation in quantitative simulations, taking the damping to be

$$\gamma(\phi) = \gamma_0 + \gamma_\pi \left(\frac{1 - \cos\phi}{2} \right)^{\delta} \tag{2}$$

with parameters γ_0 and γ_π chosen to be 1, and the δ parameter (chosen to be 20 in the simulation) determines the degree of localisation in the increase of the damping; see the colour coding in Fig. S8 a. The memory effect is a dynamical effect involving the time evolution of $\phi(t)$. This time dependence is determined by the equation

$$J = J_S + J_N + J_n = U'(\phi) + \gamma(\phi)\dot{\phi} + J_n \tag{3}$$

To study the dynamics of the junction we numerically solve Eq. (3) with initial conditions $\phi(0) = 0, \pi$ for different current driving regimes. The two types of driving we consider are: (i) a short rectangular current pulse (quench; out-of-equilibrium regime) and (ii) a slowly varying triangular current pulse ("annealing" or "erasure"; quasi-adiabatic evolution), mimicking the experimental setup. Within our effective description the memory effect is achieved by the switching between different minima of the effective potential: for the regime (i) starting from either of the two minima, the system ends up in the minimum corresponding to $\phi = \pi$. Conversely, for the erasure regime (ii), the system ends up in the $\phi = 0$ minimum regardless of the initial ϕ. Such switching can be seen in plots of the explicit time dependence of the phase $\phi(t)$ in Fig. S8 c,d. We interpret this non-equilibrium switching due to the quench to be associated with the formation of a long-lived transient (metastable) state.[7] To plot the $J - V$ curves we find the asymptotic behaviour of $\frac{d\phi(t)}{dt} \propto V$ on large timescales, so that the system reaches a (meta)stable state, see Fig. S8. The added noise is averaged over $N = 60$ realizations of the $J - V$ curves.

Having established the requirements for the phenomenological parameters $U(\phi)$ and $\gamma(\phi)$, we now speculate on their possible origin and the interpretation of the metastable state within this model. First we observe that the memory effect is only seen in a narrow region of $B-\theta$ space (Fig. 4 of the Main Text) where SC1 and SC2 coexist, suggesting that the presence of multiple order parameters is crucial for realising a non-trivial local minimum of $U(\phi)$. This could be either at the level of the microscopic order parameter (Δ_1, Δ_2), or due to the presence of multiple mesoscopic domains with distinct SC states.

In order to further interpret the parameters, we next need to consider the meaning of the parameter ϕ. In the presence of vortices and possible domain walls, this is not straightforward. To simplify, we can first consider the case of a single vortex, or a rigid vortex lattice, with coordinate ϕ moving through a periodic effective vortex pinning potential $U(\phi)-J\phi$.[6] Crucially, the current enters the effective potential due to the Lorentz force, and the Josephson relation $\dot{\phi} = 2eV/\hbar$ still holds due to Faraday's law (since magnetic flux moves with the vortex lines). The two minima of U, in that interpretation, correspond to two distinct vortex positions X_0 and X_π within a unit cell of the vortex lattice, and slowly decreasing a current may correspond to an annealing process in which the vortices relax into more stable (equilibrium) positions.[8]

In a more realistic description, the vortex positions are not necessarily periodic (glassy vortex states are possible) and vortex interactions are obviously important in forming the vortex lattice and the associated potential. $U(\phi)$ should thus be considered as an effective mean-field approximation, but as in other mean-field problems we expect similar equations to hold qualitatively under reasonable assumptions. Within this picture, we can further speculate on both the origin of the second minimum and the difference in vortex-motion damping at the two minima. In light of the presence of coexisting order parameters, a straightforward interpretation is that the two vortex positions occur within two different domains corresponding to the two order parameters, with some inhomogeneous distribution of such domains. The damping parameter and

the critical currents (determined by vortex viscosity and pinning, respectively) would naturally be different in different domains, determined by the bulk state properties of the corresponding domain.

On the other hand, the domain walls themselves are likely sources of both vortex pinning and drag forces, and may rearrange under current application. The two minima could in principle correspond instead to configurations with a different number of domains, with larger damping consistent with a higher concentration of domain walls in the memory state. Note that larger damping corresponds to a lower resistance $R \sim 1/\gamma$ in the vortex flow state.[8–10] Given that vortex pinning would also be expected to increase with the number of domain boundaries, this could also account for the higher value of J_c in the memory state.

In the context of the RSJ model of a Josephson junction, ϕ also has the meaning of the superconducting phase difference across the junction. When derived from the TDGL model in the absence of vortices, it is related to the order parameter via $\Delta(r) \propto e^{i\phi r/L}$ where L is the length of the effective unit cell of that state; within that interpretation, the metastable state at $\phi = \pi$ would correspond to a finite-momentum paired state ($\Delta(r) \sim \cos qr$ with $q = \pi/L$), which coincidentally has been proposed to explain other experimental observations of UTe_2, including in the same coexistence region of the phase diagram.[11–13] We note that a state with $\phi \neq 0, \pi$ would result in a superconducting diode effect of the metastable state (i.e., unequal critical currents in that state),[14] which is not consistent with the data we report here.

The two interpretations of ϕ (as a superconducting phase or the vortex coordinate) are, not coincidentally, not mutually exclusive. Since the RSJ model is phenomenological, we can only state that translational symmetry breaking – either by the order parameter itself, due to the presence of domains, or some more complicated vortex physics – is necessary to explain the memory effect. Our data thus does not allow us to determine which of the above mechanisms, if any, are relevant to the experiment. Future imaging experiments may shed further light this.

2 Python code developed for controlling Keithley pulse delta measurement

```python
from datetime import datetime
from pathlib import Path
from time import sleep

import matplotlib as plt
import numpy as np
import pandas as pd
from pymeasure.instruments.keithley import Keithley6221


def unique_filename(directory: str | None, folder_name: str, file_start: str) -> str:
    """Generate a unique filename in the specified directory by appending a counter if
    necessary.

    Args:
        folder_name (str): The name of the folder to create/use within the directory.
        file_start (str): The base name of the file without extension.
        directory (str | None): The directory where the file will be
            saved.

    Returns:
        str: A unique filename with path.
    """
    if directory is None:
        directory = "."
    dir_path = Path(directory) / folder_name
    dir_path.mkdir(parents=True, exist_ok=True)
    counter = 1
    while True:
        filename = dir_path / f"{file_start}{counter:03d}.csv"
        if not filename.exists():
            return str(filename)
        counter += 1


def PulseDelta_IV(
```

```
    GPIB_add: str,
    start_I: float,
    end_I: float,
    step_I: float,
    delay: float,
    compliance: float,
    PWidth: float,
    SDelay: float,
) -> tuple[datetime, int]:
    """This function starts the Pulse Delta Sweep measurement with
    preset current sequences defined by start, end and step.

    Args:
        GPIB_add (str): GPIB address of the source meter
        start_I: starting value of current sweep, [-105 mA, 105 mA], in A
        end_I: ending value of current sweep, in A
        step_I: current step, in A
        delay: how long each point take, in s
        compliance: value in V, I*R_total < V_compliance
        PWidth: pulse width, in s
        SDelay: delay between pulses, in s

    Returns:
        start_time (datetime): time when the measurement starts
        NI (int): number of points in the sweep
    """
    NI = int(np.round(abs((start_I - end_I) / step_I)) + 1)

    instrument = Keithley6221(GPIB_add)

    instrument.source_compliance = compliance

    delay_cmd = "SOUR:DEL " + str(delay)

    instrument.write(delay_cmd)
    instrument.write("SOUR:SWE:CAB OFF")  # compliance abort

    PWd_cmd = "SOUR:PDEL:WIDT " + str(PWidth)
    instrument.write(PWd_cmd)

    SDel_cmd = "SOUR:PDEL:SDEL " + str(SDelay)
    instrument.write(SDel_cmd)

```

```
    count_cmd = "SOUR:PDEL:COUN " + str(NI)
    instrument.write(count_cmd)
    instrument.write("SOUR:PDEL:RANG BEST")
    instrument.write("SOUR:PDEL:INT 10")
    instrument.write("SOUR:PDEL:SWE ON")  # turn on pulse delta sweep
    instrument.write("SOUR:PDEL:LME 2")
    start_time = datetime.now()

    instrument.write("SOUR:SWE:SPAC LIN")
    str_cmd = "SOUR:CURR:STAR " + str(start_I)
    instrument.write(str_cmd)
    stp_cmd = "SOUR:CURR:STOP " + str(end_I)
    instrument.write(stp_cmd)

    step_cmd = "SOUR:CURR:STEP " + str(step_I)
    instrument.write(step_cmd)

    track_cmd = "TRAC:POIN " + str(NI)  # number of points allowed in buffer
    instrument.write(track_cmd)
    instrument.write("SOUR:PDEL:ARM")  # arm wave
    instrument.write("INIT")  # trigger
    return start_time, NI


def PulseDelta_abort_read(
    GPIB_add: str,
    instrument: Keithley6221,
    start_I: float,
    end_I: float,
    step_I: float,
    _delay: float,
    start_time: datetime,
    temp_info: str,
    field_info: str,
    flag: int,
    Sample_index: str,
    loc: str | None = None,
) -> None:
    """
    This code aborts the pulse delta sweep and reads the data from the buffer.
    The data is then saved to a CSV file with a unique filename.

```

```
    Args:
        GPIB_add (str): GPIB address of the source meter
        instrument (Keithley6221): Keithley6221 instrument instance
        start_I: starting value of current sweep, in A
        end_I: ending value of current sweep, in A
        step_I: current step, in A
        _delay: how long each point take, in s (not used in this function)
        start_time (datetime): time when the measurement starts
    """
    NI = int(np.round(abs((start_I - end_I) / step_I)) + 1)
    currents = np.linspace(start_I, end_I, NI)
    instrument.write("SOUR:SWE:ABOR")
    sleep(5)
    instrument.write("SENS:DATA?")
    for k in range(4):
        try:
            instrument.write("TRACe:DATA?")
            temp = instrument.read()
            break
        except Exception as e:
            if k == 3:
                print("Failed to read data after 4 attempts.")
                print(f"Error details: {e}")

    temp = temp.split(",")
    temp = np.reshape(temp, (NI, 2))
    temp = np.column_stack((currents, temp))

    folder_name = f"{temp_info}_{field_info}_{Sample_index}"
    file_start = (
        f"{temp_info}_{field_info}_{flag:03d}_"
        f"{start_I}A_{end_I}A_{step_I}A_{Sample_index}_"
    )
    filename = unique_filename(folder_name, file_start, loc)

    data = pd.DataFrame({"Current (A)": currents, "Voltage (V)": temp[:, 1]})
    multicolumns = pd.MultiIndex.from_tuples(
        [(str(start_time), "Current (A)"), (GPIB_add + Sample_index, "Voltage (V)")]
    )
    data.columns = multicolumns
    data.to_csv(filename)
```

```


# Example usage

# Setting up instrument and parameters
GPIB_add = "GPIB0::17::INSTR"
compliance = 40
delay = 1
PWidth = 10000e-6
SDelay = 1000e-6  # source delay > 500 s
temp_info = "50mK"
field_info = "20T-0deg"
sample_name = "S2"
keithley = Keithley6221(GPIB_add)
keithley.write("*RST")

# First sweep: -40mA to +40mA, step 0.5mA
flag = 1
start_0 = -40e-3
end_0 = +40e-3
step_0 = 5e-4
start_time, NI = PulseDelta_IV(
    GPIB_add, start_0, end_0, step_0, delay, compliance, PWidth, SDelay
)
sleep(delay * (NI + 7))
PulseDelta_abort_read(
    GPIB_add,
    keithley,
    start_0,
    end_0,
    step_0,
    delay,
    start_time,
    temp_info,
    field_info,
    flag,
    sample_name,
)

# Second sweep: 0mA to +25mA, step 0.2mA
flag = 2
start_0 = 0e-3
end_0 = 25e-3
```

```
step_0 = 2e-4
start_time, NI = PulseDelta_IV(
    GPIB_add, start_0, end_0, step_0, delay, compliance, PWidth,
       SDelay
)
sleep(delay * (NI + 7))
PulseDelta_abort_read(
    GPIB_add,
    keithley,
    start_0,
    end_0,
    step_0,
    delay,
    start_time,
    temp_info,
    field_info,
    flag,
    sample_name,
)

# Third sweep: +40mA to -40mA, step -0.5mA
flag = 3
start_0 = 40e-3
end_0 = -40e-3
step_0 = -5e-4
start_time, NI = PulseDelta_IV(
    GPIB_add, start_0, end_0, step_0, delay, compliance, PWidth,
       SDelay
)
sleep(delay * (NI + 7))
PulseDelta_abort_read(
    GPIB_add,
    keithley,
    start_0,
    end_0,
    step_0,
    delay,
    start_time,
    temp_info,
    field_info,
    flag,
    sample_name,
)

```

```
# Fourth sweep: 0mA to -25mA, step -0.2mA
flag = 4
start_0 = 0e-3
end_0 = -25e-3
step_0 = -2e-4
start_time, NI = PulseDelta_IV(
    GPIB_add, start_0, end_0, step_0, delay, compliance, PWidth,
       SDelay
)
sleep(delay * (NI + 7))
PulseDelta_abort_read(
    GPIB_add,
    keithley,
    start_0,
    end_0,
    step_0,
    delay,
    start_time,
    temp_info,
    field_info,
    flag,
    sample_name,
)
```

Listing 1: Code of functions used for performing various operations to investigate the memory effect of UTe_2.

References

1. Goldobin, E., Koelle, D., Kleiner, R. & Buzdin, A. Josephson junctions with second harmonic in the current-phase relation: Properties of φ junctions. *Phys. Rev. B* **76**, 224523 (2007).

2. Ambegaokar, V. & Halperin, B. I. Voltage Due to Thermal Noise in the dc Josephson Effect. *Phys. Rev. Lett.* **22**, 1364–1366 (1969).

3. Devoret, M. H., Martinis, J. M. & Clarke, J. Measurements of macroscopic quantum tunneling out of the zero-voltage state of a current-biased josephson junction. *Phys. Rev. Lett.* **55**, 1908–1911 (1985).

4. Martinis, J. M., Devoret, M. H. & Clarke, J. Experimental tests for the quantum behavior of a macroscopic degree of freedom: The phase difference across a josephson junction. *Phys. Rev. B* **35**, 4682–4698 (1987).

5. Tinkham, M. *Introduction to superconductivity* (Courier Corporation, 2004).

6. Shapiro, B. Y., Gitterman, M., Dayan, I. & Weiss, G. H. Shapiro steps in the fluxon motion in superconductors. *Phys. Rev. B* **46**, 8416–8420 (1992).

7. Chichinadze, D. V., Ribeiro, P., Shchadilova, Y. E. & Rubtsov, A. N. Transient phases and dynamical transitions in the post-quench evolution of the generalized Bose-Anderson model. *Phys. Rev. B* **94**, 054301 (2016).

8. Reichhardt, C. J. O., McDermott, D. & Reichhardt, C. Using principal component analysis to distinguish different dynamic phases in superconducting vortex matter. *Phys. Rev. B* **111**, 104508 (2025).

9. Kim, Y. B., Hempstead, C. F. & Strnad, A. R. Flux-Flow Resistance in Type-II Superconductors. *Phys. Rev.* **139**, A1163–A1172 (1965).

10. Blatter, G., Feigel'man, M. V., Geshkenbein, V. B., Larkin, A. I. & Vinokur, V. M. Vortices in high-temperature superconductors. *Rev. Mod. Phys.* **66**, 1125–1388 (1994).

11. Zhang, L. *et al.* Dimensionality of the reinforced superconductivity in UTe_2. *Nat. Commun.* **16**, 10308 (2025).

12. Wang, Y. *et al.* Observation of vortex stripes in UTe_2. *Nano Lett.* **25**, 12824–12831 (2025).

13. Sakai, H. *et al.* Field Induced Multiple Superconducting Phases in UTe_2 along Hard Magnetic Axis. *Phys. Rev. Lett.* **130**, 196002 (2023).

14. Shaffer, D., Chichinadze, D. V. & Levchenko, A. Superconducting diode effect in multiphase superconductors. *Phys. Rev. B* **110**, 184509 (2024).
