# Supplementary material for: Electrically controllable superconducting memory effect in UTe2
Source: Nature. 2026 Sep 16;657(8132):632–7. doi: 10.1038/s41586-026-11015-3 (PMC13581588; doi:10.1038/s41586-026-11015-3)
Supplement: Supplementary file 2 [file 41586_2026_11015_MOESM2_ESM.pdf]

nature portfolio

Peer Review File

Electrically-controllable superconducting memory effect in UTe2

Corresponding Author: Dr Alexander Eaton

Any redactions in this file are there to maintain patient confidentiality, the confidentiality of unpublished data, or to remove third-party material.

This file contains all reviewer reports in order by version, followed by all author rebuttals in order by version.

Version 0:

Reviewer comments:

Referee #1

(Remarks to the Author)
1. Summary: Zheyu Wu et al. have found an interesting hysteretic I-V characteristic in uranium ditelluride (UTe_2) that mimics that of a Josephson junction that can be explained by the RSJ model, especially for the case when the voltage vs current sweep exhibits a hysteretic behavior. Particularly, the researchers show that a "memory" effect exists depending on the current history, and therefore can be used for write and erasing functions. The authors present evidence that this effect occurs in the intermediate region between the SC1 and SC2 phases when the magnetic field is applied along the b-axis. They present evidence that the hysteresis is controlled by switching the applied current. They point to RSJ model-like behavior as evidence of the existence of two distinct order parameters. Unlike the Josephson junctions created by heterostructures, the researchers claim the novelty since this is originating within a single material, likely from the non-singular vortex dynamics.

2. Review: UTe_2 is perhaps one of the most "unconventional" superconductors known to date. This material is known to be a spin-triplet that is topologically non-trivial. Not only because the critical field of a spin-triplet superconductor should exceed the Pauli limit, UTe_2 has multiple superconducting phases at extremely high magnetic field ranges, where only high magnetic field facilities can have access to study this region. A particularly interesting regime is when the magnetic field is applied along the crystallographic b-axis. In this field direction, the superconducting state from 0 to about 14T (so called SC1 phase) and another superconducting state from 14T to 35 T (so called SC2 phase) exists. It is this intermediate regime between the two superconducting boundaries (which the authors name it SC1.5 or memory regime) where the researchers, Zheyu Wu et al., found an interesting Voltage vs Current and a hysteretic behavior. We believe this work is an important addition to literature for the UTe_2 community. However, we do not believe this work merits publication to the main Nature journal mainly because of a lack of understanding of the data and the unsubstantiated claims about the existence of two distinct order parameters and the non-singular vortex structures. The potential usefulness of this effect is, in our opinion, overstated, especially with the community not in a sufficient agreement of the superconducting order parameter. For example, it is not clear what distinguishes it from the myriad examples of similar phenomena that have appeared in the literature for half a century. The claim of novelty and importance seems to hinge on the fact that the effect occurs in a putative p-wave superconductor. Although we believe this work should be reported to the community, a few points must be addressed or further tested.

3. We believe the data has been taken carefully. However, the referees are still concerned with the following points that the researchers should additionally consider and address. This includes the methodology, validity, reliability, and suggested improvements. Also, we include possible references.

a. Possible Joule Heating artifact.
In Figure 1 (a), the so-called memory regime has a lower critical temperature than the low field superconducting phase (SC1) and the high field superconducting phase (SC2). This means that the sample can more easily exit the superconducting state (zero resistance or zero slope regime in the voltage vs current plot) by sample heating, especially when large current is flown through the sample and electrodes. We believe this can be a subtle effect that even a careful experimentalist can miss. Such Joule heating, for example, the local Joule heating from the contact resistance may increase

the temperature of a portion of the sample while this effect will not be picked up by the thermometer attached to the mixing chamber (if the researchers used a dilution refrigerator) or the He-3 pot (if the researchers used a He-3 system). The thermometer used for monitoring the sample must be as closely located near the sample as possible.
If such Joule heating is significant enough, some portions of the sample may be in a normal state rather than in a superconducting state. Then, an accidental SNS junction may be formed. In contrast, in the SC1 and SC2 state those states have a higher Tc and it is less vulnerable to create a normal state with the same amount of Joule heating.
The phase-slip center scenario must be discussed. If there exists a filamentary superconducting region in the sample, at a local region where the superconductivity breaks, the phase slips (or resets). This localized region creates heating and may show a similar behavior of the data the researchers observed (See Tinkham Figure 11.12).

b. Sample Quality Dependence: The figure panels of Figure 1 (b) are from Sample S1 and the figure panels from Figure 1 (c) is from Sample S2. In the methods section, the authors have reported that the Residual resistance ratio (RRR) value of S1 is higher than S2, implying that the quality of S1 sample is higher than S2. With that in mind, we notice that the current range at which the hysteresis exists is wider for S2. In Figure b where it is labeled “pump”, the hsystersis ends around ~5 A/cm2. In contrast, Figure c, has a hysteresis loop that exceeds 6 A/cm2. The fact that such hysteresis loop or “memory” effect persists at higher currents for lower quality samples worries the reviewer that this may be an extrinsic effect.

c. Two distinct order parameters. The claims regarding the existence of two "distinct superconducting order parameters" arising from the coexistence of SC1 and SC2 phases needs to be substantiated. The authors argue that the RSJ model physics requires two distinct order parameters. While it is noteworthy that this effect seems to only occur in the intermediate SC1-SC2 region of the phase diagram, this alone is not clear evidence of the existence of two superconducting order parameters. For example, can the authors rule out a scenario in which Jc is lowered in the intermediate SC1-SC2 phase but the system is described by a single order parameter? We emphasize that RSJ-like behavior alone is not sufficient to claim two order parameters given the wide applicability of this model to standard superconductors. In particular, it is easy to come up with counterexamples to the claim that a non-trivial minimum in the potential requires two order parameters—a non-trivial minimum would occur for a normal- and a pi-junction in parallel.

To elucidate the origin and mechanism of the tunneling barrier, we suggest that the authors study Jc as a function of temperature and field. It is well known that the temperature dependence of Jc follows a scaling law, and the associated scaling exponent can help understanding the origin.

4. While the manuscript positions this material as promising for future applications, the current work is far more significant for studying the fundamental nature of UTe$_{2}$. Discussing specific applications at this stage seems premature. We recommend shifting the focus to the fundamental physical insights provided by the data.

(Remarks on code availability)

Referee #2

(Remarks to the Author)
The authors present interesting experimental data demonstrating current-mediated memory effects in UTe2. From the perspective of vortex physics and neuromorphic computing, this is a potentially significant result.

The authors suggest vortex clustering as a possible underlying mechanism. This interpretation can be taken seriously. Notably, the paper reports that the effect appears at the parameter space corresponding to a boundary between two superconducting states, a regime in which earlier theoretical work has proposed that vortex clustering may occur generically. In particular, when a superconducting state of one symmetry transitions to a state of a different symmetry, a divergent coherence length mediates vortex attraction, leading to vortex clustering and strongly hysteretic behavior, consistent with the authors’ interpretation. In addition, vortex clusters have previously been reported by Y. Wang et al. in the same material, as cited by the authors, but in a different context.

Overall, the manuscript addresses an interesting problem and contains promising experimental results. However, in its current form, the presentation and interpretation require substantial clarification and strengthening before the work can be considered suitable for publication in Nature. The MS is now very far from a publishable form.

1 . The statement
“The complex vortex dynamics in this intermediate regime are reminiscent of those in superfluid 3 ^33He (Ref. 14),”
is quite vague and would benefit from greater specificity. It is not clear which aspects of the vortex dynamics are being compared, nor how Ref. 14 directly supports this analogy. In fact, Ref. 14 notes that vortex dynamics are beyond the scope of that review. The authors may wish to clarify whether a different reference was intended, or to explain more explicitly how the cited work is relevant to the present discussion.

2.
On page 5, the authors state:
“These observations have been taken to indicate a region of intermediate superconductivity between SC1 and SC2 (Ref.

43)."
This statement would benefit from a clearer definition. The term "intermediate superconductivity" is not well specified in this part of the text, and the reader is left to consult an external reference for its precise meaning. While it is reasonable to use terms such as "intermediate superconductivity" or "SC1.5," these should be explicitly and clearly defined within the manuscript itself, particularly given their central role in the interpretation.

3.
I also find the Discussion section insufficient in its present form. The phenomenological model introduced there would benefit from a more clearly articulated motivation and a more transparent connection to the experimental observations, or it could be dropped, and the paper can be presented as a set of experimental facts.

4.
In the Outlook, the authors draw several analogies to vortex states in superfluid 3 He. I do not think these analogies are supported by existing experimental data on UTe2. To date, magnetometry or other experiments have not reported observations of fractional vortices, nonsingular vortices, or domain walls, all of which would be expected to produce distinct signatures. In the absence of such evidence, the discussion in the concluding paragraph is overly speculative.

5.
Furthermore, the reference to quantum gravity lacks any basis and should be removed.

6.
Similarly, the paragraph on page 15 beginning with
"Perhaps the most exciting prospect for the technological exploitation of UTe2 lies in quantum computing"
is highly speculative and not directly supported by the results presented in the manuscript. The authors may wish to significantly temper this claim or remove it altogether.

7.
The authors need to give arguments if conventional vortex physics and pinning landscape/inhomogeneities could give a similar effect.

To summarize the experimental data is very interesting but the MS requires a significant revision.

(Remarks on code availability)

Referee #3

(Remarks to the Author)
I co-reviewed this manuscript with one of the reviewers who provided the listed reports.

(Remarks on code availability)

Referee #4

(Remarks to the Author)
The manuscript reports the observation of a non-equilibrium quantum state in the heavy-fermion strongly correlated superconductor UTe2. By applying a magnetic field to access an intermediate regime between two distinct superconducting phases, the authors demonstrate that direct current pulses can repeatedly drive the system into and out of a metastable state with a hysteresis loop in I-V measurement. This behavior effectively endows the system with memory functionality, suggesting potential applications in information storage.

At the same time, the results provide intriguing insights into the superconducting mechanism of Ute2 and, more broadly, into non-equilibrium phenomena in heavy-fermion superconductors. Overall, the reported phenomena are highly novel, experimentally well motivated, and of clear interest to the communities studying strongly correlated systems, unconventional superconductivity, and superconducting electronics. I therefore find the work suitable for publication. However, several points require clarification before publication.

1. The authors state that the observed memory functionality is an intrinsic property of UTe2, rooted in the superconducting order itself. While this is an appealing and potentially important claim, the physical picture underlying this phenomenon is not sufficiently developed.
Can the authors provide a more concrete microscopic or phenomenological explanation for this metastable state? Does it relate to the possible spin-triplet pairing? A clearer discussion would significantly strengthen the impact of the work.

2. Given the emphasis on memory functionality, it would be helpful to place the recent results in the context of existing superconducting memory devices. For example,
(1) ACS Nano 14 11002 (2020) https://doi.org/10.1021/acsnano.0c04405
(2) Nature Communications 14 4926 (2023) https://doi.org/10.1038/s41467-023-40654-7

3. In Fig. 3a, it is difficult to distinguish between the different curves clearly. Please consider reorganizing this figure, for example, by separating curves into multiple panels.

4. In Fig. 3, the hysteresis observed in the simulations appears significantly larger than that seen experimentally. This discrepancy deserves further explanation. What are the main assumptions or simplifications in the simulation that could lead to this difference?

5. Although the experiments are performed at very low temperatures, the use of direct current pulses raises the possibility of local heating or non-equilibrium quasiparticle accumulation. In particular, both the pulse duration and amplitude serve as tuning parameters for the observed memory effect, which makes it difficult to rule out a thermal origin of the hysteresis.

(Remarks on code availability)
This is the code for the Measurement Control Program. It is relatively straightforward, but due to the involvement of testing hardware control, I have not attempted to install or run it yet. However, the overall code structure appears to be reasonable.

Version 1:

Reviewer comments:

Referee #1

(Remarks to the Author)
Dear editors,

Referee 1 and 3 have read the author's response.

We believe that this is a remarkable finding in UTe2 that will again surprise the unconventional superconductor community. A Josephson junction-like and a memory device-like behavior in a bulk single crystal is quite surprising. In the previous round of the review process, the reason for Referee 1 and 3 reserving this work for publication in Nature was: 1) This experimental finding requires high magnetic fields. This has rather fundamental value than application value. We do not believe UTe2 can be used for practical memory devices, although a similar material that has much lower Sc1 and Sc2 can in the future be useful. 2) Low temperature and high magnetic field experiments require attention for possible artifacts created by heating, especially for experiments that are performed under very high electrical current applications.

From this round of review, we find that the authors have thoroughly addressed our initial concerns and also clarified the mis-appreciation referee 1 originally had. Referee 1 now agrees with the authors that this work is also not about elucidating the superconducting order parameter either. We believe this work will open the possibility to look for more similar quantum materials with better optimized conditions. Also, for the heating issue, I appreciate the authors that they have addressed the concerns we originally had. We are satisfied with the current version.

(Remarks on code availability)

Referee #2

(Remarks to the Author)
The manuscript reports very interesting and carefully executed measurements, and it reveals a new, very interesting effect with potential for future applications. The experimental work itself is valuable. However, in its present form, I do not consider the paper suitable for publication. Indeed, even at the level of a specialized journal such as Physical Review B, I would strongly recommend rejection in its current version.

My main concern is that the authors have done very little to address the criticism raised in the previous report. The revisions are very minimal, while the problems with the interpretation and presentation remain unaddressed.

Most importantly, I find the discussion section absolutely unacceptable in its present form because it repeatedly invokes concepts for which this paper provides no experimental evidence whatsoever. I do not understand the motivation for doing so, especially since these baseless claims do not strengthen the paper at all and instead not only mislead readers but also distract from what are, in my view, the genuinely interesting experimental results.

For this reason, I strongly recommend rejection from Nature in its present form, and likewise from the other Nature Portfolio journals at this stage. That said, because the data themselves are interesting and potentially important, I would strongly encourage the editors to give the authors one final opportunity to undertake a substantial revision. I believe the paper could become publishable, and potentially suitable for Nature, but only after a major revision of the discussion and a much more disciplined interpretation of the results.

What I strongly request is the removal, or very substantial rewriting, of all claims that have essentially no overlap with the actual data presented in the manuscript.

For example, the statement
“Our findings underscore the rich complexity of putatively p-wave vortex matter”
is not supported by the data. There is, in this paper, no evidence that the system realizes any established form of p-wave vortex matter, nor does the present work provide evidence for p-wave superconductivity itself. It may be a p-wave superconductor, but the results in the paper do not shed light on it.

Likewise, the passage
“Analogies to the exotic nonsingular vortex properties of ^{3}He have previously been made in the context of the UTe2 SC1.5 coexistence region … in which we observe the memory effect. Enticingly, such vortices may host Majorana fermions.”
should be removed. The reference to Majorana fermions is especially problematic. Many papers now repeat such statements almost as a ritual, but in the present case this manuscript provides no evidence at all bearing on Majorana physics. The material may or may not host Majorana modes, but these measurements do not speak to that question. Including such claims only introduces hype and weakens the credibility of the paper.

More generally, I see no evidence in this manuscript for any connection between vortex structures in UTe2 and those in superfluid ^{3}He. It is possible that there are broad phenomenological analogies at some level, but if the authors wish to invoke ^{3}He, then a clear and well-substantiated analogy must be made based on direct experimental data. Otherwise, these references to ^{3}He should be removed because they are presently misleading.

Similarly, the statement
“this coexistence or mixed SC1.5 region might exhibit exotic phenomena such as half-quantized vortices … or a nonsingular vortex state”
is not correct. Coexistence of order parameters does not by itself imply half-quantum vortices. In a coexisting pairing state, other more complicated, not necessarily half-quanta, types of fractional vortices may arise, such as those reported in recent works by Kathryn Moler’s and Yihua Wang’s groups. However, to date there is no evidence for such vortices in UTe2 from STM or scanning SQUID studies, despite relevant characterization by Moler, Wang, and other groups. Likewise, the proposed “coexistence” scenario would lead to vortex structures quite different from the nonsingular vortices of the high-symmetry models cited here. These distinctions matter and should not be blurred.

The authors do make a convincing case that the experiments reveal unusual vortex glassiness and memory, beyond the usual vortex glass. That, in my opinion, is an important and publishable result with potential for a Nature publication. By contrast, the resistively shunted junction (RSJ) model is not convincingly established as an appropriate description of the system. I am not strongly opposed to this model, but in its current form it seems oversimplified for the complex glassy state under discussion. My view is that the results would be stronger if they were presented primarily as a set of clean experimental facts, with minimal speculation and minimal theory. The RSJ modeling could then be reserved for a follow-up or parallel paper, unless the authors can convincingly demonstrate that it captures the relevant vortex-glass phenomenology in a non-superficial way.

Finally, this statement claims that there is independent support for the interpretation:
“More generally, this could also be interpreted as mesoscopic regions with different vortex species 44 whereby L would correspond roughly to the average diameter of the domains.”
Reference 44, as far as I can see, does not support the claim about “different vortex species.” Moreover, the proposed scenario is not correct, at least in this form. If two phases truly coexist, then either they phase separate-which there is no evidence for here, and, to my knowledge, no established model for in UTe2-or else different fractional vortex components are electromagnetically confined into a single composite vortex species, which was in fact the point of Ref. 44 as far as I can see. In that case, scanning SQUID detected one vortex type, not multiple species. In chiral p-wave superconductors, it is possible to have two types of coexistent vortices: single- and double-quanta, but that picture also does not agree with any experiments on vortices in UTe2.

That does not mean the general direction of the interpretation is wrong: composite vortices in the regimes with coexistence of order parameters, irrespective of the order parameter symmetry, have even more nontrivial interactions, rather generic clustering tendencies, and very glassy dynamics, compared to ordinary vortices, and there is substantial literature on this. One does not need multiple vortex species to obtain very glassy behavior; it can arise already in simpler models with a single, but complicatedly interacting, vortex type.

I also find unsupported the claim
“Furthermore, larger damping would be consistent with the scenario of a higher concentration of domain walls in the memory state, corresponding to a lower resistance $R \sim 1/\gamma$ in the vortex flow state.”
There is no evidence in this or any other paper for such domain walls in UTe2. Nor is there any discussion backing the claim in this sentence. At best, Refs. 56–58 discuss standard scenarios. The manuscript presents these ideas far too assertively given the absence of any support for domain walls. The same criticism applies to the final sentence of that paragraph.

The outlook section contains similar problems. For instance, the discussion of ^{3}He vortex phenomenology, including nucleation of one phase within the core of vortices of another phase, is again presented in a way that suggests relevance to UTe2 without any direct evidence. These references are misleading unless backed by concrete experimental support.

Likewise, the statement
"Given that the higher Bc2 of SC2 implies a shorter coherence length than for SC1, we speculate that similar phenomenology might occur in UTe2"
is not justified at all. A higher Hc2 alone does not provide meaningful support for that specific scenario.

I want to emphasize again that the Majorana-related statements are absolutely unacceptable in the present context. There is zero evidence in this paper for Majorana modes or for any model specifically supporting them. If UTe2 is interesting for Majorana-related reasons, that is a separate matter; these data do not illuminate it. The present work is interesting for other reasons, and the paper should stand on those.

I am equally concerned by the statement on another hyped topic:
"Furthermore, many of the properties of the intermediate SC1.5 regime could also be consistent with a finite-momentum paired state (Δ(r) ~ cos qr), which has been proposed to explain some experimental observations of UTe2 44,61"
This is not only unsubstantiated, but the manuscript does not even attempt to substantiate it. In its current form, this kind of statement should not appear. Moreover, the results in Refs. 44 and 61 also could be explained without a finite-momentum paired state.

In conclusion, the minimal revisions made in the second round are not acceptable. In its present form, the manuscript is not publishable. Nevertheless, because the experimental effect appears new and interesting, I would ask the editors to give the authors one final opportunity to produce a genuinely revised manuscript. Such a revision would need to remove unsupported claims, sharply reduce speculative discussion, and refocus the paper on the experimental evidence itself. If the authors do that, I believe the paper has a chance to become publishable.

(Remarks on code availability)

Referee #3

(Remarks to the Author)
I co-reviewed this manuscript with one of the reviewers who provided the listed reports.

(Remarks on code availability)

Referee #4

(Remarks to the Author)
I am satisfied that the authors have addressed my comments thoroughly and made the necessary revisions. The manuscript is now acceptable for publication.

(Remarks on code availability)
The code is now clearly commented.

Version 2:

Reviewer comments:

Referee #2

(Remarks to the Author)
I am not satisfied with the authors' reply. As in the previous round, the authors have addressed my criticisms only partially, and the manuscript still contains serious problems.

I did not claim that previous papers studied precisely the same region of the phase diagram. My point was instead that several papers are cited in a way that implies they support the interpretation advanced in this manuscript, whereas in fact they neither support nor contradict it.

The manuscript also continues to make baseless connections to fashionable topics such as FFLO states, fractional vortices, and related phenomena. These references may be appealing to those communities, but the experimental results in this paper do not support nor establish such connections.

Under ordinary circumstances, I would recommend rejection of this paper from any journal. However, the experimental data themselves appear interesting. My recommendation is therefore as follows:

I strongly recommend rejection of the manuscript in its present form from Nature or Nature Physics.
I would not object to publication in a more focused form, provided that the authors remove all non-scientific claims for which there is no experimental or theoretical support in this work.

My specific concerns are listed below.

1. Unsupported interpretation in terms of different vortex species

The central problem remains the authors' interpretation in terms of different species of vortices. This interpretation is based in part on citing a paper on a superfluid mixture, where two independent vortex species exist by construction. That model is not a model of superconductivity and has no relevance to the present system. This reference should be removed.

2. No support for two distinct kinds of vortices

There is no theoretical model and no experimental evidence in the manuscript supporting the existence of two distinct kinds of vortices in this superconducting material. I am not aware of any theory paper on superconductivity that would substantiate this interpretation. The corresponding claim and reference in the abstract should therefore be removed.

3. The theoretical modeling is disconnected from the experiment

The theoretical modeling section is not connected to the claimed interpretation in any meaningful way. It does not provide a basis for the central claims made in the manuscript. I strongly insist on removing the theory/modeling part from this paper.

The authors may, of course, publish such a theory separately, but in its present form it is far from sufficient for inclusion in a serious experimental paper in a high-profile journal.

4. The manuscript should remain focused on established experimental facts

If the authors wish, they may keep the cautious suggestion on page 13, beginning with "we propose," that the observed result may be related to the effect they discuss. However, the following paragraph and the discussion based on the RSJ model have no direct relationship to this interpretation and should be removed.

The manuscript should remain focused on well-established facts. In this case, those facts are experimental.

5. Unsupported discussion of diode effects

The references to diode effects on page 10 are not substantiated by the present data and should be removed. The manuscript does not provide sufficient evidence to make this connection.

6. Figure 3 is misleading

Figure 3 is highly misleading because it presents an unsubstantiated theoretical interpretation alongside experimental results. All theoretical components that are not directly supported by the data should be removed. Only experimentally established facts should remain.

7. Fractional vortices are not supported by the data

The references to fractional vortices and the corresponding citations on page 5 should be removed. The present paper has nothing to do with fractional vortices and sheds no light on their existence. Such vortices have not been observed in the other regions of the phase diagram of this material. The manuscript should focus on the data actually presented, rather than using the results as a pretext to comment on unrelated phenomena.

8. FFLO references are unjustified

The references to FFLO physics on page 24 should be removed. The data in this paper have no demonstrated connection to FFLO states.

Overall assessment

The main issue is not that the authors speculate, but that the manuscript repeatedly presents speculation in a way that suggests physical support where none exists. The experimental data is interesting, but the current framing overreaches substantially. The paper attempts to associate the results with a broad range of fashionable topics, including fractional vortices, FFLO physics, diode effects, and related ideas, without providing evidence that these phenomena are relevant.

This is not acceptable for a serious journal, especially not for Nature or Nature Physics. The manuscript could become suitable for publication only after substantial revision, with all unsupported theoretical and interpretive claims removed and the paper refocused on the experimental findings.

(Remarks on code availability)

Open Access This Peer Review File is licensed under a Creative Commons Attribution 4.0 International License, which permits use, sharing, adaptation, distribution and reproduction in any medium or format, as long as you give appropriate credit to the original author(s) and the source, provide a link to the Creative Commons license, and indicate if changes were made.
In cases where reviewers are anonymous, credit should be given to 'Anonymous Referee' and the source.
The images or other third party material in this Peer Review File are included in the article's Creative Commons license, unless indicated otherwise in a credit line to the material. If material is not included in the article's Creative Commons license and your intended use is not permitted by statutory regulation or exceeds the permitted use, you will need to obtain permission directly from the copyright holder.
To view a copy of this license, visit https://creativecommons.org/licenses/by/4.0/

Summary of major changes:

- Significantly revised Outlook section
- Significantly revised Discussion (now titled *Modelling the memory effect*)
- Two new main text paragraphs discussing why Joule heating artifacts can be ruled out
- New main text paragraph sketching out our microscopic picture of the memory effect
- 7 new supplementary figures
- New layout for Fig. 3
- Modified abstract

Referee #1 (Remarks to the Author):

1. Summary: Zheyu Wu et al. have found an interesting hysteretic I-V characteristic in uranium ditelluride (UTe2) that mimics that of a Josephson junction that can be explained by the RSJ model, especially for the case when the voltage vs current sweep exhibits a hysteretic behavior. Particularly, the researchers show that a "memory" effect exists depending on the current history, and therefore can be used for write and erasing functions. The authors present evidence that this effect occurs in the intermediate region between the SC1 and SC2 phases when the magnetic field is applied along the b-axis. They present evidence that the hysteresis is controlled by switching the applied current. They point to RSJ model-like behavior as evidence of the existence of two distinct order parameters. Unlike the Josephson junctions created by heterostructures, the researchers claim the novelty since this is originating within a single material, likely from the non-singular vortex dynamics.

We are grateful to the referee for expressing their interest in our discovery. We note however that one aspect of this point is slightly inaccurate: we do not claim simple RSJ model-like behavior as evidence for the existence of two distinct order parameters. Firstly, because the RSJ model needed to describe the memory effect is itself unconventional, and secondly because we consider scenarios other than with two distinct order parameters (as we discuss below in our reply to point 3). We will elaborate below on the large and growing body of evidence suggesting two distinct order parameters for the SC1 and SC2 phases of UTe2.

2. Review: UTe2 is perhaps one of the most "unconventional" superconductors known to date. This material is known to be a spin-triplet that is topologically non-trivial. Not only because the critical field of a spin-triplet superconductor should exceed the Pauli limit, UTe2 has multiple superconducting phases at extremely high magnetic field ranges, where only high magnetic field facilities can have access to study this region. A particularly interesting regime is when the magnetic field is applied along the crystallographic b-axis. In this field direction, the superconducting state from 0 to about 14T (so called SC1 phase) and another superconducting state from 14T to 35 T (so called SC2 phase) exists. It is this intermediate regime between the two superconducting boundaries (which the authors name it SC1.5 or memory regime) where the researchers, Zheyu Wu et al., found an interesting Voltage vs Current and a hysteretic behavior. We believe this work is an important addition to literature for the UTe2 community. However, we do not believe this work merits publication to the main Nature journal mainly because of a lack of understanding of the data and the unsubstantiated claims about the existence of two distinct order parameters and the non-singular vortex structures. The potential usefulness of this effect is, in our opinion, overstated, especially with the community not in a

sufficient agreement of the superconducting order parameter. For example, it is not clear what distinguishes it from the myriad examples of similar phenomena that have appeared in the literature for half a century. The claim of novelty and importance seems to hinge on the fact that the effect occurs in a putative p-wave superconductor. Although we believe this work should be reported to the community, a few points must be addressed or further tested.

We are grateful that the referee believes our work to be "an important addition to the literature". However, we respectfully disagree with the referee's characterization of the novelty aspect of our results. The novelty does not rely on the particular character of the paired state (whether it is p-wave or s-wave, triplet or singlet, topological or not). The novelty lies primarily in the memory effect itself, which to the best of our knowledge has not previously been seen in any other bulk superconductor. We do argue in turn that the memory effect is not possible in a conventional superconductor and thus provides evidence for an unconventional superconducting state in UTe2. While we discuss some plausible possibilities for such a state, we do not claim to resolve its precise nature, which is a task we leave to future studies.

As we shall detail below, the memory effect of UTe2 has several distinct characteristics that distinguishes it from the other effects the referee points to. We are grateful to the referee for inviting us to strengthen the explanation of these points of difference in our revised manuscript.

3. We believe the data has been taken carefully. However, the referees are still concerned with the following points that the researchers should additionally consider and address. This includes the methodology, validity, reliability, and suggested improvements. Also, we include possible references.

a. Possible Joule Heating artifact.
In Figure 1 (a), the so-called memory regime has a lower critical temperature than the low field superconducting phase (SC1) and the high field superconducting phase (SC2). This means that the sample can more easily exit the superconducting state (zero resistance or zero slope regime in the voltage vs current plot) by sample heating, especially when large current is flown through the sample and electrodes. We believe this can be a subtle effect that even a careful experimentalist can miss. Such Joule heating, for example, the local Joule heating from the contact resistance may increase the temperature of a portion of the sample while this effect will not be picked up by the thermometer attached to the mixing chamber (if the researchers used a dilution refrigerator) or the He-3 pot (if the researchers used a He-3 system). The thermometer used for monitoring the sample must be as closely located near the sample as possible.
If such Joule heating is significant enough, some portions of the sample may be in a normal state rather than in a superconducting state. Then, an accidental SNS junction may be formed.
In contrast, in the SC1 and SC2 state those states have a higher Tc and it is less vulnerable to create a normal state with the same amount of Joule heating.
The phase-slip center scenario must be discussed. If there exists a filamentary superconducting region in the sample, at a local region where the superconductivity breaks, the phase slips (or resets). This localized region creates heating and may show a similar behavior of the data the researchers observed (See Tinkham Figure 11.12).

We thank the referee for bringing this point to our attention. However, we do not believe that there is evidence of filamentary superconductivity in UTe2, as no characteristic phase slip steps have been seen in the IV curves. It is also unclear to us how such phase slips, or Joule heating, could explain the memory effect, even if accidental Josephson junctions are formed. To make

sure we are on the same page with the referee, as we understand Fig. 11.12 in Tinkham does not show hysteresis or memory (the normal curve is only shown to illustrate the asymptote of the IV curve). Though the IV curve in that figure is otherwise qualitatively similar to ours, our data are more naturally explained by vortex flow effects rather than phase slips, given the large magnetic fields.

The question of Joule heating is of course very important. In our measurements we were very careful to ensure this was not an issue – however, we regret not initially including all our relevant e.g. thermometry data, to convince a sceptical reader. In our revised manuscript, we have greatly expanded the supplement to show thermometry data during current pulses, which shows no meaningful changes during the measurements. We also show that the friction of the rotator mechanism produces much more considerable temperature changes, which require us to wait for the system to cool back to base after each rotation increment, before taking further data. By contrast, the heating imparted by the short current pulse measurement protocols is negligible.

Given the importance of considering Joule heating effects, in our revised manuscript we spend two paragraphs (immediately after the heading "Modelling the memory effect") explicitly considering and discussing potential heating effects. We give numerous arguments why we do not believe these to be relevant. These arguments include: the fact that the memory effect is most pronounced at low temperatures; the fact that the strength of the memory effect scales with the speed of turning off the current pulse that therefore anticorrelates with the amount of energy imparted to the sample; and the fact that the memory effect only occurs when in the pocket of phase space immediately underneath SC2 while upon rotating **B** more than 20 degrees away from [010] no memory effect is seen, and indeed no memory effect is seen within SC2.

Exploring further the referee's point of possibly forming an SNS section, let us work through carefully what this would imply. Consider that, after cooling the sample in zero field, a field is then applied. If enough heating were to occur to push some part of the sample from superconductivity into the normal state, when it later cools it then effectively field-cools and might trap some flux within that section, which might plausibly cause some hysteretic signal of some sort. However, there is then no way to return to the original (zero field cooled) state. The material might have hysteretically moved to some new state (field cooled), but without turning off the external field there is no way to return to the original (zero field cooled) state. Whereas, the UTe2 memory effect is easily reset with smooth current ramps, with the hysteresis behaving very differently for the naïve expectations of SNS sections. This simple line of reasoning adds yet another reason why we are very confident that heating effects are negligible in these measurements, and hence why we explain them with vortex reorganisation arguments.

b. Sample Quality Dependence: The figure panels of Figure 1 (b) are from Sample S1 and the figure panels from Figure 1 (c) is from Sample S2. In the methods section, the authors have reported that the Residual resistance ratio (RRR) value of S1 is higher than S2, implying that the quality of S1 sample is higher than S2. With that in mind, we notice that the current range at which the hysteresis exists is wider for S2. In Figure b where it is labeled "pump", the hsystersis ends around ~5 A/cm2. In contrast, Figure c, has a hysteresis loop that exceeds 6 A/cm2. The fact that such hysteresis loop or "memory" effect persists at higher currents for lower quality samples worries the reviewer that this may be an extrinsic effect.

We thank the referee for bringing this RRR dependence of the effect to our attention. The observation of sample dependence is in fact consistent with our modeling of the memory effect and the interpretation that it occurs due to differences in vortex pinning strength in different domain configurations. In particular, we expect that no memory effect (i.e., observation of two different critical currents before and after a current pump) would occur in perfectly clean sample, since in the absence of pinning all critical currents would be strictly zero. The memory effect would thus be expected to be enhanced in somewhat 'worse' samples. We now discuss this in the new Supplementary Information. Note, however, that disorder effects on vortex flow (and SC2 itself, see ref. 33) can be quite complicated, and the memory effect may also be detrimentally affected by much stronger disorder. Non-trivial sample dependence of the memory effect is thus unfortunately to be expected.

Importantly, as this discussion illustrates, extrinsic effects (i.e., presence of disorder) clearly are necessary for the memory effect to occur. However, the presence of extrinsic effects does not mean the absence of intrinsic effects. Since no memory effect has been observed in conventional superconductors, which are naturally disordered, and since within our phenomenological model unconventional terms are necessary to explain the memory effect, we argue that the memory effect requires both conventional extrinsic and unconventional intrinsic properties of the UTe2 samples. Again, we stress that for a ‘perfect’ type-II superconductor in a magnetic field, the critical current above Bc1 will be effectively zero, because of the lack of pinning. We argue that the observation of stronger memory correlating with stronger pinning sites fits very well with our proposed vortex reorganisation model.

It would be informative in future studies to carefully characterise the properties of the memory effect for different concentrations of defects. Indeed, by e.g. electron irradiation one could deterministically control the number of vortex pinning sites and quantitatively study how that affects the memory phenomena. However, this is beyond the scope of this initial discovery paper.

c. Two distinct order parameters. The claims regarding the existence of two "distinct superconducting order parameters" arising from the coexistence of SC1 and SC2 phases needs to be substantiated. The authors argue that the RSJ model physics requires two distinct order parameters. While it is noteworthy that this effect seems to only occur in the intermediate SC1-SC2 region of the phase diagram, this alone is not clear evidence of the existence of two superconducting order parameters. For example, can the authors rule out a scenario in which Jc is lowered in the intermediate SC1-SC2 phase but the system is described by a single order parameter? We emphasize that RSJ-like behavior alone is not sufficient to claim two order parameters given the wide applicability of this model to standard superconductors. In particular, it is easy to come up with counterexamples to the claim that a non-trivial minimum in the potential requires two order parameters—a non-trivial minimum would occur for a normal- and a pi-junction in parallel.

We thank the referee for raising this point. There is a multitude of evidence from other groups that points to two distinct order parameters for SC1 and SC2. However, we should have made this clear to the non-specialist reader. We have inserted a line in the main text to this effect: “There is a substantial, growing body of evidence that SC1 and SC2 possess distinct order parameters.8, 9, 36, 39, 40”

To further expand on this point, we would like to qualify that while we believe that two distinct order parameters are most likely a part of the explanation of the memory effect, in part due to

the abundance of evidence that SC1 and SC2 have two distinct order parameters (as cited above), this is not the only possible explanation that we consider. An alternative that we discuss is instead the presence of distinct vortex domains (i.e., with distinct vortex configurations), which is consistent with but does not require multiple order parameters.

Additionally, it is not simply the RSJ-like behavior that is the evidence of two distinct order parameters, but the unconventional RSJ-like behavior. Indeed, the conventional RSJ model would reproduce vortex flow IV curves seen in a conventional s-wave superconductor. The presence of a second metastable state that is clearly demonstrated by the memory effect, however, is not a generic feature of the RSJ model, and points to unconventional physics.

Here we should clarify that the conventional hysteresis in the RCSJ model and the memory effects are not identical: hysteresis in an underdamped Josephson junction is characterized by different retrapping and critical/switching currents (i.e., currents below and above which the system returns to and leaves the superconducting state, respectively). The memory effect refers instead to the presence of two different critical/switching currents depending on whether the system has been previously pumped with a current pulse or not. The memory state with the higher critical current does exhibit hysteresis, but the ground state in the absence of a current pump does not. While the final IV curves do look the same (both exhibit hysteresis loops), the sequence in which the branches are traversed is different: in a conventional hysteresis, the system always goes along the same branch as the current is swept up; in the memory effect, which branch the system goes along depends on past history, in particular whether there was a pump or not. This is illustrated in Fig. 1b.

Regarding the referee's assertion of a non-trivial minimum occurring for a normal and pi-junction in parallel, we respectfully disagree with this particular point. Our RSJ model is indeed based on a model of a ϕ junction which can be realized using 0- and pi- junctions in parallel [as demonstrated in PRL 109, 107002 (2012)]. But, first of all, realizing a pi-junction itself requires unconventional superconductivity or proximity of the superconductor to a ferromagnet. Second, the presence of both a normal and pi junctions in parallel clearly requires (at least two) domains with distinct superconducting properties. Contrary to the referee's assertion that this is a counter-example to the requirement for two order parameters, this example instead illustrates why the presence of two order parameters (or at least multiple domains of distinct nature) is likely needed to explain the memory effect, and why this is evidence of unconventional superconductivity in UTe2.

To elucidate the origin and mechanism of the tunneling barrier, we suggest that the authors study Jc as a function of temperature and field. It is well known that the temperature dependence of Jc follows a scaling law, and the associated scaling exponent can help understanding the origin.

In our revised supplement we include the critical current as a function of magnetic field for several temperatures:

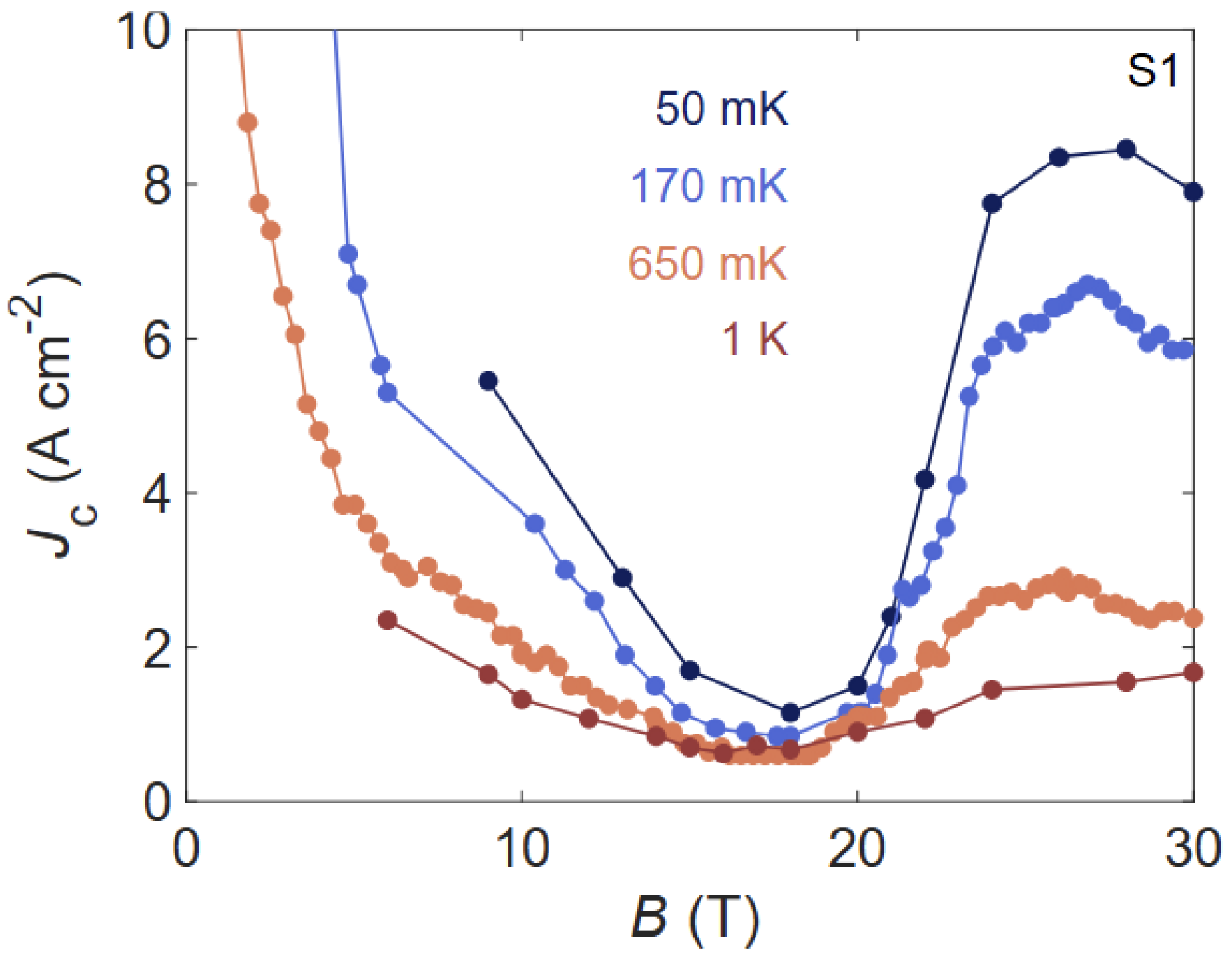

We also include additional data wherein we swept the field at various currents and temperatures:

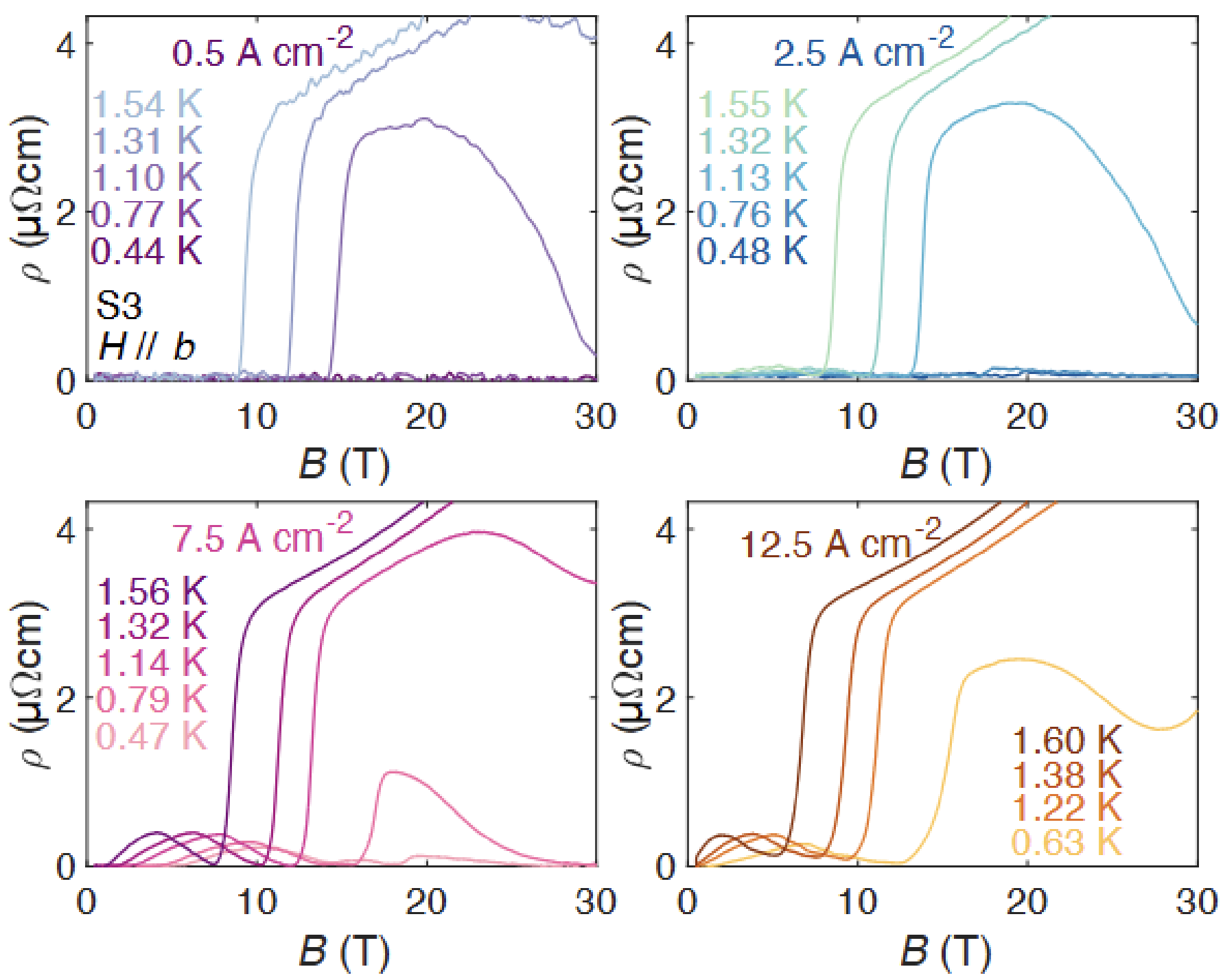

This behaviour is highly anomalous, with a complex non-monotonic profile of the effective resistivity with respect to magnetic field strength. As such, we do not think it would be appropriate to apply simple scaling assumptions to these datasets.

Instead, in order to more clearly explain our picture for where the memory effect originates from, we have added the below paragraph to the main text:

“We propose that the memory effect is due to competition between two different vortex species – one native to SC1, and the other from SC2. In the equilibrium state, the vortices arrange themselves in a highly ordered configuration to minimise entropy and energetic costs. Upon perturbing the system, current densities above Jc are applied, inducing a flux-flow regime in which both types of vortex are dissipatively travelling through the sample. If the perturbation is sufficiently sharp – for example, if the relaxation rate α is very fast – then when J drops back below Jc vortex flow suddenly halts and the vortices are abruptly locked into their new position. One can imagine a glassy vortex state has now been quenched in the material, which possesses much higher disorder than the original equilibrium vortex lattice. Higher disorder implies stronger pinning forces between vortices, and hence the higher Jc values that characterise the memory effect. The system can then be reset by smoothly raising J back above Jc before gradually ramping back down to zero current. This could be thought of as annealing the system, thereby returning to the equilibrium low-Jc state.”

We hope that the referee finds this simple picture quite intuitive and realistic, given the numerous aspects of the data that point towards such a scenario. We have tried, in our modelling section, to be as agnostic and make as few assumptions as possible, while being able to construct a minimal model that can qualitatively describe the interesting data we observed.

4. While the manuscript positions this material as promising for future applications, the current work is far more significant for studying the fundamental nature of UTe$_{2}$. Discussing specific applications at this stage seems premature. We recommend shifting the focus to the fundamental physical insights provided by the data.

We thank the referee for this advice. We have significantly revised the ending of the paper, with a much shortened outlook that briefly mentions how this effect could be technologically useful. We have dedicated much more text to explaining how we believe the memory effect is caused, and how that relates to the underlying microscopic physics of UTe2. We hope that, with these significant revisions, the referee now feels that the paper is suitable for publication.

Referee #2 (Remarks to the Author):

The authors present interesting experimental data demonstrating current-mediated memory effects in UTe2. From the perspective of vortex physics and neuromorphic computing, this is a potentially significant result.

The authors suggest vortex clustering as a possible underlying mechanism. This interpretation can be taken seriously. Notably, the paper reports that the effect appears at the parameter space corresponding to a boundary between two superconducting states, a regime in which earlier theoretical work has proposed that vortex clustering may occur generically. In particular, when a superconducting state of one symmetry transitions to a state of a different symmetry, a divergent coherence length mediates vortex attraction, leading to vortex clustering and strongly hysteretic behavior, consistent with the authors’ interpretation. In addition, vortex clusters have previously been reported by Y. Wang et al. in the same material, as cited by the authors, but in a different context.

Overall, the manuscript addresses an interesting problem and contains promising experimental results. However, in its current form, the presentation and interpretation require substantial clarification and strengthening before the work can be considered suitable for publication in Nature. The MS is now very far from a publishable form.
We thank the referee for highlighting our "promising experimental results" that address this "interesting problem" as a "potentially significant result".

1 . The statement
"The complex vortex dynamics in this intermediate regime are reminiscent of those in superfluid 3 ^33He (Ref. 14),"
is quite vague and would benefit from greater specificity. It is not clear which aspects of the vortex dynamics are being compared, nor how Ref. 14 directly supports this analogy. In fact, Ref. 14 notes that vortex dynamics are beyond the scope of that review. The authors may wish to clarify whether a different reference was intended, or to explain more explicitly how the cited work is relevant to the present discussion.

We agree that this phrasing led to unnecessary ambiguity. We have removed this line from the abstract. We have also significantly redrafted the outlook section, in which we state very clearly how we speculate there may be parallels between SC1 and SC2 with the A and B phases of 3He.

2.
On page 5, the authors state:
"These observations have been taken to indicate a region of intermediate superconductivity between SC1 and SC2 (Ref. 43)."
This statement would benefit from a clearer definition. The term "intermediate superconductivity" is not well specified in this part of the text, and the reader is left to consult an external reference for its precise meaning. While it is reasonable to use terms such as "intermediate superconductivity" or "SC1.5," these should be explicitly and clearly defined within the manuscript itself, particularly given their central role in the interpretation.

The referee raises an excellent point. In our revised manuscript we have amended how we introduce this terminology of "SC1.5" in the context of UTe2 (top of pg 5). We agree that "intermediate" was an ambiguous term, instead we now use "coexistence". The new phrasing is:

"Alongside signatures in the magnetic susceptibility, these observations have been taken to indicate a region of coexistence between the SC1 and SC2 states that hereafter we refer to as the SC1.5 regime"

3.
I also find the Discussion section insufficient in its present form. The phenomenological model introduced there would benefit from a more clearly articulated motivation and a more transparent connection to the experimental observations, or it could be dropped, and the paper can be presented as a set of experimental facts.

We thank the referee for their suggestion to improve the discussion section. Since the model is indeed phenomenological, the main purpose, besides offering a qualitative description of the memory effect, is to illustrate a plausible underlying physical mechanism behind the memory effect. For example, though we do not compute them, the drag and parameters fitting the critical current are consistent with a picture of vortex clustering and domain formation (with

increased pinning due to the domains in the metastable state plausibly leading to a larger critical current as well as a stronger drag on vortex motion). We hope that the explicit model thus helps to strengthen an otherwise qualitative explanation of the effect.

4.
In the Outlook, the authors draw several analogies to vortex states in superfluid 3 He. I do not think these analogies are supported by existing experimental data on UTe2. To date, magnetometry or other experiments have not reported observations of fractional vortices, nonsingular vortices, or domain walls, all of which would be expected to produce distinct signatures. In the absence of such evidence, the discussion in the concluding paragraph is overly speculative.

The referee raises sage points. In our revised draft we have significantly revised the wording and pitch of the Outlook, which now has only one paragraph briefly discussing 3He. We note that we are not the first to discuss possible nonsingular vortex state in UTe2 (see ref. 46). Given the field and temperature regime necessary to look for such phenomena, it is understandable why many experiments have not yet been performed examining this corner of the phase diagram. We hope to explore this further ourselves in future. We also have tried to make it very clear where we are speculating, particularly with our revised wording of: “Given that the higher $Bc2$ of SC2 implies a shorter coherence length than for SC1, we speculate that similar phenomenology might occur in UTe2.” We hope the referee feels that some small level of speculation is appropriate for an experimental discovery paper such as this.

5.
Furthermore, the reference to quantum gravity lacks any basis and should be removed.

This has been omitted in the revised manuscript.

6.
Similarly, the paragraph on page 15 beginning with
“Perhaps the most exciting prospect for the technological exploitation of UTe2 lies in quantum computing”
is highly speculative and not directly supported by the results presented in the manuscript. The authors may wish to significantly temper this claim or remove it altogether.

We thank the referee for their constructive criticism. We hope that the referee finds the new significantly shorter Outlook to be much more appropriate.

7.
The authors need to give arguments if conventional vortex physics and pinning landscape/inhomogeneities could give a similar effect.

Our phenomenological model is in principle consistent with both conventional and unconventional vortex physics. In particular, the switching between the stable and metastable configurations by applying a current pump or ramp can be explained in the model via conventional drag and pinning effects. The presence of a metastable configuration with a distinct depinning current, however, suggests an unconventional pinning landscape. In principle 'conventional' effects like vortex trapping can give rise to similar hysteretic behavior (see, e.g., Ref.[25], https://journals.aps.org/prb/abstract/10.1103/PhysRevB.39.4701), but the

fact that the memory effect only occurs in the SC1.5 region and not elsewhere suggests that conventional vortex physics alone is not sufficient to explain it.

The simplest explanation we suggest is domain formation in the SC1.5 region of the phase diagram, with the metastable state being a state with a larger number of domains (due to rapid domain nucleation upon a current pulse); the memory effect can then be understood if a current ramp anneals the system into a configuration with fewer domains. This moreover in principle explains why the depinning currents and the vortex drag are different between the stable and metastable states. In principle, if the domains are distinguished by some unconventional microscopic order parameters (e.g., if they are SC1 and SC2 regions), then conventional vortex physics is enough to account for the memory effect. On the other hand, the domains may well correspond to regions with distinct vortex clusters. As noted by the referee, this seems likely in UTe2 based on previously observed vortex clustering. It's possible and seems likely to us that both the microscopic order parameters and the vortices are unconventional, but additional data would be needed to determine this. We do believe that the memory effect shows that at least some aspect of superconductivity in the SC1.5 region must be unconventional, as no such effect has been reported in conventional bulk superconductors to the best of our knowledge.

To summarize the experimental data is very interesting but the MS requires a significant revision.

We thank the referee for their close reading and carefully scrutiny of our original submission, and for providing several helpful suggestions to improve our work. We hope that the referee would agree with us that the significant changes we have implemented have markedly improved the clarity and quality of our paper, and thus that our revised manuscript is now suitable for publication.

Referee #3 (Remarks to the Author):

I co-reviewed this manuscript with one of the reviewers who provided the listed reports.

Referee #4 (Remarks to the Author):

The manuscript reports the observation of a non-equilibrium quantum state in the heavy-fermion strongly correlated superconductor UTe2. By applying a magnetic field to access an intermediate regime between two distinct superconducting phases, the authors demonstrate that direct current pulses can repeatedly drive the system into and out of a metastable state with a hysteresis loop in I-V measurement. This behavior effectively endows the system with memory functionality, suggesting potential applications in information storage.

At the same time, the results provide intriguing insights into the superconducting mechanism of Ute2 and, more broadly, into non-equilibrium phenomena in heavy-fermion superconductors. Overall, the reported phenomena are highly novel, experimentally well motivated, and of clear interest to the communities studying strongly correlated systems, unconventional

superconductivity, and superconducting electronics. I therefore find the work suitable for publication. However, several points require clarification before publication.

We are grateful to Referees #3 & #4 for their positive appraisal of our work.

1. The authors state that the observed memory functionality is an intrinsic property of UTe2, rooted in the superconducting order itself. While this is an appealing and potentially important claim, the physical picture underlying this phenomenon is not sufficiently developed.
Can the authors provide a more concrete microscopic or phenomenological explanation for this metastable state? Does it relate to the possible spin-triplet pairing? A clearer discussion would significantly strengthen the impact of the work.

We provide an improved description of a phenomenological model of the effect in the discussion section. The essential picture is that a current pulse can lead to formation of multiple small domains that increase the strength of the vortex pinning, which makes this state both sufficiently stable so that the system does not relax to a ground state with fewer domains, and increases the critical current. An adiabatic (slow) decrease of the current from the normal state to the superconducting system, on the other hand, may act as annealing on the vortex domains and may allow the vortices to relax into a configuration with fewer domains.

The triplet pairing is not in itself essential to the model, but it may play an important role at a more microscopic level. In general, any microscopic modeling of the mixed state with vortex pinning forces would be quite complicated, and is beyond the scope of our work.

We hope that the referee finds our new explanatory paragraph to be much clearer:
"We propose that the memory effect is due to competition between two different vortex species – one native to SC1, and the other from SC2. In the equilibrium state, the vortices arrange themselves in a highly ordered configuration to minimise entropy and energetic costs. Upon perturbing the system, current densities above Jc are applied, inducing a flux-flow regime in which both types of vortex are dissipatively travelling through the sample. If the perturbation is sufficiently sharp – for example, if the relaxation rate α is very fast – then when J drops back below Jc vortex flow suddenly halts and the vortices are abruptly locked into their new position. One can imagine a glassy vortex state has now been quenched in the material, which possesses much higher disorder than the original equilibrium vortex lattice. Higher disorder implies stronger pinning forces between vortices, and hence the higher Jc values that characterise the memory effect. The system can then be reset by smoothly raising J back above Jc before gradually ramping back down to zero current. This could be thought of as annealing the system, thereby returning to the equilibrium low-Jc state."

We have also amended the abstract to bring this interpretation front and centre. We thank the referee for advising us to enhance the impact of our discovery in this way.

2. Given the emphasis on memory functionality, it would be helpful to place the recent results in the context of existing superconducting memory devices. For example,
(1) ACS Nano 14 11002 (2020) https://doi.org/10.1021/acsnano.0c04405
(2) Nature Communications 14 4926 (2023) https://doi.org/10.1038/s41467-023-40654-7
We thank the referee for bringing these works to our attention. We have revised our introduction, which now discusses these recent developments, alongside other similar work.

3. In Fig. 3a, it is difficult to distinguish between the different curves clearly. Please consider reorganizing this figure, for example, by separating curves into multiple panels.

We thank the referee for this suggestion – we have now expanded the temperature data into separate panels, which displays the temperature evolution much more clearly.

4. In Fig. 3, the hysteresis observed in the simulations appears significantly larger than that seen experimentally. This discrepancy deserves further explanation. What are the main assumptions or simplifications in the simulation that could lead to this difference?

We would like to emphasize that the theoretical modeling used arbitrary units, so it is not entirely valid to numerically compare the simulations against the experiment. The numerical values do happen to agree reasonably well if the arbitrary units are set to A cm^-2 (note that the axes in the figures have slightly different scales).

5. Although the experiments are performed at very low temperatures, the use of direct current pulses raises the possibility of local heating or non-equilibrium quasiparticle accumulation. In particular, both the pulse duration and amplitude serve as tuning parameters for the observed memory effect, which makes it difficult to rule out a thermal origin of the hysteresis.

Following the similar concerns raised by R1, in our revised text we now spend two paragraphs ruling out any such extrinsic heating artifacts, and in our new Supplementary Information we give a detailed discussion on this point. To recap from our answer to R1 above, our arguments revolve around: the fact that the memory effect is most pronounced at low temperatures; the fact that the strength of the memory effect scales with the speed of turning off the current pulse that therefore anticorrelates with the amount of energy imparted to the sample; and the fact that the memory effect only occurs when in the pocket of phase space immediately underneath SC2 while upon rotating **B** more than 20 degrees away from [010] no memory effect is seen, and indeed no memory effect is seen within SC2.

We hope the referee finds these arguments persuasive. The two new paragraphs in the main text are:
"Now we turn to considering the likely microscopic origin of the memory effect in UTe2. First, given that we are applying significant electrical current pulses at dilution fridge temperatures, we should consider any extrinsic heating effects that might be present. In the Supplementary Information we plot the measured temperature as a function of time during the current pulse measurement protocols, and give an extended discussion as to why we do not believe that any Joule heating effects significantly affect our measurements. One strong argument against any Joule heating artifacts comes from the sensitivity of the memory effect to the relaxation rate of the dc stimulus. In Fig. 2 we showed that stronger memory effect correlates with larger amplitude and duration tuning. For both of these, the larger stimulus correlates with larger energy deposited into the system. However, the memory effect is also strongly correlated with faster relaxation rates α, which anticorrelates with the total energy transferred into the system. If the memory effect were due to some relatively straightforward Bean-Livingston barrier or Bean model pinning effect process,50 this should be insensitive to how the current is turned off, depending only on the total energy imparted. The acute sensitivity of the memory effect to the turn-off speed instead implies some dynamic re-organisation or annealing of vortex domains, due to some interesting intrinsic physical property or properties of UTe2.

Further arguments against heating effects stem from the fact this phenomenon is only observed in the narrow regime of $B-\theta$ space directly underneath SC2, whereas any extrinsic heating

phenomena should likely be present throughout SC1 and SC2. The memory effect is most pronounced at the lowest temperatures, and quickly diminishes upon warming (Fig. 3), providing perhaps the strongest argument against any heating-induced artifacts. Instead, this strongly indicates that the observed hysteretic $V\ (J)$ behaviour is due to some intrinsic low-energy phenomenon, with an energy scale significantly lower than Tc."

We hope that the referee finds this discussion adequate, and with the incorporated changes, that our work is now ready for publication.

Referees' comments:

Referee #1 (Remarks to the Author):

(see also attachment)

Referee 1 and 3 have read the author's response.

We believe that this is a remarkable finding in UTe2 that will again surprise the unconventional superconductor community. A Josephson junction-like and a memory device-like behavior in a bulk single crystal is quite surprising. In the previous round of the review process, the reason for Referee 1 and 3 reserving this work for publication in Nature was: 1) This experimental finding requires high magnetic fields. This has rather fundamental value than application value. We do not believe UTe2 can be used for practical memory devices, although a similar material that has much lower Sc1 and Sc2 can in the future be useful. 2) Low temperature and high magnetic field experiments require attention for possible artifacts created by heating, especially for experiments that are performed under very high electrical current applications.

From this round of review, we find that the authors have thoroughly addressed our initial concerns and also clarified the mis-appreciation referee 1 originally had. Referee 1 now agrees with the authors that this work is also not about elucidating the superconducting order parameter either. We believe this work will open the possibility to look for more similar quantum materials with better optimized conditions. Also, for the heating issue, I appreciate the authors that they have addressed the concerns we originally had. We are satisfied with the current version.

We thank the referees for their careful reading of the manuscript, and helpful suggestions for improving it. We are grateful that they now find our work ready for publication in *Nature*.

Referee #2:

The manuscript reports very interesting and carefully executed measurements, and it reveals a new, very interesting effect with potential for future applications. The experimental work itself is valuable. However, in its present form, I do not consider the paper suitable for publication. Indeed, even at the level of a specialized journal such as Physical Review B, I would strongly recommend rejection in its current version.

My main concern is that the authors have done very little to address the criticism raised in the previous report. The revisions are very minimal, while the problems with the interpretation and presentation remain unaddressed.

Most importantly, I find the discussion section absolutely unacceptable in its present form because it repeatedly invokes concepts for which this paper provides no experimental evidence whatsoever. I do not understand the motivation for doing so, especially since these baseless claims do not strengthen the paper at all and instead not only mislead readers but also distract from what are, in my view, the genuinely interesting experimental results.

For this reason, I strongly recommend rejection from Nature in its present form, and likewise from the other Nature Portfolio journals at this stage. That said, because the data themselves are interesting and potentially important, I would strongly encourage the editors to give the authors one final opportunity to undertake a substantial revision. I believe the paper could become publishable, and potentially suitable for Nature, but only after a major revision of the discussion and a much more disciplined interpretation of the results.

We thank the referee for recognising the value of this work, and regret that our previous revisions, despite what we hoped, were not to the referee's satisfaction. We are also grateful that the referee is affording us another opportunity to address those concerns. We have removed the vast majority of the speculative portions of the text, mostly towards the front and back of the manuscript, which we hope addresses the core of the referee's concerns, and address more specific points (which we numbered for convenience) in our reply below.

We would like to politely point out at this point that perhaps the referee has made some mis-apprehension in their assessment of the previous research literature of UTe2. At numerous points in their challenges below they refer to microscopic studies of the vortex studies of UTe2 (from STM and scanning SQUID experiments). However, to date none of these studies examined the vortex properties of UTe2 in the memory effect region of the phase diagram. They are all performed exclusively within SC1, at different field strength/field angle/temperature regions of the complex phase diagram that lie relatively far away from the memory region we uncover here. Thus, none of our inferences and speculatory points concerning the possible vortex properties in the memory region go against observations from microscopic measurements – simply because no such measurements yet exist. It will be very interesting to go exploring the microscopic vortex properties in the vicinity of the memory regime our present work has uncovered, which we intend to pursue in subsequent work.

Before proceeding, we should note that we feel that a number of the referee's concerns are perhaps more philosophical in nature, rather than physical. The referee has very few comments pertaining to our experimental procedure or indeed the data itself. Instead, the referee appears to have very strong opinions regarding the structure of the

discussive elements of our manuscript. We note that the other three referees found our Discussion and Outlook to be appropriate.

In our view, here we are presenting an experimental discovery paper – and we feel we are entirely justified to have some room for speculation in it, provided we signpost very clearly where we are being speculatory. The referee acknowledges that many other groups' papers in our field discuss e.g. Majorana physics. In so doing, the referee acknowledges that this is standard current scientific practice – the referee simply has strong subjective opinions on this practice, and its appropriateness. We would like to politely argue we feel this is very much a question of taste, rather than on the soundness, novelty, or importance of our experimental results.

However, this being said, we do indeed agree with the referee on several of these philosophical points that they raise. I cannot speak on behalf of all coauthors, but personally (A.G.E.) I very much agree that there is currently a regrettable level of hype and overselling within our community. In this revised manuscript, we have therefore made a very honest attempt to slim down to the key physical points that are most pertinent (and directly relevant) to our experimental work presented herein. **We have removed all references to Majorana physics, and to superfluid He-3**. We appreciate that the referee does not like our minimal model we invoke to give a possible phenomenological description of our experimental results. However, numerous other colleagues have positively commented upon it while sharing our work with them – so again, this very much appears to be a matter of taste. We would politely reemphasise that we are not at all claiming that *this theoretical model is definitely true and capturing all the physics present here* – far from it. In the 'Modelling the memory effect' section we now explicitly state: "We propose that the memory effect may be due to…" to make it abundantly clear that this is merely a simple model that might explain some of this phenomenology. We are grateful to the referee for their positive appraisal of our experimental discovery. We would politely suggest that a reader, once getting to this stage of our paper where we present the model, will have seen the 4 main figures of data and will very much be in the position to decide for themselves whether they do/do not like our little minimal model; and in no way does that detract or diminish the novelty and importance of the experimental result itself. Instead, we would argue that it simply provides a point of departure for constructing future more rigorous theoretical descriptions – and provides a grain of inspiration, we hope, for some of the numerous other groups working on UTe2 to come along and pursue further experimental studies in this remarkable region of the phase diagram where the memory effect is manifested.

1. What I strongly request is the removal, or very substantial rewriting, of all claims that have essentially no overlap with the actual data presented in the manuscript.

We have removed any mentions of phenomena not directly linked to the data, and rewrote the text in relation to those phenomena that are linked to the data.

2. For example, the statement

“Our findings underscore the rich complexity of putatively p-wave vortex matter” is not supported by the data. There is, in this paper, no evidence that the system realizes any established form of p-wave vortex matter, nor does the present work provide evidence for p-wave superconductivity itself. It may be a p-wave superconductor, but the results in the paper do not shed light on it.

This has been deleted.

3. Likewise, the passage
“Analogies to the exotic nonsingular vortex properties of ^{3}He have previously been made in the context of the UTe2 SC1.5 coexistence region … in which we observe the memory effect. Enticingly, such vortices may host Majorana fermions.”
should be removed. The reference to Majorana fermions is especially problematic. Many papers now repeat such statements almost as a ritual, but in the present case this manuscript provides no evidence at all bearing on Majorana physics. The material may or may not host Majorana modes, but these measurements do not speak to that question. Including such claims only introduces hype and weakens the credibility of the paper.

We have removed all references to Majoranas and helium-3.

4. More generally, I see no evidence in this manuscript for any connection between vortex structures in UTe2 and those in superfluid ^{3}He. It is possible that there are broad phenomenological analogies at some level, but if the authors wish to invoke ^{3}He, then a clear and well-substantiated analogy must be made based on direct experimental data. Otherwise, these references to ^{3}He should be removed because they are presently misleading.

This has been omitted.

5. Similarly, the statement
“this coexistence or mixed SC1.5 region might exhibit exotic phenomena such as half-quantized vortices … or a nonsingular vortex state”
is not correct. Coexistence of order parameters does not by itself imply half-quantum vortices. In a coexisting pairing state, other more complicated, not necessarily half-quanta, types of fractional vortices may arise, such as those reported in recent works by Kathryn Moler’s and Yihua Wang’s groups. However, to date there is no evidence for such vortices in UTe2 from STM or scanning SQUID studies, despite relevant characterization by Moler, Wang, and other groups. Likewise, the proposed “coexistence” scenario would lead to vortex structures quite different from the nonsingular vortices of the high-symmetry models cited here. These distinctions matter and should not be blurred.

We agree with the referee that we should be more careful in our wording in this case, and we changed half-quantized to fractional to include the more general possibility. We would like to point out, however, that the vortex imaging in UTe2 by Moler and Wang

groups have only been carried out in low fields (0.06 T and 0.5 T, respectively), deep in the SC1 phase, whereas the memory effect occurs between 7 and 20 T. Imaging by Yang et al. [41] (up to 5 T) and Sharma et al. [42] (up to 8 T) are closer to the SC1.5 region and see interesting signatures of inversion symmetry breaking in the shape of the vortices, but they still do not quite reach into the temperature-field regime of the memory state due to wrong field orientation (we also note that the extent of the SC1.5 region is sample-dependent due to disorder effects).

In general, therefore, little is known experimentally about vortices in the SC1.5 region, except that the vortex transport is unusual, and which our data shows exhibits the memory effect. It is therefore not unreasonable to suppose that the vortex structures in this region are also unusual, especially given the large body of evidence in the literature suggesting some form of coexistence between SC1 and SC2 [see e.g. https://arxiv.org/abs/2603.17905], which the referee points out is a necessary precondition for realizing fractional vortices.

Likewise, the non-singular vortices were considered precisely in the SC1.5 region of the UTe2 phase diagram by Tokiwa et al. [44] to explain unusual vortex transport, so we do not think it is inappropriate to mention this possibility.

6. The authors do make a convincing case that the experiments reveal unusual vortex glassiness and memory, beyond the usual vortex glass. That, in my opinion, is an important and publishable result with potential for a Nature publication. By contrast, the resistively shunted junction (RSJ) model is not convincingly established as an appropriate description of the system. I am not strongly opposed to this model, but in its current form it seems oversimplified for the complex glassy state under discussion. My view is that the results would be stronger if they were presented primarily as a set of clean experimental facts, with minimal speculation and minimal theory. The RSJ modeling could then be reserved for a follow-up or parallel paper, unless the authors can convincingly demonstrate that it captures the relevant vortex-glass phenomenology in a non-superficial way.

The main criterion for establishing a phenomenological model is that it properly captures the phenomenology, and given the reasonable agreement between the data and the simulation we believe the RSJ model meets this criterion. We do of course agree that our model does not capture the precise nature pertaining to the complex structure of the glassy state, as any such model would require running complex numerical simulations, but this is beyond the scope of our work: we agree with the referee that the theory should instead be minimal.

There are three main aspects of the model that we believe describe the memory effect in a non-superficial way, and which moreover allow us to draw some conclusions about the possible origin of the effect. The two most important of these are the presence of two distinct minima in the free energy landscape corresponding to the ground state and the metastable state, and the fact that a current pulse switches between the two states.

The latter implies that the two states must correspond to two different values of the parameter φ that couples to the current J (a term of the form φJ in the free energy). In the simplest interpretation, φ is the SC phase across the sample, but it is also related to vortex configurations, see our discussion of point 8. In any interpretation, a state with a non-trivial φ must break translation symmetry, which is the main conclusion we draw from the model. From the presence of two minima, on the other hand, we can also conclude that there is likely a competition or coexistence of two different states in the memory region, which suggests a scenario with phase separation or multiple vortex species, etc. We discuss these points in more detail in our response to points 8 and 9.

A third aspect the model captures, which is not as salient, is the fact that the critical current as well as the damping in the metastable state are higher than those in the ground state. This suggests that vortex pinning and damping are stronger in the metastable state, and this is consistent with an increased number of domain walls in that state. This of course does not prove the presence of domains, but it favors that scenario. We discuss this in more detail in our response to points 9 and 10.

7. Finally, this statement claims that there is independent support for the interpretation: "More generally, this could also be interpreted as mesoscopic regions with different vortex species 44 whereby L would correspond roughly to the average diameter of the domains."
Reference 44, as far as I can see, does not support the claim about "different vortex species."

We apologize for the editing mistake, the intention was to mention both the possibility of domains with different vortex species (for example, SC1 and SC2 domains; more exotic structures in which vortices of the two types form lattices are also possible [Phys. Rev. Lett. **122**, 045301]), as well as vortex stripes or clusters that act as effective domains, with L then being the distance between the stripes, which is why we made that citation. We thank the referee for identifying this error, which we address further in our response to point 8.

8. Moreover, the proposed scenario is not correct, at least in this form. If two phases truly coexist, then either they phase separate-which there is no evidence for here, and, to my knowledge, no established model for in UTe2-or else different fractional vortex components are electromagnetically confined into a single composite vortex species, which was in fact the point of Ref. 44 as far as I can see. In that case, scanning SQUID detected one vortex type, not multiple species. In chiral p-wave superconductors, it is possible to have two types of coexistent vortices: single- and double-quanta, but that picture also does not agree with any experiments on vortices in UTe2.

Again, we should stress that *there have so far not been any direct measurements of vortices in UTe2 in the memory effect region of the phase diagram*. All STM and scanning SQUID studies reported to data have been at lower fields/higher temperatures

than our current-voltage measurements reported here, in which we observe the memory effect. The question of phase separation is somewhat controversial, with numerous recent reports e.g. https://arxiv.org/abs/2604.25896 https://arxiv.org/abs/2603.17905 - here in this work we remain agnostic regarding this.

Furthermore, we believe that on this overall point we are not in fact in disagreement with the referee, but we have not been sufficiently clear about the scenarios we are considering (and our error in point 7 likely led to additional confusion). We apologise for this, and have expanded that description for clarity.

We fully agree that there are in essence three possibilities mentioned by the referee: phase separation, a uniform phase possibly with complex vortex clusters (as in ref 44), or multiple coexisting vortices species [e.g. Scientific Reports volume 5, Article number: 17540 (2015)]. Our intention was simply to mention all three as consistent with our model, which hopefully is more clear in the new manuscript. **We have kept the modelling deliberately agnostic with respect to these possible microscopic pictures.** We merely present a simple, minimal model, by which we can explain the phenomenology of the observed effect.

The main point is that the RSJ model establishes that the metastable state responsible for the memory effect must break some translation symmetry of the ground state (in relation to point 6., we believe this is one of the main values of the model). This is because the metastable state must correspond to a non-trivial minimum of the (phenomenological) SC phase parameter φ, namely φ=π (it must be π in order to preserve inversion symmetry of the state, as seen in experiment). It's important to emphasize that the phase φ in phenomenological, the only requirement of the model is that dφ/dt is proportional to the voltage while the supercurrent is a function of φ. This means φ is proportional to the SC phase across the sample, but it is also related to the vortex lattice configurations: essentially the same RSJ-like equations encode vortex dynamics, see in Eqs (1-3) in Ref. [53], among many other examples.

In the most direct interpretation of φ as the SC phase across the sample, the metastable state is in fact a finite momentum-paired state with Δ~e^{i φ r/L}, where r is the position along the sample and L is the sample length (see our related response to point 14). The Cooper pair momentum in this state is q=φ/L, and inversion symmetry of the state implies that Δ(r) ~ cos qr (the so-called LO-type state). We do not believe this literal interpretation of the model is very likely to be relevant to the experiment, which is why we do not refer to FFLO states except in passing and in the context of other UTe2 works. Since φ also encodes vortex configurations and other spatial inhomogeneity, the only conclusion we can draw from the model is that the metastable state breaks translation symmetry with some characteristic length scale l~L/φ.

The three scenarios (phase separated domains, vortex clusters, or some lattice structure of coexisting vortex species) can all provide this relevant length scale (domain size, distance between vortex stripes, etc.). In our view, phase separation into multiple domains is the simplest and most likely scenario, as we explain further in our response

to point 9, but we do not rule out any other scenario. It should be pointed out, for example, that UTe_2 in general and the SC1.5 phase in particular are contenders for a chiral p-wave SC, as discussed widely in the literature. There are still relatively few experiments on vortex flow effects in the SC1.5 region besides our own (and as we have stated, **no** direct imaging studies of the microscopic vortex properties), so our results add plausibility to the multi-species picture, though of course do not conclusively prove it. If multiple vortex species do exist, they may form complicated vortex lattices that could also be consistent with translation-symmetry breaking metastable states, see added reference [Phys. Rev. Lett. **122**, 045301].

In short, and to reiterate, we agree with the referee that no conclusive evidence exists to firmly rule in/out any of the possible scenarios, which is why we discuss them on equal footing. It is not our intention to suggest that the experiment proves any one of these scenarios, only that it is consistent with all of them.

9. That does not mean the general direction of the interpretation is wrong: composite vortices in the regimes with coexistence of order parameters, irrespective of the order parameter symmetry, have even more nontrivial interactions, rather generic clustering tendencies, and very glassy dynamics, compared to ordinary vortices, and there is substantial literature on this. One does not need multiple vortex species to obtain very glassy behavior; it can arise already in simpler models with a single, but complicatedly interacting, vortex type.

Here we again agree with the referee. Vortex clustering, e.g. into stripes, would provide an explanation of a state with broken translation symmetry. However, this is not the only thing that needs to be explained in the memory effect: one has to further explain the presence of two stable states (the ground state *and* the metastable state). We cannot rule out that this is possible with a single complicatedly interacting vortex type in an otherwise uniform phase, but we are not aware of any examples in the literature that demonstrated a current-pulse controlled depinning current in such phases, glassy or otherwise. We would be greatly indebted to the referee if they point us to the relevant literature we may not be aware of.

In our view, the presence of two minima of the free energy is more naturally explained by the presence of two competing or coexisting states. This favours the scenarios with phase separation into domains with different SC orders or with different vortex species, the former being the simplest explanation in our opinion. The phase separation scenario is also favoured by the plethora of experimental evidence that SC1 and SC2 are two distinct phases.

10. I also find unsupported the claim
“Furthermore, larger damping would be consistent with the scenario of a higher concentration of domain walls in the memory state, corresponding to a lower resistance $R \sim 1/\gamma$ in the vortex flow state.”
There is no evidence in this or any other paper for such domain walls in UTe_2. Nor is there any discussion backing the claim in this sentence. At best, Refs. 56–58 discuss

standard scenarios. The manuscript presents these ideas far too assertively given the absence of any support for domain walls. The same criticism applies to the final sentence of that paragraph.

To clarify, we cited those references in relation to the fact that larger damping corresponds to lower resistance (this is well-known but may be counterintuitive to some readers). We do not cite them in relation to the existence or otherwise of domain walls. However, it is also well-known and more intuitive that domain walls do act as a source of vortex pinning. As stated in our response to point 9, we believe the presence of domains in the SC1.5 region to be the most likely mechanism for the memory effect, and this is further supported by the fact that in this scenario we can qualitatively explain why the critical current in the metastable state is higher than in the ground state. This is a further, if less concrete, illustration of the value of the RSJ model, since it requires increased damping in the metastable state to explain the memory effect (such that the system tends to stay in the metastable state upon a current pump).

We reiterate, we have now reworded the beginning of that section to start "We propose that the memory effect may be due to…" and leave it to the reader to draw their own informed conclusions based on the >10 figures of data we present throughout the manuscript.

11. The outlook section contains similar problems. For instance, the discussion of ^{3}He vortex phenomenology, including nucleation of one phase within the core of vortices of another phase, is again presented in a way that suggests relevance to UTe_2 without any direct evidence. These references are misleading unless backed by concrete experimental support.

12. Likewise, the statement
"Given that the higher B_{c2} of SC2 implies a shorter coherence length than for SC1, we speculate that similar phenomenology might occur in UTe_2"
is not justified at all. A higher H_{c2} alone does not provide meaningful support for that specific scenario.

13. I want to emphasize again that the Majorana-related statements are absolutely unacceptable in the present context. There is zero evidence in this paper for Majorana modes or for any model specifically supporting them. If UTe_2 is interesting for Majorana-related reasons, that is a separate matter; these data do not illuminate it. The present work is interesting for other reasons, and the paper should stand on those.

These are removed.

14. I am equally concerned by the statement on another hyped topic:

“Furthermore, many of the properties of the intermediate SC1.5 regime could also be consistent with a finite-momentum paired state ($\Delta(r) \sim \cos qr$), which has been proposed to explain some experimental observations of UTe_2 44,61”
This is not only unsubstantiated, but the manuscript does not even attempt to substantiate it. In its current form, this kind of statement should not appear. Moreover, the results in Refs. 44 and 61 also could be explained without a finite-momentum paired state.

On this point, we are somewhat confused with the referee’s objection here. It has been clearly shown from STM measurements that UTe_2 has a tendency towards pair-density-wave ordering [Nature 618, 921–927 (2023)]. We hope that our discussion of the RSJ model above provided some substantiation. This is simply an observation that in the RSJ model the state with $\varphi=\pi$ is precisely a finite-momentum paired state. We intentionally avoided using the overhyped FFLO terminology since this need not be a very exotic state. An important reason is that, as discussed in our response to point 8, φ is a phenomenological parameter and we do not want to over-interpret it as the literal phase of the microscopic order parameter, though it must, on general grounds, be related to it. To avoid sounding too exotic, we replaced ‘finite-momentum pairing’ with ‘spatially modulated (or finite-momentum) pairing.’ Finite-momentum pairing and spatially modulated pairing are of course equivalent.

Reference [44] may help illustrate what we mean: the presence of vortex stripes in fact in general implies that the underlying order parameter must have an oscillatory component in real space that goes as $\Delta(r) \sim \cos qr$, with $1/q$ corresponding to the distance between the stripes (on top of the short-scale inhomogeneity of $\Delta(r)$ due to the presence of vortices themselves). We would not call such a state an FFLO state (since the origin of the stripes is an interaction between the vortices), but it is technically a state with finite-momentum pairing. We hope this clarifies what we mean by finite-momentum pairing in the broad sense. Note that any state with a supercurrent is also in general a finite-momentum paired state; finite-momentum pairing is only exotic if it occurs in the ground state, which we are not claiming.

Regarding reference [61], we simply refer to the authors’ interpretation of their data (they do refer more specifically to the more exotic pair density wave), and we make no claim on its validity. A similar possibility of an FFLO state explaining unusual vortex dynamics has been independently made in [PRL **130**, 196002], which we now cite in this section as well. It both cases the interpretation was made in the context of the SC1.5 region, and given the result of our model we believe it is not unfair to mention.

In conclusion, the minimal revisions made in the second round are not acceptable. In its present form, the manuscript is not publishable. Nevertheless, because the experimental effect appears new and interesting, I would ask the editors to give the authors one final opportunity to produce a genuinely revised manuscript. Such a revision would need to remove unsupported claims, sharply reduce speculative discussion, and refocus the paper on the experimental evidence itself. If the authors do that, I believe the paper has a chance to become publishable.

In summary, we hope that our significant revisions (mostly deletions!) make the reviewer feel much more comfortable with the discussive elements of our manuscript. Once again, we would like to firmly underline that at no point do we claim to have 'solved' the origin of the interesting memory effect phenomenon – rather, we merely provide some ideas as to what appears, to us, to be a reasonable explanation of the phenomenology that can reproduce the experimental result qualitatively accurately.

We thank the referee for their very thoughtful and detailed responses. If the referee has ideas of how to improve the paper further, we would be interested to learn them. However, given the significant revisions now implemented, we sincerely hope the referee feels that our work is very close to being suitable for publication.

Referee #3 (Remarks to the Author):

I co-reviewed this manuscript with one of the reviewers who provided the listed reports.

Referee #4 (Remarks to the Author):

I am satisfied that the authors have addressed my comments thoroughly and made the necessary revisions. The manuscript is now acceptable for publication.

Referee #4 (Remarks on code availability):

The code is now clearly commented.

Referee #2 (Remarks to the Author):

I am not satisfied with the authors' reply. As in the previous round, the authors have addressed my criticisms only partially, and the manuscript still contains serious problems.

I did not claim that previous papers studied precisely the same region of the phase diagram. My point was instead that several papers are cited in a way that implies they support the interpretation advanced in this manuscript, whereas in fact they neither support nor contradict it.

The manuscript also continues to make baseless connections to fashionable topics such as FFLO states, fractional vortices, and related phenomena. These references may be appealing to those communities, but the experimental results in this paper do not support nor establish such connections.

Under ordinary circumstances, I would recommend rejection of this paper from any journal. However, the experimental data themselves appear interesting. My recommendation is therefore as follows:

I strongly recommend rejection of the manuscript in its present form from Nature or Nature Physics.
I would not object to publication in a more focused form, provided that the authors remove all non-scientific claims for which there is no experimental or theoretical support in this work.

In light of the referee's comments, we have significantly revised the manuscript, and have sought to do our best to remove all these claims. We have moved the RSJ modelling to the SI, and now the main text focuses firmly on the experimental data.

My specific concerns are listed below.

1. Unsupported interpretation in terms of different vortex species

The central problem remains the authors' interpretation in terms of different species of vortices. This interpretation is based in part on citing a paper on a superfluid mixture, where two independent vortex species exist by construction. That model is not a model of superconductivity and has no relevance to the present system. This reference should be removed.

This is removed, and we have replaced any reference to "vortex species" with "vortex structures" to be more vague and open minded (taking this to allude either to collective behaviour of many vortices or to a single vortex – our data does not give information regarding which, as the referee correctly points out, so we have tried to be much more vague with this revised terminology).

2. No support for two distinct kinds of vortices

There is no theoretical model and no experimental evidence in the manuscript supporting the existence of two distinct kinds of vortices in this superconducting material. I am not aware of

any theory paper on superconductivity that would substantiate this interpretation. The corresponding claim and reference in the abstract should therefore be removed.

This phrasing has been removed.

3. The theoretical modeling is disconnected from the experiment

The theoretical modeling section is not connected to the claimed interpretation in any meaningful way. It does not provide a basis for the central claims made in the manuscript. I strongly insist on removing the theory/modeling part from this paper.

The authors may, of course, publish such a theory separately, but in its present form it is far from sufficient for inclusion in a serious experimental paper in a high-profile journal.

This has all been removed from the main text.

4. The manuscript should remain focused on established experimental facts

If the authors wish, they may keep the cautious suggestion on page 13, beginning with "we propose," that the observed result may be related to the effect they discuss. However, the following paragraph and the discussion based on the RSJ model have no direct relationship to this interpretation and should be removed.

The manuscript should remain focused on well-established facts. In this case, those facts are experimental.

All RSJ modelling has been removed from the main text. All that remains is the paragraph commencing "We propose…" in which we have changed "species" to "structures".

5. Unsupported discussion of diode effects

The references to diode effects on page 10 are not substantiated by the present data and should be removed. The manuscript does not provide sufficient evidence to make this connection.

This is removed.

6. Figure 3 is misleading

Figure 3 is highly misleading because it presents an unsubstantiated theoretical interpretation alongside experimental results. All theoretical components that are not directly supported by the data should be removed. Only experimentally established facts should remain.

This is removed.

7. Fractional vortices are not supported by the data

The references to fractional vortices and the corresponding citations on page 5 should be removed. The present paper has nothing to do with fractional vortices and sheds no light on their existence. Such vortices have not been observed in the other regions of the phase diagram

of this material. The manuscript should focus on the data actually presented, rather than using the results as a pretext to comment on unrelated phenomena.

This discussion has been omitted.

8. FFLO references are unjustified

The references to FFLO physics on page 24 should be removed. The data in this paper have no demonstrated connection to FFLO states.

This is removed.

Overall assessment

The main issue is not that the authors speculate, but that the manuscript repeatedly presents speculation in a way that suggests physical support where none exists. The experimental data is interesting, but the current framing overreaches substantially. The paper attempts to associate the results with a broad range of fashionable topics, including fractional vortices, FFLO physics, diode effects, and related ideas, without providing evidence that these phenomena are relevant.

This is not acceptable for a serious journal, especially not for Nature or Nature Physics. The manuscript could become suitable for publication only after substantial revision, with all unsupported theoretical and interpretive claims removed and the paper refocused on the experimental findings.

We hope the referee finds the new version of the manuscript adequately focussed on experimental facts to be suitable for publication.
